# Expression patterns of small numbers of transcripts from functionally-related pathways predict survival in multiple cancers

**DOI:** 10.1186/s12885-019-5851-6

**Published:** 2019-07-12

**Authors:** Jordan Mandel, Huabo Wang, Daniel P. Normolle, Wei Chen, Qi Yan, Peter C. Lucas, Panayiotis V. Benos, Edward V. Prochownik

**Affiliations:** 1 The Division of Hematology/Oncology, Children’s Hospital of Pittsburgh of UPMC, Children’s Hospital of Pittsburgh of UPMC, Rangos Research Center, Fl. 5, Bay 8, 4401 Penn Ave, Pittsburgh, PA 15224 USA; 2 The Department of Biostatistics and The University of Pittsburgh Graduate School of Public Health, 130 De Soto Street, Pittsburgh, PA 15261 USA; 3 The Hillman Cancer Center of The University of Pittsburgh Medical Center, UPMC, 5150 Centre Ave, Pittsburgh, PA 15232 USA; 4 The Division of Pulmonary Medicine, Allergy and Immunology, Children’s Hospital of Pittsburgh of UPMC, 4401 Penn Ave, Pittsburgh, PA 15224 USA; 5 The Department of Pathology, The University of Pittsburgh Medical Center, S-417 BST 200 Lothrop Street, Pittsburgh, PA 15261 USA; 6 The Department of Computational and Systems Biology, The University of Pittsburgh Medical Center, 3501 Fifth Avenue, 3064 BST3, Pittsburgh, PA 15260 USA; 7 Department of Biomedical Informatics, The University of Pittsburgh Medical Center, 5607 Baum Blvd, Pittsburgh, PA 15206 USA; 8 The Joint Carnegie Mellon-University of Pittsburgh Program in Computational Biology, 3501 Fifth Ave, Pittsburgh, PA 15213 USA; 9 The Pittsburgh Liver Research Center, S414 Biomedical Science Tower, 200 Lothrop Street, Pittsburgh, PA 15224 USA; 10 The Department of Microbiology and Molecular Genetics, 450 Technology Dr. Pittsburgh, Pittsburgh, PA 15219 USA; 11 Division of Hematology/Oncology, Children’s Hospital of Pittsburgh of UPMC, Rangos Research Center, 4401 Penn Ave, Pittsburgh, PA 15224 USA

**Keywords:** Cancer metabolism, Myc, Notch, PI3 kinase, TP53, Transcriptional profiling, T-SNE, Wnt

## Abstract

**Background:**

Genetic profiling of cancers for variations in copy number, structure or expression of certain genes has improved diagnosis, risk-stratification and therapeutic decision-making. However the tumor-restricted nature of these changes limits their application to certain cancer types or sub-types. Tests with broader prognostic capabilities are lacking.

**Methods:**

Using RNAseq data from 10,227 tumors in The Cancer Genome Atlas (TCGA), we evaluated 212 protein-coding transcripts from 12 cancer-related pathways. We employed *t-*distributed stochastic neighbor embedding *(t-*SNE) to identify expression pattern difference among each pathway’s transcripts. We have previously used t-SNE to show that survival in some cancers correlates with expression patterns of transcripts encoding ribosomal proteins and enzymes for cholesterol biosynthesis and fatty acid oxidation.

**Results:**

Using the above 212 transcripts, t-SNE-assisted transcript pattern profiling identified patient cohorts with significant survival differences in 30 of 34 different cancer types comprising 9350 tumors (91.4% of all TCGA cases). Small subsets of each pathway’s transcripts, comprising no more than 50–60 from the original group, played particularly prominent roles in determining overall t-SNE patterns. In several cases, further refinements in long-term survival could be achieved by sequential t-SNE profiling with two pathways’ transcripts, by a combination of t-SNE plus whole transcriptome profiling or by employing t-SNE on immuno-histochemically defined breast cancer subtypes. In two cancer types, individuals with Stage IV disease at presentation could be readily subdivided into groups with highly significant survival differences based on t-SNE-based tumor sub-classification.

**Conclusions:**

t-SNE-assisted profiling of a small number of transcripts allows the prediction of long-term survival across multiple cancer types.

**Electronic supplementary material:**

The online version of this article (10.1186/s12885-019-5851-6) contains supplementary material, which is available to authorized users.

## Background

Molecular genetic advances, particularly next-generation DNA and RNA sequencing, have identified gene copy number variations, recurrent mutations, rearrangements and transcript expression differences in many cancers. These may be associated with specific tumor subtypes, biological behaviors, therapeutic responses and outcomes not otherwise revealed by more traditional histologic or immuno-histochemical assessments [1–5]. However, such tests tend to focus upon and be of value for only specific cancer types or subtypes and are generally not more broadly applicable. There is thus a clear need to assess these parameters more globally and across multiple cancers with a common and preferably small set of genes. The availability of such a test could greatly simplify and expand the molecular evaluation of cancers, further improve prognostication and therapeutic stratification and aid in decisions regarding the frequency and intensity of post-therapy follow-up.

Using the machine learning algorithm “*t-*distributed stochastic neighbor embedding” (*t*-SNE) [6] we have previously demonstrated that the expression patterns of ribosomal protein transcripts (RPTs) differ among normal tissues and cancers in distinct and reproducible ways that are largely independent of absolute expression levels [7, 8]. Most cancers contained two-five distinct RPT t-SNE expression patterns (“clusters”) and in seven cancer types these correlated with long-term survival [8]. More recently, we made similar observations with transcripts encoding enzymes involved in cholesterol biosynthesis and fatty acid oxidation (FAO) [9].

Ribosomal biogenesis, cholesterol biosynthesis and FAO are only three of numerous growth- and metabolism-related pathways that are de-regulated in cancer [8–11]. To investigate whether other transcripts are similarly informative of long-term survival, we used t-SNE-assisted clustering to ascertain the expression patterns of 212 protein-coding transcripts from twelve additional cancer-related pathways. We found these patterns to be predictive of survival for 30 of the 34 distinct cancers, comprising 91.4% of the 10,227 tumors from The Cancer Genome Atlas’ (TCGA) PanCancer Atlas collection [12].

## Methods

### Selection of pathways and RNAseq data

Transcripts for 8 of the 12 cancer-related pathways listed in Additional file 1: Table S1 were obtained from Sanchez et al. [11]. Additional transcripts encoding enzymes of the Purine and Pyrimidine Biosynthetic Pathways and Pentose Phosphate Pathway were selected because of their established roles in providing critical anabolic precursors for nucleic acid synthesis [12–14]. TCA Cycle transcripts were selected because oxidative phosphorylation is often altered in cancer cells as they reprogram glucose, fatty acid and glutamine metabolism [15]. RNAseq expression data (FPKM-UQ) were taken from the TCGA GDC PANCAN dataset and accessed through the UCSC Xenabrowser [16]. These represent RNAseq results from 10,227 untreated primary tumors. The only exception to this was uveal melanomas where all tumors were metastatic (SI Appendix Table S2). Expression values were initially stored as the base-two logarithm of the incremented-by-one FPKM-UQ value. The inverse of this transformation was applied to the values to obtain the true FPKM-UQ values.

### Depiction of cancer pathway transcript patterns

Prior to t-SNE visualization, RNA expression data were centered and normalized for each pathway [8]. Briefly, every primary tumor sample was assigned an “expression vector” in n-dimensional space for each pathway, where n was equal to the number of genes in the pathway and each element of each vector was equal to the FPKM-UQ expression value of a particular gene. For each pathway-cancer type combination, the associated expression vectors were centered by subtracting from each one the mean value of all the vectors. The centered vectors were then normalized by their magnitudes. The result was that all centered expression vectors were projected onto a hyper-sphere in n-dimensional space. For each cancer type and pathway, the vectors on this hypersphere were the input to t-SNE. t-SNE analyses of each pathway’s transcript patterns were performed using Tensorboard Release 1.12.0 [17] in three dimensions to maximize the appreciation of the compactness and separateness of the resulting clusters. Multiple t-SNE runs were initially attempted with perplexities ranging between 5 and 30, and learning rates of 0.1, 1, 10, or 100. For each cancer-pathway combination, parameters that produced obviously distinguishable clusters were selected for further validation by multiple runs. Cancer-pathway combinations for which no set of parameters could be found that produced obviously distinguishable clusters were rejected for further analysis. We heuristically defined an obvious cluster as a densely distributed collection of points in the embedding space separated from other such dense regions by clearly discernable regions containing no embedded points. For the final selected parameters, t-SNE was run for at least 2500 iterations and until the embedding stabilized. After embedding, the number of clusters was recorded. Members of the clusters were specified using Gaussian mixture models implemented through MATLAB’s “fitgmdist” and ‘cluster’ functions. Though a Gaussian mixture models were used to assign samples to clusters we refer to the clusters as “t-SNE clusters”. The default “K-means++” algorithm was used to set initial conditions in all cases. In some cases, the output t-SNE data were randomly perturbed by 5% of the radius of the smallest sphere that contained all the output points before clustering. The number of Gaussian components used was equal to the number of clusters previously identified. For each t-SNE profile, every combination of full or diagonal covariance matrices, shared or unshared covariance and the application or non-application of the aforementioned perturbation were iteratively tried when fitting the Gaussian mixture model, for a total of eight attempts with different parameter settings. The output that best preserved the unity of the obviously distinguishable clusters in the t-SNE were chosen for display in all figures and for further analysis. Finally, the aforementioned perturbation was applied to the actual output t-SNE scatterplot displayed in the figures in cases where clusters were so dense as to prevent its individual component members from being readily visualized. The parameters used for each tSNE and Gaussian mixture clustering are listed in Additional file 1: Table S3. Parametric t-SNE [18] was used to confirm the clusters found with the initial t-SNE assisted clustering, using the same perplexity as in that initial analysis, where three-quarters of the data were used for training and one quarter was withheld as a test set.

### Comparing t-SNE clusters

Clinical and survival data for TCGA cancer cohorts were accessed using the UCSC Xenabrowser [16] under the data heading “Phenotypes”. Kaplan-Meier survival curves for each each *t*-SNE cluster were compared using Mantel-Haenszel (log-rank) methods through the “Matsurv” function on the MATLAB file exchange [19] and confirmed in Graphpad Prism 7.

### Random forest analyses

To identify genetic features that differed the most among t-SNE clusters, a random forest classifier model [20, 21] was employed through MATLAB’s ‘TreeBagger’ function in the ‘Statistics and Machine Learning Toolbox’, with ‘NumTrees’ equal to 100, ‘OOBPredictorImportance’ turned on, ‘NumPredictorsToSample’ set to ‘all’, and ‘PredictorSelection’ set to ‘interaction-curvature’. The importance of the transcripts in distinguishing the clusters from one another were indicated by the ‘OOBPermutedPredictor’ field of the object returned by the ‘TreeBagger’ function.

### Comparing t-SNE clusters with hierarchical groups

To investigate relationships between t-SNE clusters and the entire expressed protein-coding genome, four cancer types were selected for full transcriptome visualization by hierarchically clustered heat maps. RNAseq-based heat maps of the cancers of interest were downloaded from the TCGA Next-Generation Heat Map Compendium [22]. We selected the platform “RNA Expression” and heat map type selected as “Gene/Probe vs Sample”. Tumor and t-SNE samples represented in this heat map had significant overlap. Samples were pre-divided into hierarchical groups (hereafter referred to as “Dendros” to avoid confusion with the t-SNE clusters). Individual tumors within each Dendro were then identified according to the t-SNE clusters with which they associated. Significance of survival differences between these groups within each Dendro was assessed in Graphpad Prism 7 using log-rank tests.

## Results

### Transcript patterns from cancer-related pathways correlate with survival

**Fig. 1:**
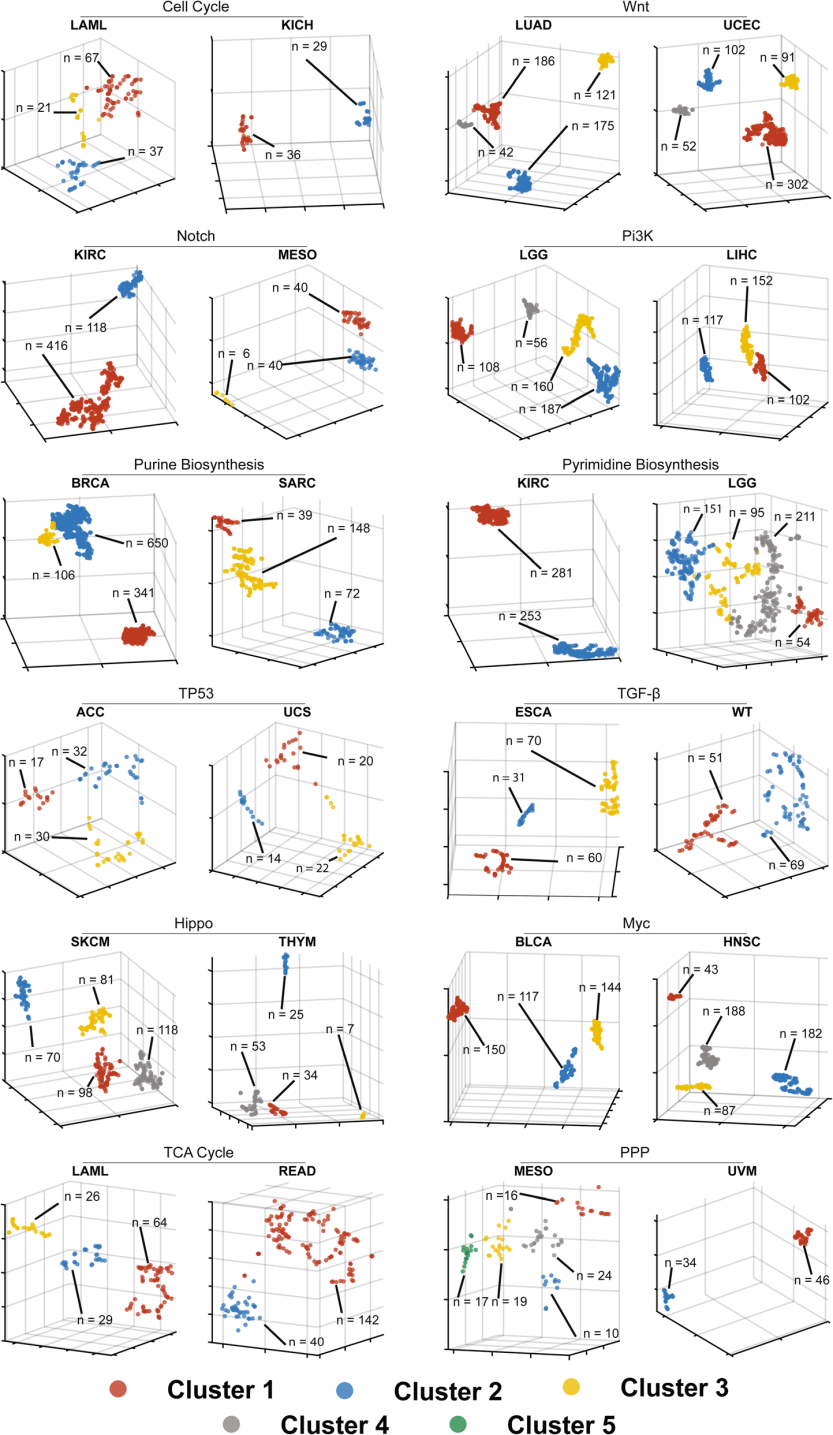
3D t-SNE plots of transcript clusters from each of the 12 cancer-related pathways. For each pathway (Additional file 1: Table S1), two representative tumor types are shown. See Additional File 1: Table S3 in the Supplementary Appendix for the specific parameters used to generate each t-SNE cluster. Additional file 1: Figures S1-S12 show t-SNE profiles of additional relevant tumor types for each pathway. Axes here and in Additional file 1: Figures S1-S12 are unlabeled as t-SNE parameters are non-linear, dimensionless and not meaningfully interpretable (49)

Our previous finding that the expression patterns of transcripts encoding ribosomal proteins and enzymes involved in cholesterol biosynthesis and FAO pathways raised the question of whether t-SNE patterns of other transcript families also correlated with survival [7–9]. We therefore assembled a core group of 212 transcripts representing 12 pathways with well-defined roles in cancer cell proliferation, survival and metabolism and whose members are frequently subject to mutations that drive cancer pathogenesis (Additional file 1: Table S1) [13–17, 19]. In 10,227 samples from TCGA representing 34 cancer types, t-SNE profiling identified distinct and multiple tumor type-specific transcript patterns for most pathways (Fig. 1 and Additional file 1 Figures S1–12).

**Fig. 2:**
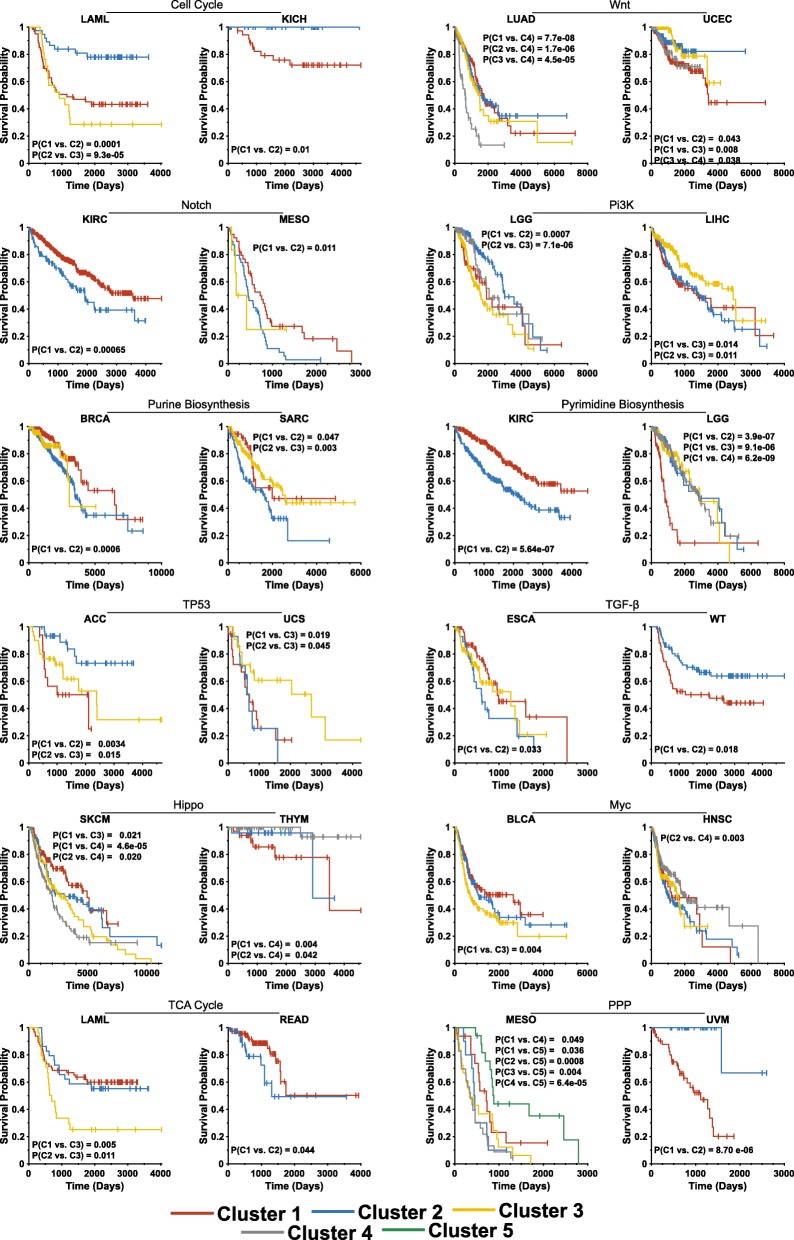
Kaplan-Meier survival curves of patients based on t-SNE profiles. The survival curves shown here are those for the t-SNE clusters in Fig. 1. Patient groups are indicated by the same colors used to present the t-SNE clusters. *P* values between individual groups are indicated only when significant. See Additional file 1: Figures S13-S24 for other relevant survival curves that correspond to the t-SNE profiles depicted in Additional file 1: Figures S1–12

**Fig. 3:**
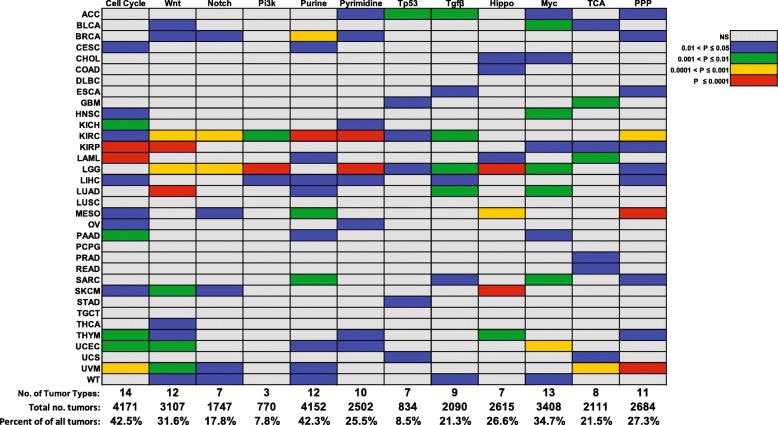
Summary of Kaplan-Meier survival results for every tumor type. The results are summarized from Fig. 2 and Additional file 1: Figures S13–24 in the Supplementary Appendix. Colored boxes indicate instances in which the overall survival of at least one patient t-SNE cohort differed significantly from at least one other cohort. Grey boxes indicate cancers where survival differences between individual t-SNE clusters groups were not significant or where only a single t-SNE cluster was obtained. The P values listed are those between the two most disparate sets of survival curves for each comparison (Figs. 2 and Additional file 1: Figures S13-S24)

The t-SNE clusters of individual pathways correlated with survival in 3–14 cancer types comprising 9.6–38.9% of the entire TCGA population (Figs. 2 and 3 and Additional file 1: Figures S13–14). Collectively, survival for all cancers for which t-SNE-assisted clustering was useful could be predicted from a mean of 3.7 pathways. This ranged from nine pathways for low-grade gliomas and clear cell kidney cancer to as few as a single pathway each for colon, prostate and colo-rectal cancers (Fig. 3). t-SNE-assisted clustering did not predict survival in diffuse large B-cell lymphoma, squamous cell lung cancer, phenochromocytoma/paraganglioneoroma and testicular germ cell tumor, collectively comprising 8.6% of the TCGA population. t-SNE patterns from at least one pathway, and more often multiple pathways, thus predicted survival in 30 of 34 cancer groups, comprising 91.4% of all tumors (Fig. 3).

Certain RPT transcripts disproportionately and recurrently shape t-SNE clusters [8, 9]. We therefore applied a Random Forest classifier [20] to identify such key transcripts in each of the above 12 pathways. These were relatively few in number, ranging from as few as one-two to as many as four-six depending both on the tumor type and the specific pathway (Additional file 1: Figures S25–36). Thus, a much smaller subset of the original 212 transcript collection, comprising no more than 50–60 members, contributed disproportionately to the t-SNE profiles of most cancers.

### Additional predictive value from sequential t-SNE analysis and whole transcriptome profiling

**Fig. 4:**
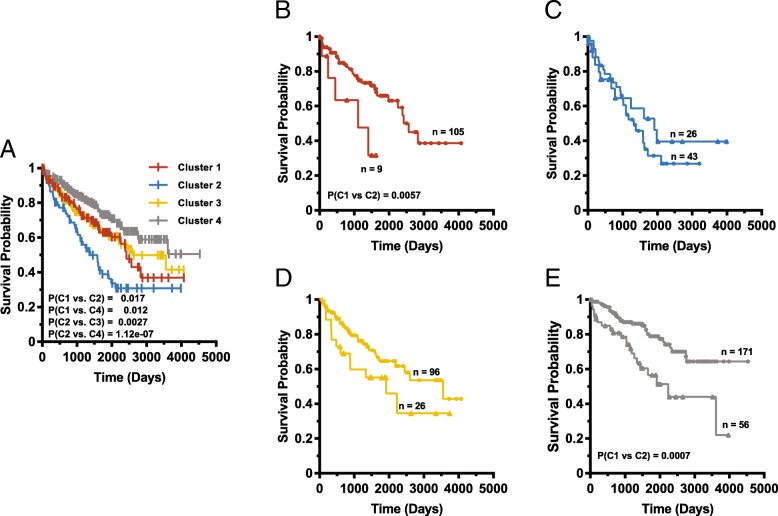
Additional predictive power of sequential t-SNE analyses. **a**. The survival curves of clear cell kidney cancer patients based on t-SNE clusters from the Purine Biosynthesis Pathway taken from Additional file 1: Figure S17. **b**. Cluster 1 patients from (**a**) were further analyzed based on whether they could be categorized as Cluster 1 or Cluster 2 when analyzed for Notch Pathway transcripts. **c-e** Clusters 2–4 patients from (**a**) were similarly categorized as in (**b**). See Additional file 1: Figures S37 and S38 for additional examples

Because t-SNE profiles for more than one pathway correlated with survival in 25 of 34 cancers (Fig. 3) we asked whether a second, sequential analysis performed on an initial set of t-SNE clusters could be used to further refine survival predictions. Figure 4a shows the original Kaplan-Meier survival curves of the four patient cohorts with clear cell kidney cancer profiled with Purine Biosynthesis Pathway transcripts (Additional file 1 Figure S17). Subsequent t-SNE-assisted profiling with Notch Pathway members allowed for additional subdivisions of two of these groups. Cluster 1, with relatively poor prognosis (median survival = 2419 days), could be sub-divided into a large sub-group with slightly longer median survival (2564 days) and a smaller sub-group with a particularly poor median survival of only 1111 days (*P* = 0.0057) (Fig. 4b). Neither Clusters 2 nor 3 could be further subdivided (Fig. 4c and d). Similarly, Cluster 4, with the best overall survival of > 3700 days, could be subdivided into two groups with median survivals of > 4700 days and 2241 days, respectively (*P* = 0.00045) (Fig. 4e). At least two additional examples of initial t-SNE clusters (generated from sarcomas and head and neck squamous cell cancers) that could be sub-classified with a second pathway’s transcripts are shown in Additional file 1 Figures S37 & 38).

**Fig. 5:**
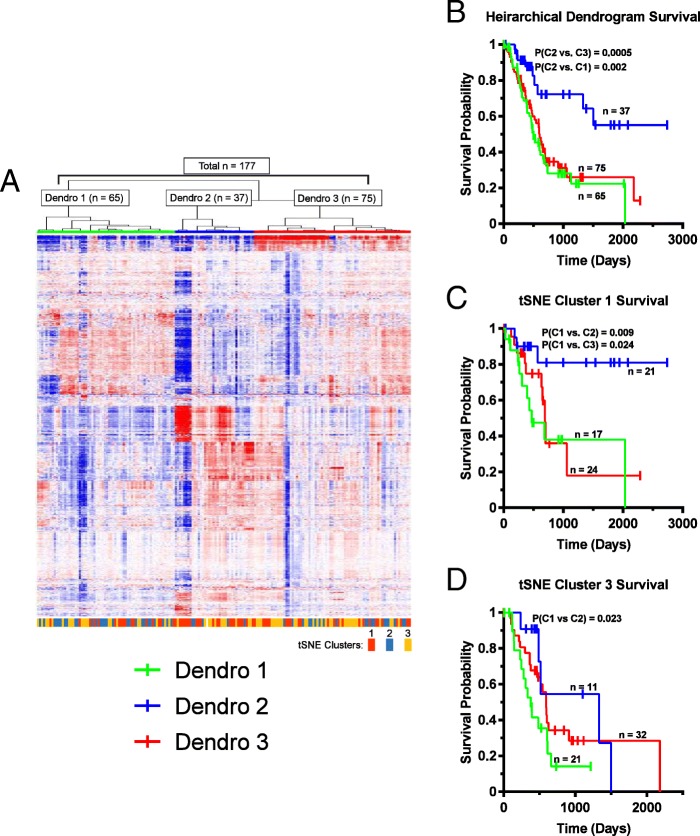
Whole transcriptome analysis further refines the predictive power of t-SNE profiling. **a**. Unsupervised hierarchical clustering of whole transcriptome profiles from 177 pancreatic adenocarcinomas (22). Three major groups were identified (Dendro 1, Dendro 2, and Dendro 3) and are indicated by the green, blue and red horizontal bars, respectively, at the topv of the heat map. Within each Dendro group, individual tumors, previously classified by t-SNE for their expression patterns of purine biosynthesis family transcripts (Clusters 1–3: Additional file 1: Figure S5) are indicated by the red, blue and yellow-colored bars, respectively, at the bottom of the heat map. **b**. Kaplan-Meier survival curves of patients from each of the Dendro groups in A. **c**. Tumors from Purine Biosynthesis Pathway t-SNE Cluster 3 (unfavorable survival) were further divided according to the dendrogram group with which they associated and Kaplan-Meier curves were again generated. **d**. Similar to (**c***)*, patients from Purine Biosynthesis Pathway t-SNE Cluster 1 (favorable survival) were also grouped according to the Dendro group with which they associated. See Additional file 1: Figures S39-S41 for additional examples of this type of analysis

Whole transcriptome profiling can molecularly classify tumors and predict survival and therapeutic responses [23, 24]. To determine whether t-SNE-assisted clustering could also be employed to further refine survival predictions based on this approach or vice versa, we retrieved RNAseq data from several tumor types, generated heat maps of all expressed protein-coding transcripts and sub-classified tumors using hierarchical clustering [22, 25]. We initially focused on pancreatic ductal adenocarcinoma in which t-SNE analysis with Purine Biosynthesis Pathway transcripts had previously identified three distinct cohorts with differential survival (Additional file 1 Figures S5 and S17). Hierarchical clustering revealed three molecular subgroups (Fig. 5a), two of which, “Dendro 1” and “Dendro 3”, were associated with inferior survival (Fig. 5b). Tumors from the three t-SNE clusters were about evenly distributed among these Dendro groups (Fig. 5a). t-SNE Cluster 1 tumors could be further subdivided into groups with significant differences in survival based upon their dendrogram identities (Fig. 5c). Similarly, t-SNE Cluster 3 tumors could also be divided into groups with significant differences in survival (Fig. 5d). Thus, t-SNE clusters, already predictive of survival, could be further stratified based on hierarchical clustering. Similarly, dendrogram groups contained patients whose survival could be further stratified based on t-SNE profiles.

Related findings were made in clear cell kidney cancer, where whole transcriptome profiling generated 4 dendrograms (Dendro 1–4) with Dendro 1 having particularly unfavorable survival (Additional file 1 Figure S39*A&B*). Unlike the more random distribution of t-SNE clusters seen in Fig. 5a, Dendro 1 group was overly populated by Pyrimidine Biosynthetic Pathway t-SNE Cluster 2 tumors (also with unfavorable outcomes-Fig. 2) whereas the Dendro 3 group with a favorable outcome contained a preponderance of t-SNE 1 cluster tumors, also with more favorable outcomes. However, both t-SNE groups could be further sub-divided into distinct survival cohorts when categorized by their respective dendro group (Additional file 1 Figure S39 *C&D*). Additional variations of these general themes were seen with Myc Pathway transcripts in sarcomas and TCA Cycle Pathway transcripts in Bladder Cancer (Additional file 1: Figures S40 & S41). t-SNE-based analysis is thus comparable and in some cases even superior to whole transcriptome profiling for forecasting long-term survival. As with sequential t-SNE profiling, the two methods can be used in tandem to better define tumor subgroups and long-term survival.

### T-SNE compliments sub-classification and clinical staging for certain cancers

Triple-negative breast cancer (TNBC), which represents 10–20% of all tumors, is defined by the lack of immuno-histochemical staining for the estrogen and progesterone receptors and the cell surface epidermal growth factor receptor *HER2*. It has the most unfavorable outcome of all breast cancer subtypes due primarily to its propensity for early metastatic recurrence [26, 27]. In contrast, the Luminal A form, representing 50–60% of all cases, has the most favorable long-term survival [28, 29]. Belying the apparent simplicity of this long-standing classification scheme, however, is the fact that TNBC and Luminal A variants have each been recently sub-classified into several distinct molecular entities based on whole transcriptomic profiling [26, 27, 30, 31].

**Fig. 6:**
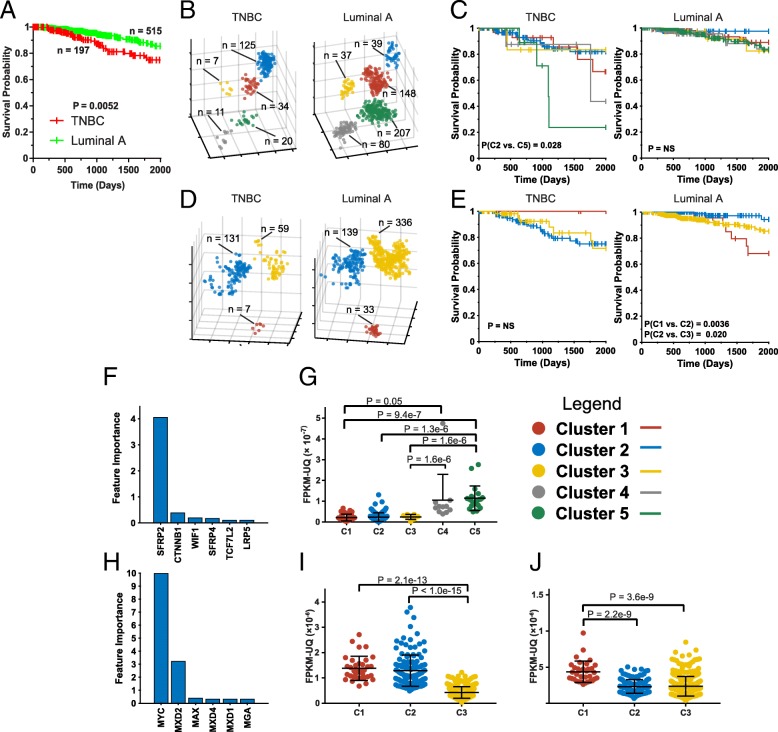
t-SNE profiling can further refine survival prediction in specific breast cancer subtypes. **a** Kaplan-Meier survival of patients with TNBC and Luminal A tumors. Patients and survival information were compiled from TCGA and ref. 23. **b** t-SNE clusters of only TNBC and Luminal A tumors from (**a**) using Wnt Pathway transcripts. These were derived from Additional file 1: Figure S2. **c**. Kaplan-Meier survival of each of the t-SNE groups from (**b**). NS = not significant (**d**). t-SNE profiling of TNBC and Luminal A tumors using Myc Pathway transcripts. (**e**). Kaplan-Meier survival of each of the t-SNE groups from (**d**). **f** Random Forest classification of transcripts from the Wnt Pathway that were the most deterministic of survival for all TNBC patients from (**a**). **g** Expression levels of Sfrp2 transcripts in each of the t-SNE clusters of TNBCs from (**b**). **h**. Random Forest classification of transcripts from the Myc Pathway that were the most deterministic of survival for all Luminal A patients from (***a***). **i**. Expression levels of Myc transcripts in each of the t-SNE clusters of Luminal A tumors from (**d**). **j**. Expression levels of Mxd2 transcripts in each of the t-SNE clusters of Luminal A tumors from (**d**)

To determine whether t-SNE-based analyses could aid in refining the survival prediction for these two forms of breast cancer, we first confirmed these differences using data from the TCGA database and ref. 23 (Fig. 6a). Because Wnt Pathway transcript t-SNE patterns had been predictive of survival in all breast cancer patients (Fig. 3, and Additional file 1 Figures S2 and S14), we applied these analyses to the individual TNBC and Luminal A subtype populations. TNBCs comprised 17.9% of all tumors (197 of 1097) and occupied the same original five t-SNE clusters as their non-TNBC counterparts (Fig. 6b). However, these tumors were disproportionately grouped into Cluster 2, which contained 62.8% of the total TNBC population (*P* = 4.2 × 10^− 60^ based on Fisher’s exact test), with the remaining four clusters each containing 5.3–11%. Luminal A cancers (46.5% of all tumors) were evenly distributed among t-SNE clusters 1,3,4 and 5 (48–56.3%) but were relatively depleted from Cluster 2 (19.5%. *P* = 4.37 × 10^− 18^). Thus, Cluster 2 was disproportionately comprised of a relative excess of TNBCs and a paucity of luminal A cancers. As a group, this Cluster’s survival was identical to that of Clusters 1,3 and 4 whereas the smaller number of TNBCs within Cluster 5 (20/197 = 10.1%) was associated with a significantly worse long-term survival (Fig. 6c). Wnt pathway transcript patterns were not predictive of survival for luminal A cancers.

t-SNE-based profiling of breast cancers with Myc Pathway member transcripts did not initially identify groups with significantly different survival (Fig. 3). However, the analysis of Luminal A tumors but not TNBCs with this pathway’s transcripts did further enhance survival prediction (Fig. 6d&e). Taken together, these results demonstrate that, at least in the case of breast cancer, well-defined molecular subtypes could be further categorized by the subsequent interrogation with t-SNE-based transcriptional profiling.

On average, Random Forest classification had shown that approximately three Wnt Pathway transcripts were the major determinants of t-SNE cluster profiles among the 12 different cancer types, including all breast cancers, where differential survival among Clusters was observed (Fig. 3). The most prominent of these transcripts were Sfrp2, Ctnnb1 and Dkk1/3 (Feature Importance > 1, Additional file 1 Figure S26). In the case of TNBC, however, this patterning was determined exclusively by Sfrp2 (Fig. 6f). Consistent with this, Cluster 5 tumors expressed the highest levels of Sfrp2 transcripts (Fig. 6g).

t-SNE clusters generated by Myc Pathway transcripts in 11 relevant tumor types were also determined by an average of three transcripts/tumor type with the most common ones being Myc, N-Myc and Mxd2 (Additional file 1 Figure S34). The t-SNE clusters of Luminal A cancers, in contrast, were more driven by Myc and Mxd2 (Fig. 6h). Interestingly, the Cluster 1 tumors of this subset, which expressed high levels of Myc and Mxd2 were associated with the worst prognosis (Fig. 6i&j).

**Fig. 7:**
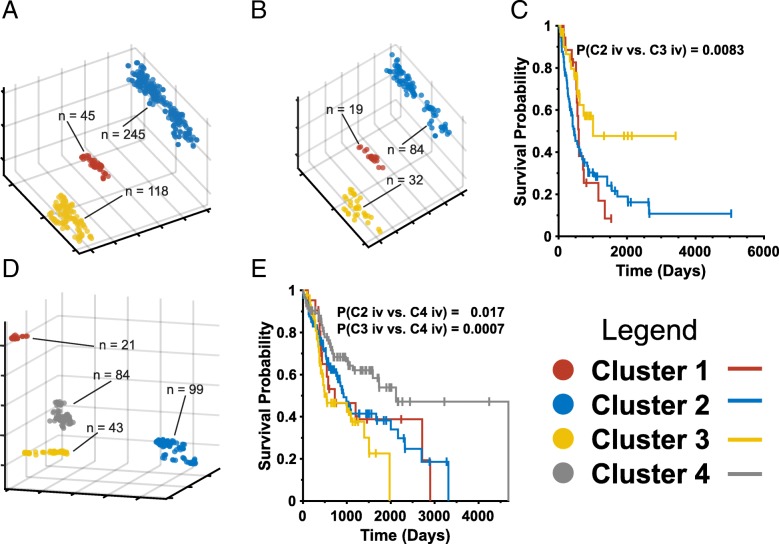
t-SNE profiling better predicts survival in tumors from individuals with advanced stage disease. (**a**). Original t-SNE clusters of all primary bladder cancers profiled with TCA Cycle transcripts (from Additional file 1: Figure S11). **b** The t-SNE clusters from (**a**) showing only Stage IV primary tumors (total = 135). ***c*** Differential survival of Stage IV patients from (**b**). **d**. t-SNE clustering of Stage IV only head and neck squamous cell cancers using Myc Pathway transcripts. See Fig. 1 for t-SNE clustering with all tumors. **e** Survival of patients from (**d**) according to t-SNE cluster

Lastly, we asked whether the survival of patients with advanced stage disease at the time of diagnosis could also be better stratified by t-SNE analysis. To this end, we re-analyzed the bladder cancers in TCGA (SI Appendix Table S2), 135 of which originated from patients with Stage IV disease. A Chi-square test indicated that the tumors were randomly distributed among the three previously identified t-SNE clusters (*P* = 0.073), Fig. 7a*&*b and Additional file 1 Figure S11). Just as t-SNE profiling had previously predicted differential survival in all patients with bladder cancer (Additional file 1 Figure S23), so too was it predictive of survival in individuals with Stage IV tumors with Cluster 3 tumors being associated with significantly more favorable survival (Fig. 7c).

Similar findings were made in head and neck squamous cell cancers (SI Appendix Table S20 where t-SNE profiling with Myc Pathway transcripts had previously identified four distinct clusters with significant survival differences (Figs. 1 and 2). As with bladder cancers, the primary tumors from 247 Stage IV cancers were randomly distributed among these groups (*P* = 0.075, Fig. 7d). Among these tumors, however, t-SNE Cluster 4 was associated with a significantly longer median survival (2120 days) than the other clusters (combined median survival = 915 days).

## Discussion

Molecular tests such as MammaPrint™ and ThyroSeq™ have proven highly useful in guiding the diagnosis and prognosis of select cancers [4, 5]. In the former case, a 70 gene expression signature in Stage I and II breast cancer can accurately predict the likelihood of recurrence following surgical extirpation and thus inform the need for adjuvant chemotherapy [4]. ThryoSeq™ utilizes a collection of ~ 140 gene copy number variations, fusions and transcript expression differences to diagnose and classify malignant thyroid nodules of indeterminate histology [5]. Despite their utility, these tests are relevant only to their respective tumor types or, more specifically, certain stages or subtypes and lack broader applicability.

We have demonstrated here the feasibility of predicting survival in multiple cancer types based on the expression patterns of small, functionally related subsets of a 212 member transcript collection. These were drawn from 12 canonical pathways with well-established roles in cancer cell proliferation, survival and metabolism [11–15, 25, 32, 33]. However, unlike whole transcriptome profiling where gene expression levels correlate with survival in specific cancers (Fig. 5a, Additional file 1 Figures S39-S41) [22], the value of the analyses reported here lies in the expression patterns of small numbers of transcripts across multiple tumor types. Indeed, in 30 of 34 cancers, these patterns were so highly predictive of survival that transcripts from a single pathway sufficed for this purpose. Examples include the Cell Cycle Pathway (15 transcripts) in acute myelogenous leukemia, the PI3K Pathway (18 transcripts) in low-grade gliomas and any one of nine pathways, each comprised of 6–30 transcripts, in clear cell kidney cancer (Fig. 3). Indeed, of the 30 cancer types for which t-SNE-assisted profiling was useful, an average of 3.7 pathways/tumor type correlated with survival, thus providing coverage of 91.4% of all cancers archived in TCGA. Our previous t-SNE profiling with transcripts encoding ribosomal proteins and enzymes involved in cholesterol biosynthesis and FAO [7–9] was also prognostic for 17 of the listed cancers and also did not include any of the four not covered by the current 12 pathways (Fig. 3). The future addition of new pathways may eventually prove to be of prognostic value in these four cancer types. It is worth considering the possibility that the failure of this approach in testicular germ cell tumor may reflect this cancer’s extraordinarily high cure rate [34]. For these reasons, the current numbers must be considered provisional and likely to expand. The precise fraction of cancers for which t-SNE profiling will prove useful is also likely to change somewhat given that the TCGA database is biased both for and against particular cancer types (for example, it excludes many rare cancers and most pediatric cancers).

Unsurprisingly, many of above pathways’ transcripts encode known oncoproteins and tumor suppressors such as Myc, PTEN, p53 and IDH1/2 whose mutation, expression level and/or de-regulation frequently correlate with various individual cancers and their outcomes (SI Appendix Table S1) [11, 35–41]. However, we show that an additional and more powerful prognostic aspect of these transcripts resides in the patterns they assume relative to other transcripts in their respective pathways. These patterns likely serve as surrogate reporters for the unique transcriptional and post-transcriptional environments that characterize each cancer type and that dictate its relevant behaviors in much the same way as does whole transcriptome hierarchical clustering [42–44]. Such patterns are undoubtedly determined by numerous interdependent factors including chromatin conformation; the binding and activities of promoter-proximal complexes such as RNA polymerase II and Mediator; the binding and activities of adjacent transcriptional factors; the long-range contribution of protein-bound enhancers and super-enhancers and the regulation of all these by post-translational modifications, metabolites and additional tissue-specific proteins [42, 45–48]. Differences in mRNA splicing and stability further influence mature transcript expression levels in tissue- and tumor-specific ways [49, 50]. That transcript patterns may reflect a more complex control than do absolute levels is suggested by the fact that, in at least some cases, these two do not correlate (Fig. 5a). Based on presumably similar regulatory dependencies, it seems likely that t-SNE patterns will also correlate with other important tumor behaviors such as therapeutic responses and their durability.

Also to be emphasized is that the entire 212 transcript repertoire reported here is unnecessary for assessing any individual tumor type. Rather, only those pathways of previously demonstrated predictive value for a particular tumor type need be selected (Additional file 1 Figures S25–36). In the case of low-grade gliomas and clear cell renal cancer, for example, this could be as many as nine pathways or as few as a single one for colo-rectal and prostate cancers (Fig. 3).

In some cases, additional prognostic information was extracted using sequential t-SNE analysis or whole transcriptome profiling (Figs. 4 and 5 and Additional file 1 Figures S37-S41). It is in tumor types such as pancreatic ductal adenocarcinoma where particular t-SNE profiles are more evenly distributed across the entire transcriptome spectrum that the combined advantages of these two independent approaches are likely to have the greatest impact (Fig. 5).

Importantly, more traditional and clinically well-integrated ways of classifying tumor can also be complemented using the t-SNE-based profiling described here so as to allow for the identification of more or less challenging tumor subsets. This was well-illustrated for breast cancer where the TNBC and Luminal A subtypes, already long-known as having distinct outcomes [23, 24, 26–29], could both be subdivided, albeit with different set of transcripts (Fig. 6). The high-risk TNBC group was particularly interesting as these patients’ t-SNE profiles and their long-term survival, were entirely driven by the expression of Sfrp2 (Fig. 6b,c & g). Sfrp2 (“secreted frizzled-related protein”) is a cell surface protein that is highly expressed by breast cancer-associated endothelial cells and correlates inversely with survival [51]. Monoclonal antibody-mediated inhibition of Sfrp2 has been show to reduce tumor growth and prolong survival in a mouse model of TNBC [52]. This suggests the intriguing possibility that some transcripts that are predictive of survival may not necessarily be expressed by actual tumor cells but rather by stromal elements that play critical roles in maintaining tumor growth, nutrition and oxygenation [53].

For both bladder and head and neck squamous cell cancer, t-SNE profiling also complemented and strengthened the well-recognized prognostic power of classical clinical staging, which is largely predicated on well-established clinico-pathologic criteria such as tumor size and location, local invasion, lymphatic involvement and distant metastatic spread, with the latter being indicative of the most advanced, i.e. Stage IV, disease [54]. The fact that the t-SNE clusters of stage IV tumors were indistinguishable from those of their less advanced counterparts (Fig. 7b&d) argues that, rather than simply being correlated with and perhaps the result of more advanced disease, transcript expression patterns are a fundamental property of their respective tumor that likely precedes the onset of metastatic dissemination. More extensive testing involving additional cancers and transcript pathways will be required to determine how t-SNE-based analyses can best be integrated with these more traditional types of evaluation so as to establish the best clinical practice.

It is important to emphasize that, like all other clinic-pathologic, biochemical and molecular analyses, the results generated by t-SNE profiling must be interpreted cautiously and in light of other factors that are not necessarily accounted for by the analysis itself and that, either individually or together, could affect long-term outcomes. These might include such non-mutually exclusive factors as age and frailty, co-existing organ dysfunction that limits chemotherapy dosing, disparities in the quality of care or the inability to initiate or continue treatment.

## Conclusions

Collectively, our results demonstrate that the expression patterns rather than the absolute levels of small, functionally related sets of transcripts can be used to achieve highly accurate projections of long-term survival in the vast majority of cancer patients. In most instances, several different pathways can be selected for the analysis of any particular tumor type. Together, the pathways can be used to predict survival in at least three and as many as 14 different tumor types for which the approach is applicable. Additional versatility is demonstrated by the fact that tandem t-SNE profiling or t-SNE profiling in conjunction with whole transcriptome analysis affords even greater refinement of survival prediction. This remains true when t-SNE profiling is combined with more traditional forms of tumor assessment such as immuno-histochemical staining and clinic-pathologic staging. Future efforts should continue to focus on and improve the benefits offered by such combinatorial analyses. While the prognostic advantages of these sequential approaches may initially be somewhat limited in their statistical power by relatively small patient numbers, this is likely to diminish with the accrual of additional data.

## Additional file

Additional file 1: **Table S1.** Component Transcripts and NCBI Gene ID Numbers Used for t-SNE Profiling in Each of Twelve Cancer-Related Pathways. Note that, although there are a total of 221 transcripts listed, 9 of those in the Purine and Pyrimidine Biosynthesis Pathways (depicted in bold) are common. Thus, a total of 212 unique transcripts were used for generating t-SNE profiles. **Table S2.** Abbreviations for and Number of Cancers in Each of the TCGA Groups and Those for Which Survival Data is Unavailable. **Table S3.** t-SNE clustering parameters. For “Diagonal” covariance matrices only the diagonal entries were non-zero, and the principle axes of the fitted Gaussians were parallel to the X,Y, and Z axes. For “Full” covariance matrices any entry could be nonzero and the principle axes of the fitted Gaussians could be oriented in any direction. Shared Covariance: in cases where this is “TRUE” each fitted Gaussian had the same covariance matrix. Where this was “FALSE” every fitted Gaussian had a unique covariance matrix. Perturb Input: where this is “TRUE” the t-SNE data were randomly perturbed by a maximum of 5% of the radius of the sphere enclosing them prior to clustering. Perturb Output: Where this is “TRUE”, the t-SNE scatter-plots displayed in the figures have the afore-mentioned perturbation applied. **Figure S1.** Additional t-SNE profiles for select tumor types, excluding those shown in Fig. 1, demonstrating Cell Cycle Pathway transcript clustering. **Figure S2.** Additional t-SNE profiles for select tumor types, excluding those shown in Fig. 1, demonstrating Wnt Pathway transcript clustering. **Figure S3.** Additional t-SNE profiles for select tumor types, excluding those shown in Fig. 1, demonstrating Notch Pathway transcript clustering. **Figure S4.** Additional t-SNE profiles for select tumor types, excluding those shown in Fig. 1, demonstrating PI3K Pathway transcript clustering. **Figure S5.** Additional t-SNE profiles for select tumor types, excluding those shown in Fig. 1, demonstrating Purine Biosynthesis Pathway transcript clust. **Figure S6.** Additional t-SNE profiles for select tumor types, excluding those shown in Fig. 1, demonstrating Pyrimidine Biosynthesis Pathway transcript clustering. **Figure S7.** Additional t-SNE profiles for select tumor types, excluding those shown in Fig. 1, demonstrating TP53 Pathway transcript cluste. **Figure S8.** Additional t-SNE profiles for select tumor types, excluding those shown in Fig. 1, demonstrating TGF-β Pathway transcript clustering. **Figure S9.** Additional t-SNE profiles for select tumor types, excluding those shown in Fig. 1, demonstrating Hippo Pathway transcript clustering. **Figure S10.** Additional t-SNE profiles for select tumor types, excluding those shown in Fig. 1, demonstrating Myc Pathway transcript clustering. **Figure S11.** Additional t-SNE profiles for select tumor types, excluding those shown in Fig. 1, demonstrating TCA Cycle transcript clustering. **Figure S12.** Additional t-SNE profiles for select tumor types, excluding those shown in Fig. 1, demonstrating Pentose Phosphate Pathway transcript clustering. **Figure S13.** Additional Kaplan-Meier survival curves for patients with distinct groups of Cell Cycle Pathway t-SNE clusters, excluding those shown in Fig. 2. **Figure S14.** Additional Kaplan-Meier survival curves for patients with distinct groups of Wnt Pathway t-SNE clusters, excluding those shown in Fig. 2. **Figure S15.** Additional Kaplan-Meier survival curves for patients with distinct groups of Notch Pathway t-SNE clusters, excluding those shown in Fig. 2. **Figure S16.** Additional Kaplan-Meier survival curves for patients with distinct groups of PI3K Pathway t-SNE clusters, excluding those shown in Fig. 2. **Figure S17.** Additional Kaplan-Meier survival curves for patients with distinct groups of Purine Biosynthesis Pathway t-SNE clusters, excluding those shown in Fig. 2. **Figure S18.** Additional Kaplan-Meier survival curves for patients with distinct groups of Pyrimidine Biosynthesis Pathway t-SNE clusters, excluding those shown in Fig. 2. **Figure S19.** Additional Kaplan-Meier survival curves for patients with distinct groups of TP53 Pathway t-SNE clusters, excluding those shown in Fig. 2. **Figure S20.** Additional Kaplan-Meier survival curves for patients with distinct groups of TGF-β Pathway t-SNE clusters, excluding those shown in Fig. 2. **Figure S21.** Additional Kaplan-Meier survival curves for patients with distinct groups of Hippo Pathway t-SNE clusters, excluding those shown in Fig. 2. **Figure S22.** Additional Kaplan-Meier survival curves for patients with distinct groups of Myc Pathway t-SNE clusters, excluding those shown in Fig. 2. **Figure S23.** Additional Kaplan-Meier survival curves for patients with distinct groups of TCA Cycle Pathway t-SNE clusters, excluding those shown in Fig. 2. **Figure S24.** Additional Kaplan-Meier survival curves for patients with distinct groups of Pentose Phosphate Pathway t-SNE clusters, excluding those shown in Fig. 2. **Figure S25.** Additional Random Forest Classifiers showing the individual transcripts in the Cell Cycle Pathway that were most deterministic of t-SNE profiles for each relevant tumor type. **Figure S26.** Additional Random Forest Classifiers showing the individual transcripts in the Wnt Pathway that were most deterministic of t-SNE profiles for each relevant tumor type. **Figure S27.** Additional Random Forest Classifiers showing the individual transcripts in the Notch Pathway that were most deterministic of t-SNE profiles for each relevant tumor type. **Figure S28.** Additional Random Forest Classifiers showing the individual transcripts in the PI3K Pathway that were most deterministic of t-SNE profiles for each relevant tumor type. **Figure S29.** Additional Random Forest Classifiers showing the individual transcripts in the Purine Biosynthesis Pathway that were most deterministic of t-SNE profiles for each relevant tumor type. **Figure S30.** Additional Random Forest Classifiers showing the individual transcripts in the Pyrimidine Biosynthesis Pathway that were most deterministic of t-SNE profiles for each relevant tumor type. **Figure S31.** Additional Random Forest Classifiers showing the individual transcripts in the TP53 Pathway that were most deterministic of t-SNE profiles for each relevant tumor type. **Figure S32.** Additional Random Forest Classifiers showing the individual transcripts in the TGF-β Pathway that were most deterministic of t-SNE profiles for each of 11 relevant tumor types, not including those shown in Fig. 4. **Figure S33.** Additional Random Forest Classifiers showing the individual transcripts in the Hippo Pathway that were most deterministic of t-SNE profiles for each relevant tumor type. **Figure S34.** Additional Random Forest Classifiers showing the individual transcripts in the Myc Pathway that were most deterministic of t-SNE profiles for each relevant tumor type. **Figure S35.** Additional Random Forest Classifiers showing the individual transcripts in the TCA Pathway that were most deterministic of t-SNE profiles for each relevant tumor type. **Figure S36.** Additional Random Forest Classifiers showing the individual transcripts in the Pentose Phosphate Pathway that were most deterministic of t-SNE profiles for each relevant tumor type. **Figure S37.** Additional predictive power of sequential t-SNE analyses. (A). The survival curve shown in Fig. 2 of sarcoma patients based on t-SNE clusters from the Purine Biosynthesis Pathway. (B). Cluster 1 patients from A were further analyzed based on whether they could be categorized as Cluster 1 or Cluster 2 when analyzed for TGF-β Pathway transcripts. (C). Cluster 2 patients from A were similarly categorized as in B. (D). Cluster 3 patients from A were similarly categorized as in B. (4). Cluster 4 patients from A were similarly categorized as in B. **Figure S38.** Additional predictive power of sequential t-SNE analyses. (A). The survival curves of head and neck cancer patients based on t-SNE clusters from the Myc Pathway taken from Fig. 2 in the Supplementary Appendix. (B). Cluster 1 patients from A were further analyzed based on whether they could be categorized as Cluster 1, Cluster 2, or cluster 3 when analyzed for cell cycle Pathway transcripts. (C-E). Clusters 2–4 patients from A were similarly categorized as in B. Figs. **Figure S39.** Whole transcriptome analysis refines the predictive power of Pyrimidine Pathway t-SNE profiling in renal clear cell carcinoma (KIRC). (A). Hierarchical clustering of all KIRCs based on whole transcriptome profiling. Each tumor’s t-SNE cluster is indicated and is derived from Fig. 2. (B). Kaplan-Meier survival curves of each of the Dendro groups from A. (C). All t-SNE Cluster 1 tumors with favorable survival (Fig. 2) were further categorized based on their Dendro Groupings. It can be seen that these tumors were associated with a worse overall survival if they fell into the Dendro 1 group. Similarly, t-SNE cluster 2 tumors with overall unfavorable survival could be further sub-classified according to their Dendro group. **Figure S40.** Whole transcriptome analysis refines the predictive power of Myc Pathway t-SNE profiling in sarcoma (SARC). (A). Hierarchical clustering of all sarcoma patients identified 4 distinct Dendro Groups (1–4). The two t-SNE Clusters into which these tumors fell are indicated at the bottom of the heat map. Note that the Dendro 1 Group is particularly weighted with t-SNE Cluster 2 tumors having favorable survival. To a somewhat lesser extent, the Dendro 4 Group was more heavily populated by t-SNE Cluster 1 tumors with unfavorable survival. (B). Survival for each of the Dendro Groups in (A) showing that Dendro Groups 1 and 2 were associated with relatively favorable survival whereas Dendro group 4 was associated with unfavorable survival. (C). t-SNE Cluster 1 unfavorable survival tumors could be further subdivided based on their Dendro Group identities. (D). t-SNE Cluster 2 favorable survival tumors could also be subdivided further based on there whole transcriptome profile. **Figure S41.** Whole transcriptome analysis refines the predictive power of TCA Cycle Pathway in bladder urothelial cancer (BLCA). (A). Hierarchical cluster of all tumors identified 4 Dendro Groups. Note that Dendro Groups 1 and 2 are over-represented by t-SNE Cluster 2 TCA Pathway tumors with an intermediate survival whereas Dendro Group 4 is over-represented by t-SNE Cluster 3 tumors with a relatively favorable survival (Figs. S11 and S23 in the Supplementary Appendix). (B). Kaplan-Meier survival curves of each of the 4 Dendro Groups in (A). (C-E). Kaplan-Meier survival curves of each of the 3 t-SNE Groups. Note that the t-SNE Cluster 1 could not be further subdivided by further hierarchical clustering whereas both t-SNE Clusters 2 and 3 could. (DOC 23261 kb)

## Data Availability

This work is in part based on data generated from TCGA Research Network (http://cancergenome.nih.gov). Clinical annotation files and gene expression data were downloaded from UCSC Xenabrowser (https://xenabrowser.net) [16]. The software used to excecute tSNE is available for free at (https://projector.tensorflow.org/) [17].

## Abbreviations

FAO: Fatty acid oxidation
FPKM-UQ: Fragments per kilobase-million upper quartile
RPT: Ribosomal protein transcript
TCGA: The Cancer Genome ATLAS
TNBC: Triple negative breast cancer
t-SNE: t-distributed stochastic nearest neighbor embedding
