# Supplementary material for: Expression patterns of small numbers of transcripts from functionally-related pathways predict survival in multiple cancers
Source: BMC Cancer. 2019 Jul 12;19:686. doi: 10.1186/s12885-019-5851-6 (PMC6626418; doi:10.1186/s12885-019-5851-6)
Supplement: Supplementary file 1 — Table S1. Component Transcripts and NCBI Gene ID Numbers Used for t-SNE Profiling in Each of Twelve Cancer-Related Pathways. Note that, although there are a total of 221 transcripts listed, 9 of those in the Purine and Pyrimidine Biosynthesis Pathways (depicted in bold) are common. Thus, a total of 212 unique transcripts were used for generating t-SNE profiles. Table S2. Abbreviations for and Number of Cancers in Each of the TCGA Groups and Those for Which Survival Data is Unavailable. Table S3. t-SNE clustering parameters. For “Diagonal” covariance matrices only the diagonal entries were non-zero, and the principle axes of the fitted Gaussians were parallel to the X,Y, and Z axes. For “Full” covariance matrices any entry could be nonzero and the principle axes of the fitted Gaussians could be oriented in any direction. Shared Covariance: in cases where this is “TRUE” each fitted Gaussian had the same covariance matrix. Where this was “FALSE” every fitted Gaussian had a unique covariance matrix. Perturb Input: where this is “TRUE” the t-SNE data were randomly perturbed by a maximum of 5% of the radius of the sphere enclosing them prior to clustering. Perturb Output: Where this is “TRUE”, the t-SNE scatter-plots displayed in the figures have the afore-mentioned perturbation applied. Figure S1. Additional t-SNE profiles for select tumor types, excluding those shown in Fig. 1, demonstrating Cell Cycle Pathway transcript clustering. Figure S2. Additional t-SNE profiles for select tumor types, excluding those shown in Fig. 1, demonstrating Wnt Pathway transcript clustering. Figure S3. Additional t-SNE profiles for select tumor types, excluding those shown in Fig. 1, demonstrating Notch Pathway transcript clustering. Figure S4. Additional t-SNE profiles for select tumor types, excluding those shown in Fig. 1, demonstrating PI3K Pathway transcript clustering. Figure S5. Additional t-SNE profiles for select tumor types, excluding those shown in Fig. 1, demonstrating Purine [file 12885_2019_5851_MOESM1_ESM.doc]

**Supplementary Information for:**

**Expression Patterns of Small Numbers of Transcripts from Functionally-Related Pathways Predict Survival in Multiple Cancers**

**Table of Contents**

**pp. 29-38. Supplementary Tables S1-S3**

**pp. 39-80. Supplementary Figures S1-S41**

| Table S1. | Transcripts Used for t-SNE Profiling | |
| --- | --- | --- |
| **Pathway/Gene Family** | **Gene Symbol** | **Entrez ID** |
| **Cell Cycle (15 members)** | *RB1* | 5925 |
|  | *CDKN2C* | 1031 |
|  | *CDKN2B* | 1030 |
|  | *CDKN2A* | 1029 |
|  | *CDKN1B* | 1027 |
|  | *CDKN1A* | 1026 |
|  | *E2F3* | 1871 |
|  | *E2F1* | 1869 |
|  | *CDK6* | 1021 |
|  | *CDK4* | 1019 |
|  | *CDK2* | 1017 |
|  | *CCNE1* | 898 |
|  | *CCND3* | 896 |
|  | *CCND2* | 894 |
|  | *CCND1* | 595 |
| **Wnt/β-Catenin (25 members)** | *ZNRF3* | 84133 |
|  | *WIF1* | 11197 |
|  | *TLE4* | 7091 |
|  | *TLE3* | 7090 |
|  | *TLE2* | 7089 |
|  | *TLE1* | 7088 |
|  | *TCF7L2* | 6934 |
|  | *TCF7L1* | 83439 |
|  | *TCF7* | 6932 |
|  | *SFRP5* | 6425 |
|  | *SFRP4* | 6424 |
|  | *SFRP2* | 6423 |
|  | *SFRP1* | 6422 |
|  | *RNF43* | 54894 |
|  | *LRP5* | 4041 |
|  | *GSK3B* | 2932 |
|  | *DKK4* | 27121 |
|  | *DKK3* | 27122 |
|  | *DKK2* | 27123 |
|  | *DKK1* | 22943 |
|  | *CTNNB1* | 1499 |
|  | *AXIN2* | 8313 |
|  | *AXIN1* | 8312 |
|  | *APC* | 324 |
|  | *AMER1* | 139285 |
| **Notch (30 members)** | *ARRDC1* | 92714 |
|  | *CNTN6* | 27255 |
|  | *CREBBP* | 1387 |
|  | *EP300* | 2033 |
|  | *HES1* | 3280 |
|  | *HES2* | 54626 |
|  | *HES3* | 390992 |
|  | *HES4* | 57801 |
|  | *HES5* | 388585 |
|  | *HEY1* | 23462 |
|  | *HEY2* | 23493 |
|  | *HEYL* | 26508 |
|  | *KAT2B* | 8850 |
|  | *KDM5A* | 5927 |
|  | *NOTCH1* | 4851 |
|  | *NOTCH2* | 4853 |
|  | *NOTCH3* | 4854 |
|  | *NOTCH4* | 4855 |
|  | *NOV* | 4856 |
|  | *PSEN2* | 5664 |
|  | *SPEN* | 23013 |
|  | *FBXW7* | 55294 |
|  | *THBS2* | 7058 |
|  | *CUL1* | 8454 |
|  | *NCOR1* | 9611 |
|  | *NCOR2* | 9612 |
|  | *HDAC1* | 3065 |
|  | *JAG2* | 3714 |
|  | *MAML3* | 55534 |
|  | *DNER* | 92737 |
| **PI3K (18 members)** | *MTOR* | 2475 |
|  | *RICTOR* | 253260 |
|  | *RPTOR* | 57521 |
|  | *RHEB* | 6009 |
|  | *TSC2* | 7249 |
|  | *TSC1* | 7248 |
|  | *PPP2R1A* | 5518 |
|  | *AKT3* | 10000 |
|  | *AKT2* | 208 |
|  | *AKT1* | 207 |
|  | *STK11* | 6794 |
|  | *INPP4B* | 8821 |
|  | *PIK3R3* | 8503 |
|  | *PIK3R2* | 5296 |
|  | *PIK3R1* | 5295 |
|  | *PTEN* | 5728 |
|  | *PIK3CB* | 5291 |
|  | *PIK3CA* | 5290 |
| **Purine Biosynthesis (25 members)** | *PPAT* | 5471 |
|  | *GART* | 2618 |
|  | *PFAS* | 5198 |
|  | *PAICS* | 10606 |
|  | *ADSL* | 158 |
|  | *ATIC* | 471 |
|  | *ADSSL1* | 122622 |
|  | *ADSS* | 159 |
|  | *AK1* | 203 |
|  | *AK2* | 204 |
|  | *AK3* | 50808 |
|  | *AK4* | 205 |
|  | *AK5* | 26289 |
|  | *AK7* | 122481 |
|  | ***RRM1*** | **6240** |
|  | ***RRM2*** | **6241** |
|  | *GMPS* | 8833 |
|  | *GUK1* | 2987 |
|  | ***NME1*** | **4830** |
|  | ***NME2*** | **4831** |
|  | ***NME3*** | **4832** |
|  | ***NME4*** | **4833** |
|  | ***NME5*** | **8382** |
|  | ***NME6*** | **10201** |
|  | ***NME7*** | **29922** |
| **Pyrimidine Biosynthesis (23 members)** | *CAD* | 790 |
|  | *DHODH* | 1723 |
|  | *UMPS* | 7372 |
|  | *CMPK1* | 51727 |
|  | *CMPK2* | 129607 |
|  | ***NME1*** | **4830** |
|  | ***NME2*** | **4831** |
|  | ***NME3*** | **4832** |
|  | ***NME4*** | **4833** |
|  | ***NME5*** | **8382** |
|  | ***NME6*** | **10201** |
|  | ***NME7*** | **29922** |
|  | *CTPS1* | 1503 |
|  | *CTPS2* | 56474 |
|  | ***RRM1*** | **6240** |
|  | ***RRM2*** | **6241** |
|  | *DUT* | 1854 |
|  | *ENPP3* | 5169 |
|  | *ENPP1* | 5167 |
|  | *ITPA* | 3704 |
|  | *TYMS* | 7298 |
|  | *DTYMK* | 1841 |
|  | *NTPCR* | 84284 |
| **TP53 (6 members)** | *CHEK2* | 11200 |
|  | *ATM* | 472 |
|  | *TP53* | 7157 |
|  | *RPS6KA3* | 6197 |
|  | *MDM4* | 4194 |
|  | *MDM2* | 4193 |
| **TGF-β (7 members)** | *TGFBR1* | 7046 |
|  | *TGFBR2* | 7048 |
|  | *ACVR2A* | 92 |
|  | *ACVR1B* | 91 |
|  | *SMAD2* | 4087 |
|  | *SMAD3* | 4088 |
|  | *SMAD4* | 4089 |
| **Hippo (27 members)** | *YAP1* | 10413 |
|  | *WWTR1* | 25937 |
|  | *TEAD2* | 8463 |
|  | *STK4* | 6789 |
|  | *STK3* | 6788 |
|  | *SAV1* | 60485 |
|  | *LATS1* | 9113 |
|  | *LATS2* | 26524 |
|  | *MOB1A* | 55233 |
|  | *MOB1B* | 92597 |
|  | *PTPN14* | 5784 |
|  | *NF2* | 4771 |
|  | *WWC1* | 23286 |
|  | *TAOK1* | 57551 |
|  | *TAOK2* | 9344 |
|  | *TAOK3* | 51347 |
|  | *CRB1* | 23418 |
|  | *CRB2* | 286204 |
|  | *CRB3* | 92359 |
|  | *FAT1* | 2195 |
|  | *FAT2* | 2196 |
|  | *FAT3* | 120114 |
|  | *FAT4* | 79633 |
|  | *DCHS1* | 8642 |
|  | *DCHS2* | 54798 |
|  | *CSNK1E* | 1454 |
|  | *CSNK1D* | 1453 |
| **Myc (13 members)** | *MYC* | 4609 |
|  | *MXI1* | 4601 |
|  | *MYCL* | 4610 |
|  | *MYCN* | 4613 |
|  | *MAX* | 4149 |
|  | *MXD1* | 4084 |
|  | *MXD3* | 83463 |
|  | *MXD4* | 10608 |
|  | *MLX* | 6945 |
|  | *MLXIPL* | 51085 |
|  | *MLXIP* | 22877 |
|  | *MNT* | 4335 |
|  | *MGA* | 23269 |
| **TCA Cycle (21 members)** | *OGDH* | 4967 |
|  | *OGDHL* | 55753 |
|  | *CS* | 1431 |
|  | *ACO1* | 48 |
|  | *ACO2* | 50 |
|  | *IDH1* | 3417 |
|  | *IDH2* | 3418 |
|  | *IDH3A* | 3419 |
|  | *IDH3B* | 3420 |
|  | *IDH3G* | 3421 |
|  | *SUCLA2* | 8803 |
|  | *SUCLG1* | 8802 |
|  | *SUCLG2* | 8801 |
|  | *SDHA* | 6389 |
|  | *SDHB* | 6390 |
|  | *SDHC* | 6391 |
|  | *SDHD* | 6392 |
|  | *FH* | 2271 |
|  | *MDH1* | 4190 |
|  | *MDH1B* | 130752 |
|  | MDH2 | 4191 |
| **Pentose Phosphate Pathway (11 members)** | *H6PD* | 9563 |
|  | *PGLS* | 25796 |
|  | *G6PD* | 2539 |
|  | *RPIA* | 22934 |
|  | *PGD* | 5226 |
|  | *RPE* | 6120 |
|  | *RPEL1* | 729020 |
|  | *TALDO1* | 6888 |
|  | *TKT* | 7086 |
|  | *TKTL1* | 8277 |
|  | *TKTL2* | 84076 |

**Table S1.** Component Transcripts and NCBI Gene ID Numbers Used for t-SNE Profiling in Each of Twelve Cancer-Related Pathways. Note that, although there are a total of 221 transcripts listed, 9 of those in the Purine and Pyrimidine Biosynthesis Pathways (depicted in bold) are common. Thus, a total of 212 unique transcripts were used for generating t-SNE profiles.

| Table S2. | Cancer Abbreviations and Number of Tumors |  |
| --- | --- | --- |
| **Abbreviation** | **Cancer Type** | **Number of Tumors** |
| ACC | Adrenocortical Carcinoma | 79 |
| BLCA | Bladder Urothelial Carcinoma | 411 |
| BRIC | Breast Invasive Carcinoma | 1097 |
| CESC | Cervical/Endocervical Squamous Cell Carcinoma | 304 |
| CHOL | Cholangiocarcinoma | 36 |
| COAD | Colon adenocarcinoma | 469 |
| DLBC | Diffuse Large B-Cell Lymphoma | 48 |
| ESCA | Esophageal Carcinoma | 161 |
| GBM | Glioblastoma Multiforme | 155 |
| HNSC | Head & Neck Squamous Cell Carcinoma | 500 |
| KICH | Kidney Chromophobe | 65 |
| KIRC | Kidney Renal Clear Cell Carcinoma | 534 |
| KIRP | Kidney Renal Papillary Cell Carcinoma | 288 |
| LAML | Acute Myeloid Leukemia (bone marrow) | 119 |
| LGG | Brain Lower-grade glioma | 511 |
| LIHC | Liver Hepatocellular Carcinoma | 371 |
| LUAD | Lung Adenocarcinoma | 524 |
| LUSC | Lung Squamous Cell Carcinoma | 501 |
| MESO | Mesothelioma | 86 |
| OV | Ovarian Serous Cystadenocarcinoma | 374 |
| PAAD | Pancreatic Adenocarcinoma | 177 |
| PCPG | Pheomocrhomocytoma and Paraganglioma | 178 |
| PRAD | Prostate Adenocarcinoma | 498 |
| READ | Rectum Adenocarcinoma | 166 |
| SARC | Sarcoma | 259 |
| SKCM | Skin Cutaneous Melanoma (metastatic) | 367 |
| STAD | Stomach Adenocarcinoma | 375 |
| TGCT | Testicular Germ Cell Tumors | 150 |
| THCA | Thyroid Carcinoma | 502 |
| THYM | Thymoma | 119 |
| UCEC | Uterine Corpus Endometrial Carcinoma | 547 |
| UCS | Uterine Carcinsarcoma | 56 |
| UVM | Uveal Melanoma | 80 |
| WT | High Risk Wilms' Tumor | 120 |

**Table S2.** Abbreviations for and Number of Cancers in Each of the TCGA Groups and Those for Which Survival Data is Unavailable.

| Table S3. | t-SNE Parameters | | |  |  |  |  |
| --- | --- | --- | --- | --- | --- | --- | --- |
| **Pathway** | **Cancer** | **Perplexity** | **Learning Rate** | **Covariance Type** | **Shared Covariance** | **Perturb Input** | **Perturb Output** |
| Cell Cycle | CESC | 12 | 10 | Full | TRUE | TRUE | FALSE |
| Cell Cycle | HNSC | 13 | 100 | Full | TRUE | FALSE | FALSE |
| Cell Cycle | KICH | 5 | 1 | Diagonal | FALSE | TRUE | FALSE |
| Cell Cycle | KIRC | 17 | 100 | Full | TRUE | FALSE | FALSE |
| Cell Cycle | KIRP | 8 | 10 | Diagonal | FALSE | TRUE | FALSE |
| Cell Cycle | LAML | 7 | 1 | Diagonal | FALSE | TRUE | FALSE |
| Cell Cycle | LIHC | 13 | 100 | Diagonal | FALSE | FALSE | TRUE |
| Cell Cycle | MESO | 5 | 1 | Diagonal | FALSE | TRUE | FALSE |
| Cell Cycle | OV | 12 | 10 | Full | TRUE | TRUE | FALSE |
| Cell Cycle | PAAD | 5 | 100 | Diagonal | FALSE | TRUE | FALSE |
| Cell Cycle | SKCM | 9 | 100 | Diagonal | FALSE | TRUE | FALSE |
| Cell Cycle | THYM | 9 | 100 | Full | TRUE | FALSE | FALSE |
| Cell Cycle | UCEC | 13 | 10 | Full | TRUE | FALSE | FALSE |
| Cell Cycle | UVM | 8 | 10 | Full | TRUE | FALSE | FALSE |
| Wnt | BLCA | 16 | 10 | Full | TRUE | FALSE | TRUE |
| Wnt | BRCA | 21 | 10 | Diagonal | FALSE | TRUE | FALSE |
| Wnt | HRWT | 10 | 10 | Diagonal | FALSE | FALSE | TRUE |
| Wnt | KIRC | 12 | 10 | Full | TRUE | TRUE | TRUE |
| Wnt | KIRP | 13 | 100 | Full | TRUE | FALSE | FALSE |
| Wnt | LGG | 16 | 10 | Full | TRUE | FALSE | TRUE |
| Wnt | LUAD | 15 | 10 | Full | TRUE | FALSE | FALSE |
| Wnt | SKCM | 16 | 10 | Diagonal | FALSE | FALSE | TRUE |
| Wnt | THCA | 18 | 100 | Diagonal | FALSE | TRUE | FALSE |
| Wnt | THYM | 10 | 10 | Diagonal | FALSE | TRUE | FALSE |
| Wnt | UCEC | 22 | 10 | Diagonal | FALSE | TRUE | FALSE |
| Wnt | UVM | 12 | 10 | Diagonal | FALSE | FALSE | TRUE |
| Notch | BRCA | 17 | 100 | Diagonal | FALSE | TRUE | TRUE |
| Notch | HRWT | 8 | 1 | Diagonal | FALSE | TRUE | FALSE |
| Notch | KIRC | 10 | 100 | Diagonal | FALSE | TRUE | FALSE |
| Notch | LGG | 18 | 10 | Diagonal | FALSE | TRUE | FALSE |
| Notch | MESO | 5 | 10 | Diagonal | FALSE | FALSE | TRUE |
| Notch | SKCM | 11 | 10 | Full | TRUE | FALSE | FALSE |
| Notch | UVM | 8 | 10 | Full | TRUE | FALSE | FALSE |
| PI 3-Kinase | KIRC | 11 | 100 | Diagonal | FALSE | TRUE | FALSE |
| PI 3-Kinase | LGG | 12 | 10 | Diagonal | FALSE | TRUE | FALSE |
| PI 3-Kinase | LIHC | 11 | 10 | Diagonal | TRUE | FALSE | FALSE |
| Purine | BRCA | 18 | 100 | Full | TRUE | FALSE | FALSE |
| Purine | CESC | 9 | 100 | Diagonal | TRUE | FALSE | FALSE |
| Purine | HRWT | 5 | 10 | Full | FALSE | FALSE | FALSE |
| Purine | KIRC | 11 | 100 | Full | FALSE | TRUE | FALSE |
| Purine | LAML | 5 | 10 | Full | TRUE | FALSE | FALSE |
| Purine | LIHC | 7 | 10 | Full | TRUE | TRUE | TRUE |
| Purine | LUAD | 9 | 100 | Full | TRUE | FALSE | FALSE |
| Purine | MESO | 11 | 1 | Full | TRUE | FALSE | FALSE |
| Purine | PAAD | 9 | 0.1 | Full | TRUE | FALSE | FALSE |
| Purine | SARC | 7 | 100 | Diagonal | FALSE | TRUE | FALSE |
| Purine | UCEC | 10 | 10 | Full | FALSE | FALSE | FALSE |
| Purine | UVM | 8 | 10 | Diagonal | FALSE | TRUE | FALSE |
| Pyrimidine | ACC | 7 | 10 | Full | FALSE | FALSE | FALSE |
| Pyrimidine | BRCA | 17 | 100 | Diagonal | TRUE | FALSE | FALSE |
| Pyrimidine | KICH | 5 | 10 | Full | TRUE | FALSE | FALSE |
| Pyrimidine | KIRC | 30 | 1 | Full | TRUE | FALSE | FALSE |
| Pyrimidine | LGG | 11 | 10 | Diagonal | FALSE | TRUE | FALSE |
| Pyrimidine | LIHC | 10 | 10 | Diagonal | FALSE | TRUE | FALSE |
| Pyrimidine | OV | 10 | 10 | Full | TRUE | FALSE | FALSE |
| Pyrimidine | THYM | 11 | 10 | Diagonal | FALSE | TRUE | FALSE |
| Pyrimidine | UCEC | 10 | 10 | Diagonal | FALSE | TRUE | FALSE |
| TP53 | ACC | 8 | 10 | Diagonal | FALSE | TRUE | FALSE |
| TP53 | GBM | 14 | 10 | Diagonal | FALSE | TRUE | FALSE |
| TP53 | KIRC | 12 | 10 | Full | FALSE | FALSE | FALSE |
| TP53 | LGG | 12 | 10 | Diagonal | FALSE | TRUE | FALSE |
| TGF-β | SARC | 8 | 100 | Diagonal | FALSE | TRUE | FALSE |
| TP53 | STAD | 15 | 10 | Diagonal | FALSE | TRUE | FALSE |
| TP53 | UCS | 11 | 10 | Full | TRUE | FALSE | FALSE |
| TGF-β | ACC | 7 | 10 | Full | TRUE | FALSE | FALSE |
| TGF-β | ESCA | 9 | 1 | Diagonal | FALSE | FALSE | FALSE |
| TGF-β | HRWT | 9 | 100 | Diagonal | FALSE | TRUE | FALSE |
| TGF-β | KIRC | 12 | 10 | Full | TRUE | FALSE | FALSE |
| TGF-β | LGG | 11 | 10 | Diagonal | TRUE | FALSE | FALSE |
| TGF-β | LIHC | 9 | 10 | Diagonal | FALSE | FALSE | FALSE |
| TGF-β | LUAD | 13 | 10 | Full | TRUE | FALSE | FALSE |
| Hippo | CHOL | 5 | 10 | Diagonal | FALSE | TRUE | FALSE |
| Hippo | COAD | 10 | 10 | Diagonal | FALSE | TRUE | FALSE |
| Hippo | LAML | 7 | 1 | Full | FALSE | FALSE | FALSE |
| Hippo | LGG | 9 | 100 | Diagonal | FALSE | TRUE | TRUE |
| Hippo | MESO | 5 | 1 | Diagonal | TRUE | FALSE | FALSE |
| Hippo | SKCM | 8 | 100 | Full | TRUE | FALSE | FALSE |
| Hippo | THYM | 5 | 10 | Full | TRUE | FALSE | FALSE |
| Myc | ACC | 5 | 1 | Full | FALSE | TRUE | FALSE |
| Myc | BLCA | 11 | 10 | Diagonal | FALSE | TRUE | FALSE |
| Myc | CHOL | 5 | 1 | Diagonal | FALSE | TRUE | FALSE |
| Myc | HNSC | 9 | 10 | Full | TRUE | FALSE | FALSE |
| Myc | HRWT | 9 | 1 | Diagonal | FALSE | TRUE | FALSE |
| Myc | KIRP | 13 | 10 | Full | TRUE | FALSE | FALSE |
| Myc | LGG | 10 | 1 | Diagonal | FALSE | FALSE | FALSE |
| Myc | LUAD | 9 | 10 | Full | TRUE | FALSE | FALSE |
| Myc | PAAD | 9 | 10 | Full | TRUE | TRUE | TRUE |
| Myc | SARC | 10 | 10 | Diagonal | FALSE | TRUE | FALSE |
| Myc | UCEC | 11 | 100 | Full | TRUE | FALSE | FALSE |
| TCA | BLCA | 12 | 10 | Full | TRUE | FALSE | FALSE |
| TCA | GBM | 6 | 10 | Diagonal | TRUE | FALSE | FALSE |
| TCA | KIRP | 6 | 10 | Full | TRUE | TRUE | FALSE |
| TCA | LAML | 6 | 1 | Full | TRUE | FALSE | FALSE |
| TCA | PRAD | 11 | 10 | Diagonal | FALSE | TRUE | FALSE |
| TCA | READ | 8 | 10 | Diagonal | FALSE | TRUE | FALSE |
| TCA | UCS | 6 | 10 | Full | TRUE | FALSE | FALSE |
| TCA | UVM | 5 | 1 | Diagonal | FALSE | TRUE | FALSE |
| Pentose Phosphate | ACC | 5 | 10 | Diagonal | FALSE | FALSE | FALSE |
| Pentose Phosphate | BRCA | 9 | 100 | Diagonal | FALSE | FALSE | TRUE |
| Pentose Phosphate | ESCA | 7 | 10 | Diagonal | TRUE | FALSE | FALSE |
| Pentose Phosphate | KIRC | 11 | 100 | Diagonal | FALSE | TRUE | FALSE |
| Pentose Phosphate | KIRP | 10 | 100 | Full | TRUE | FALSE | FALSE |
| Pentose Phosphate | LGG | 11 | 100 | Diagonal | FALSE | FALSE | FALSE |
| Pentose Phosphate | LIHC | 10 | 10 | Full | TRUE | FALSE | FALSE |
| Pentose Phosphate | MESO | 7 | 1 | Diagonal | TRUE | TRUE | FALSE |
| Pentose Phosphate | SARC | 9 | 10 | Diagonal | FALSE | FALSE | FALSE |
| Pentose Phosphate | THYM | 8 | 1 | Full | TRUE | FALSE | FALSE |
| Pentose Phosphate | UVM | 5 | 10 | Full | TRUE | FALSE | FALSE |

**Table S3.** t-SNE clustering parameters. For “Diagonal” covariance matrices only the diagonal entries were non-zero, and the principle axes of the fitted Gaussians were parallel to the X,Y, and Z axes. For “Full” covariance matrices any entry could be nonzero and the principle axes of the fitted Gaussians could be oriented in any direction. Shared Covariance: in cases where this is “TRUE” each fitted Gaussian had the same covariance matrix. Where this was “FALSE” every fitted Gaussian had a unique covariance matrix. Perturb Input: where this is “TRUE” the t-SNE data were randomly perturbed by a maximum of 5% of the radius of the sphere enclosing them prior to clustering. Perturb Output: Where this is “TRUE”, the t-SNE scatter-plots displayed in the figures have the afore-mentioned perturbation applied.

**Supplementary Figures**

**
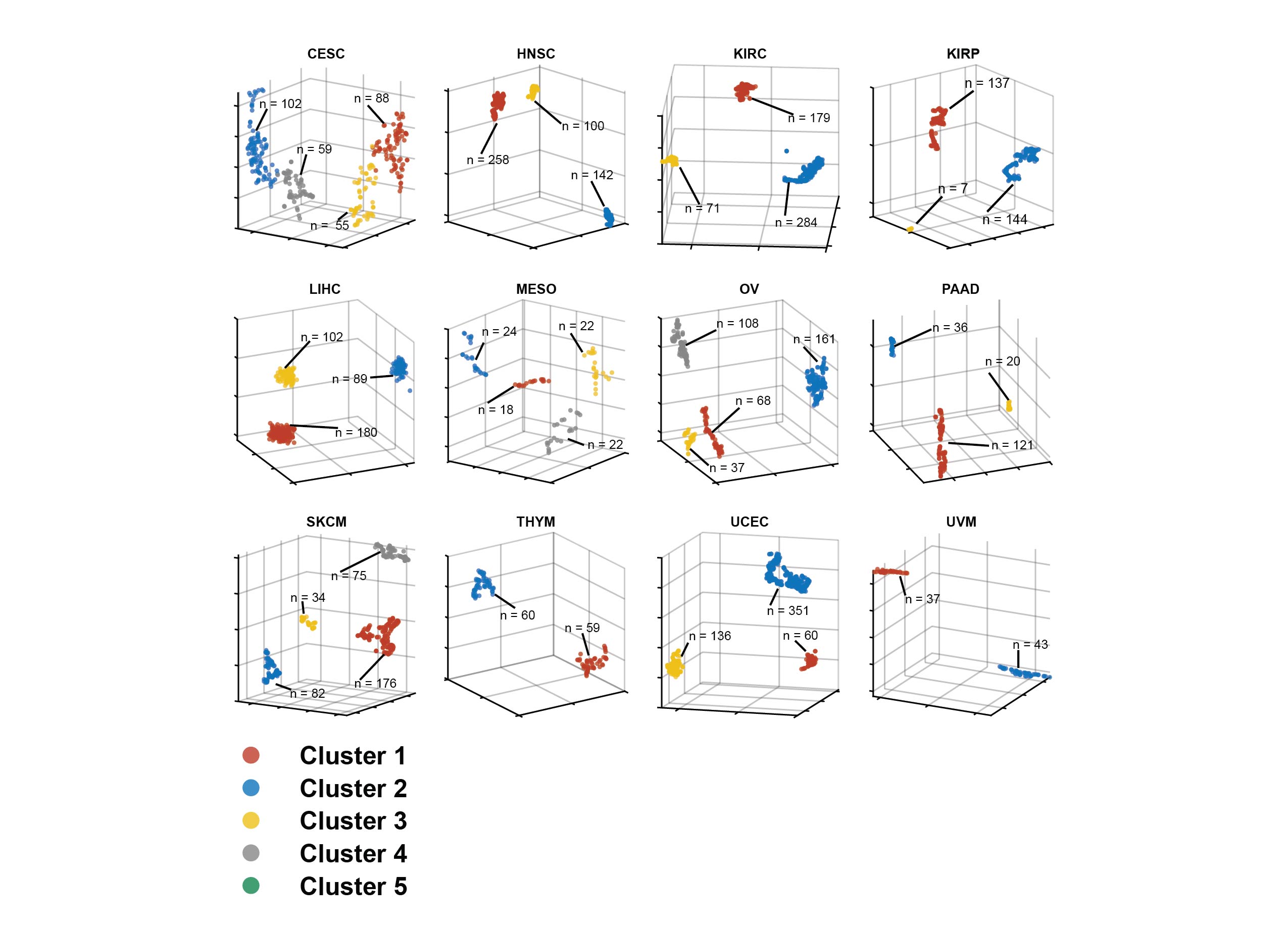
**

**Figure S1.** Additional t-SNE profiles for select tumor types, excluding those shown in Fig. 1, demonstrating Cell Cycle Pathway transcript clustering.

**
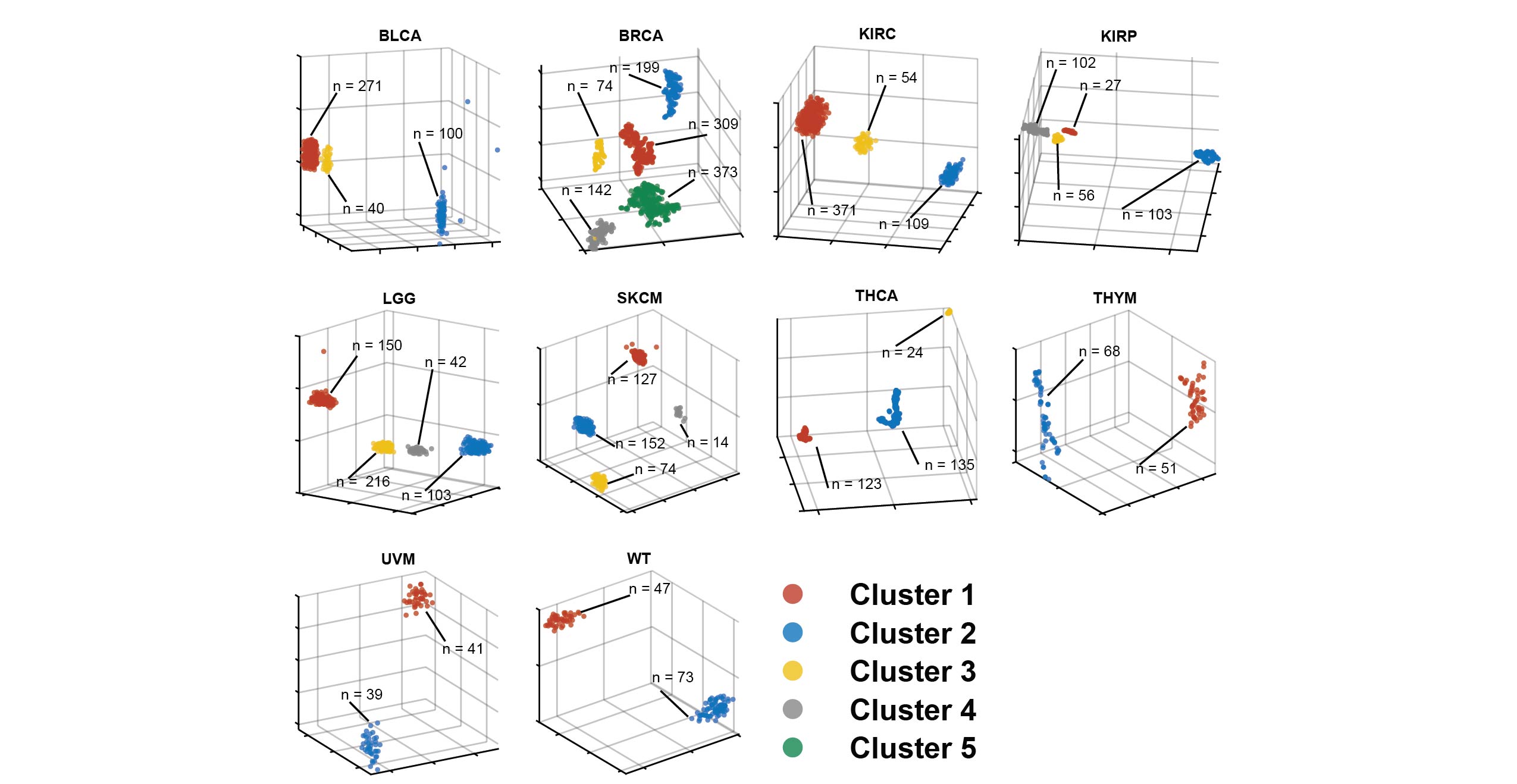
**

**Figure S2.** Additional t-SNE profiles for select tumor types, excluding those shown in Fig. 1, demonstrating Wnt Pathway transcript clustering.

**
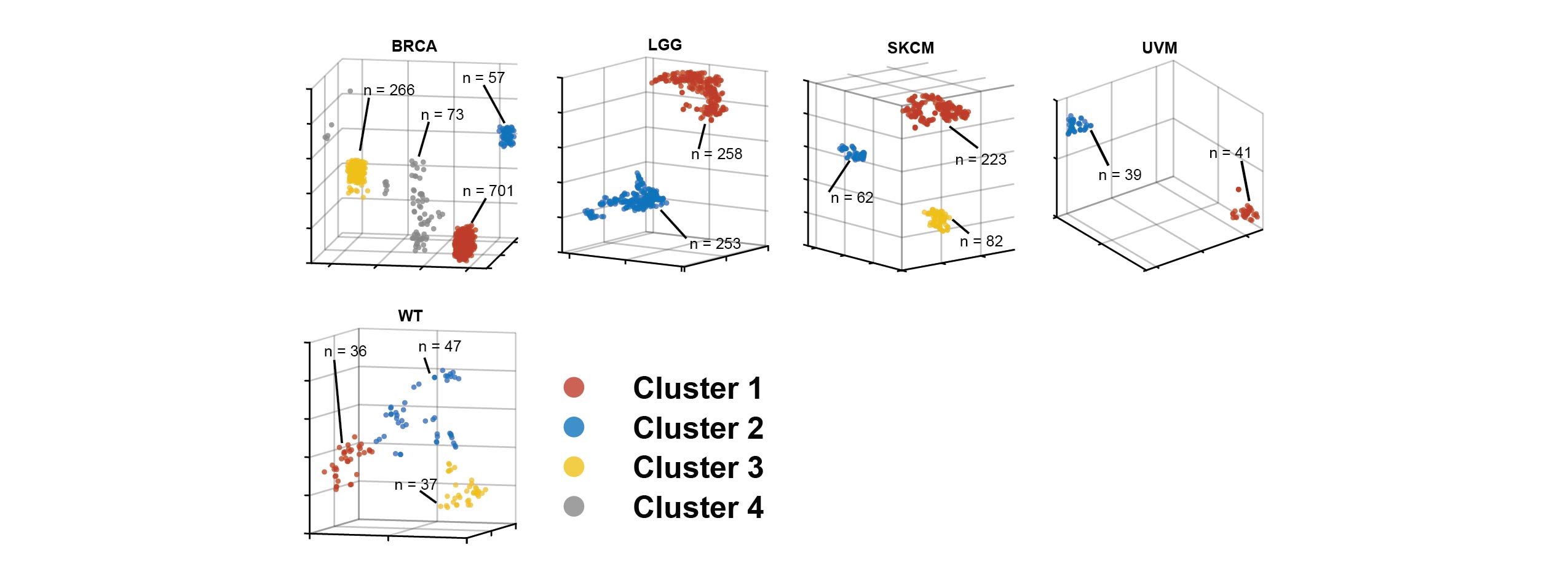
**

**Figure S3.** Additional t-SNE profiles for select tumor types, excluding those shown in Fig. 1, demonstrating Notch Pathway transcript clustering.

**
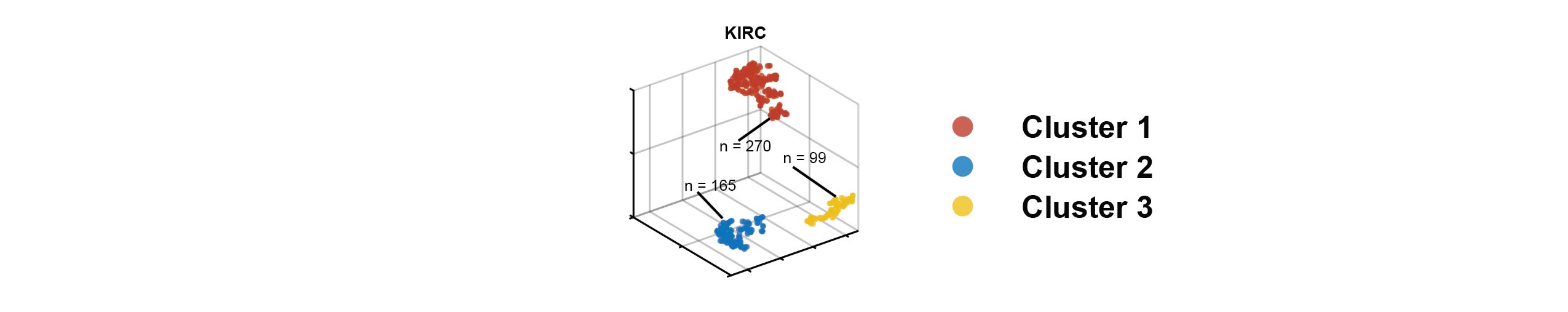
**

**Figure S4.** Additional t-SNE profiles for select tumor types, excluding those shown in Fig. 1, demonstrating PI3K Pathway transcript clustering.


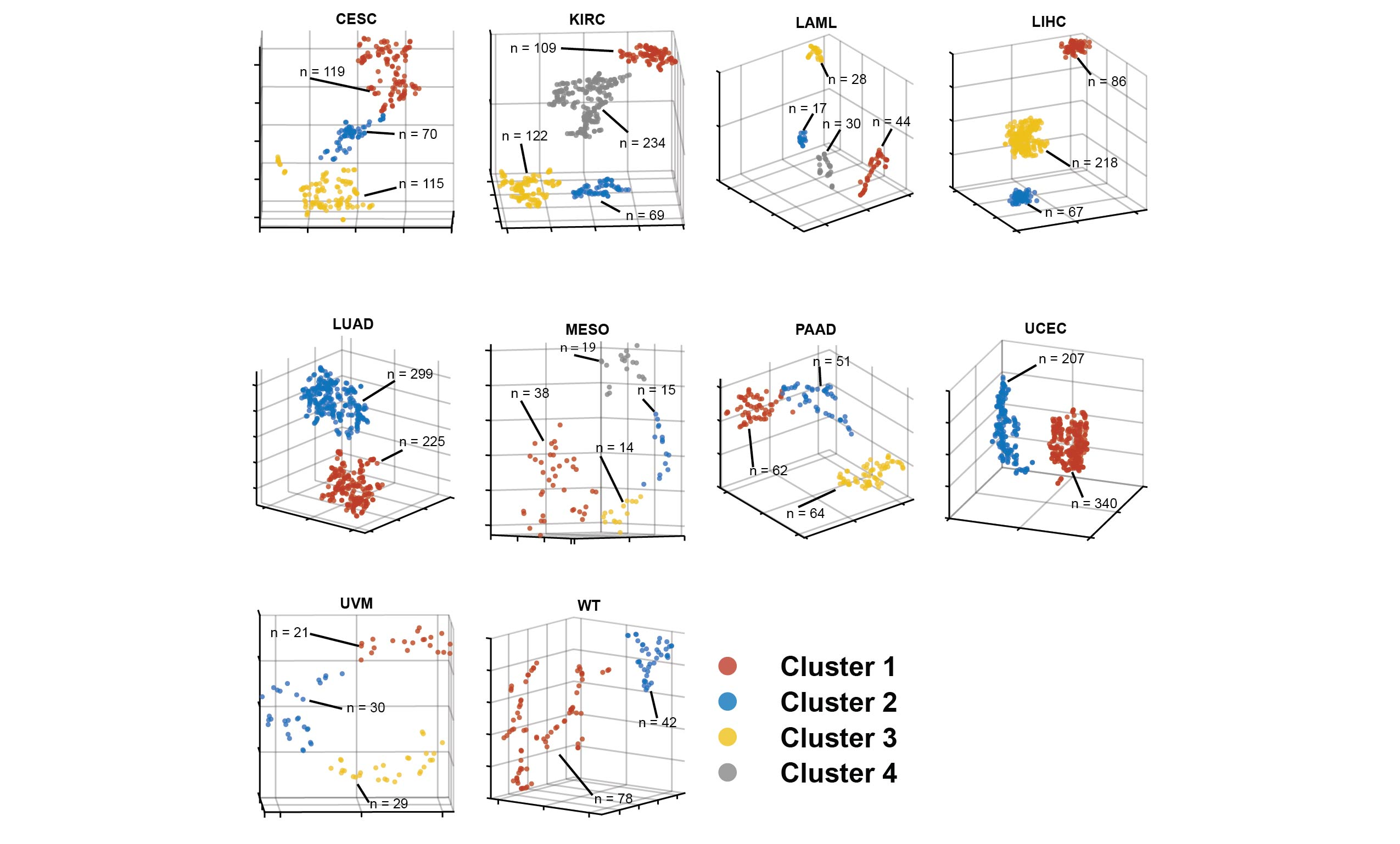


**Figure S5.** Additional t-SNE profiles for select tumor types, excluding those shown in Fig. 1, demonstrating Purine Biosynthesis Pathway transcript clustering.


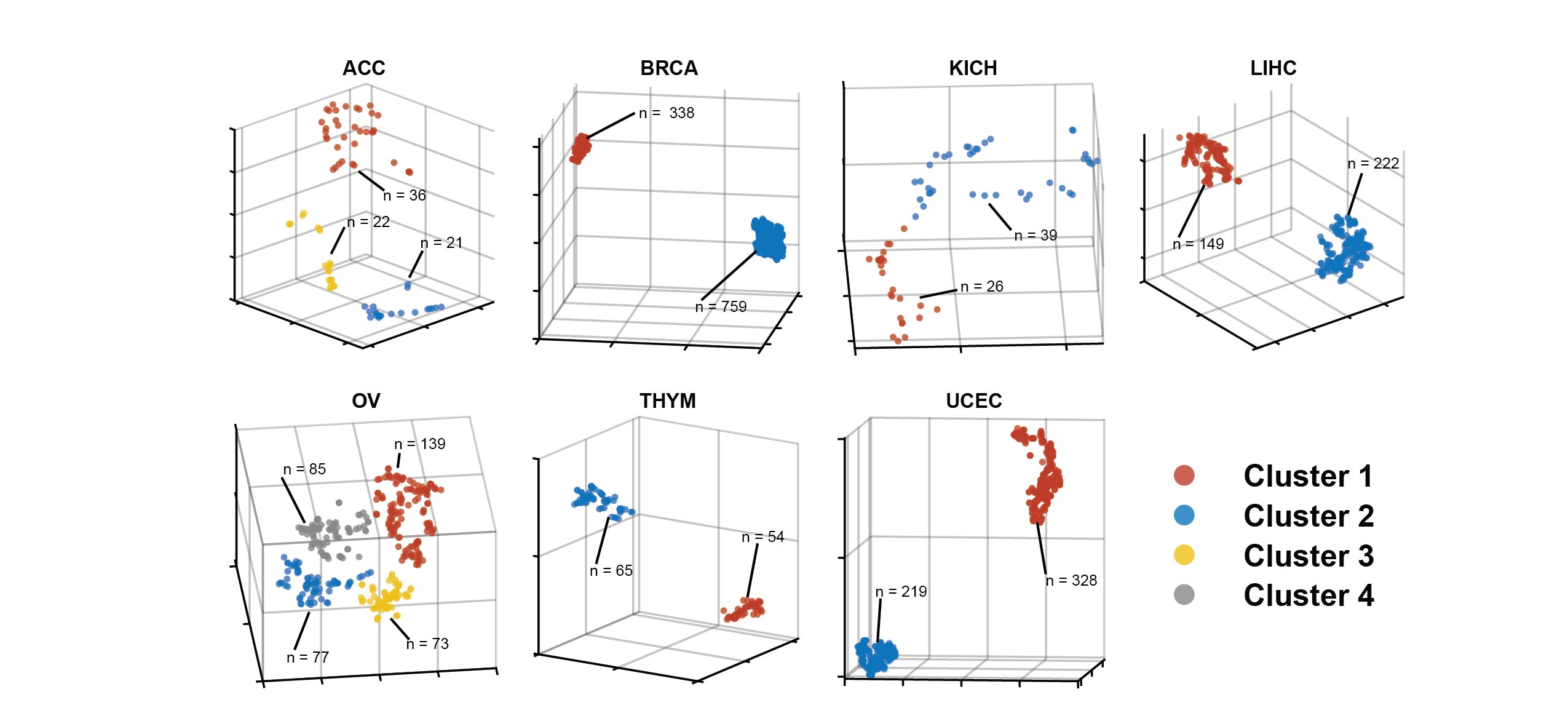


**Figure S6.** Additional t-SNE profiles for select tumor types, excluding those shown in Fig. 1, demonstrating Pyrimidine Biosynthesis Pathway transcript clustering.


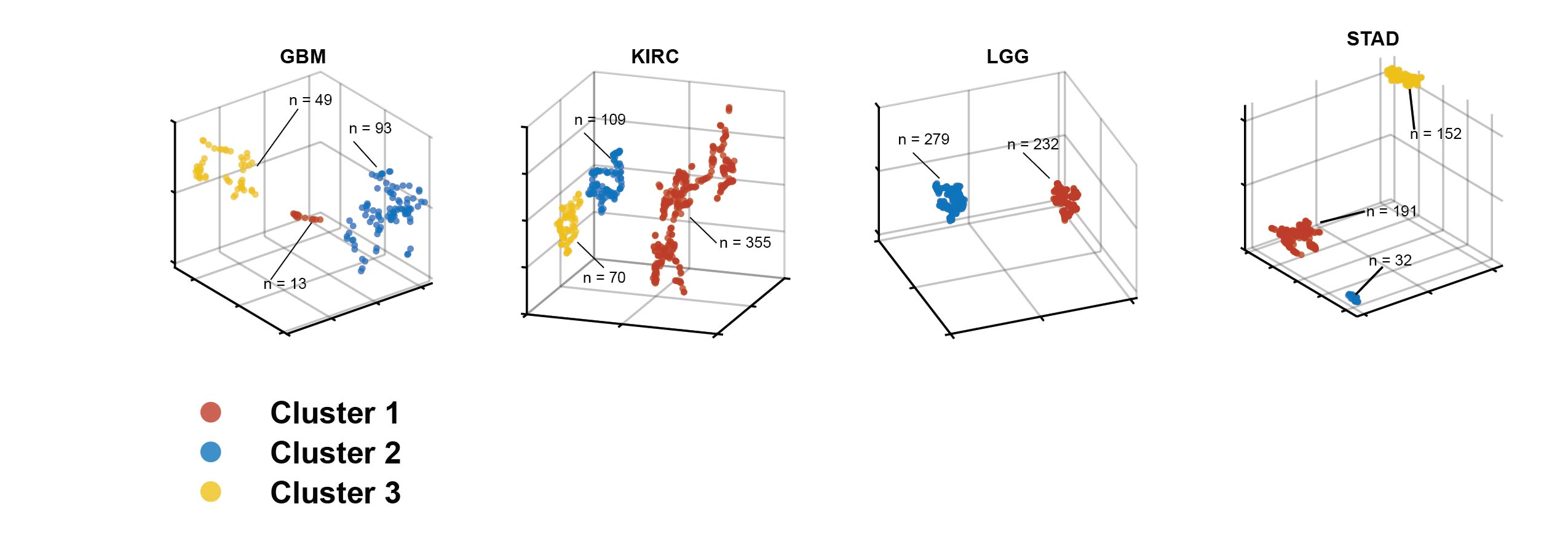


**Figure S7.** Additional t-SNE profiles for select tumor types, excluding those shown in Fig. 1, demonstrating TP53 Pathway transcript clustering.


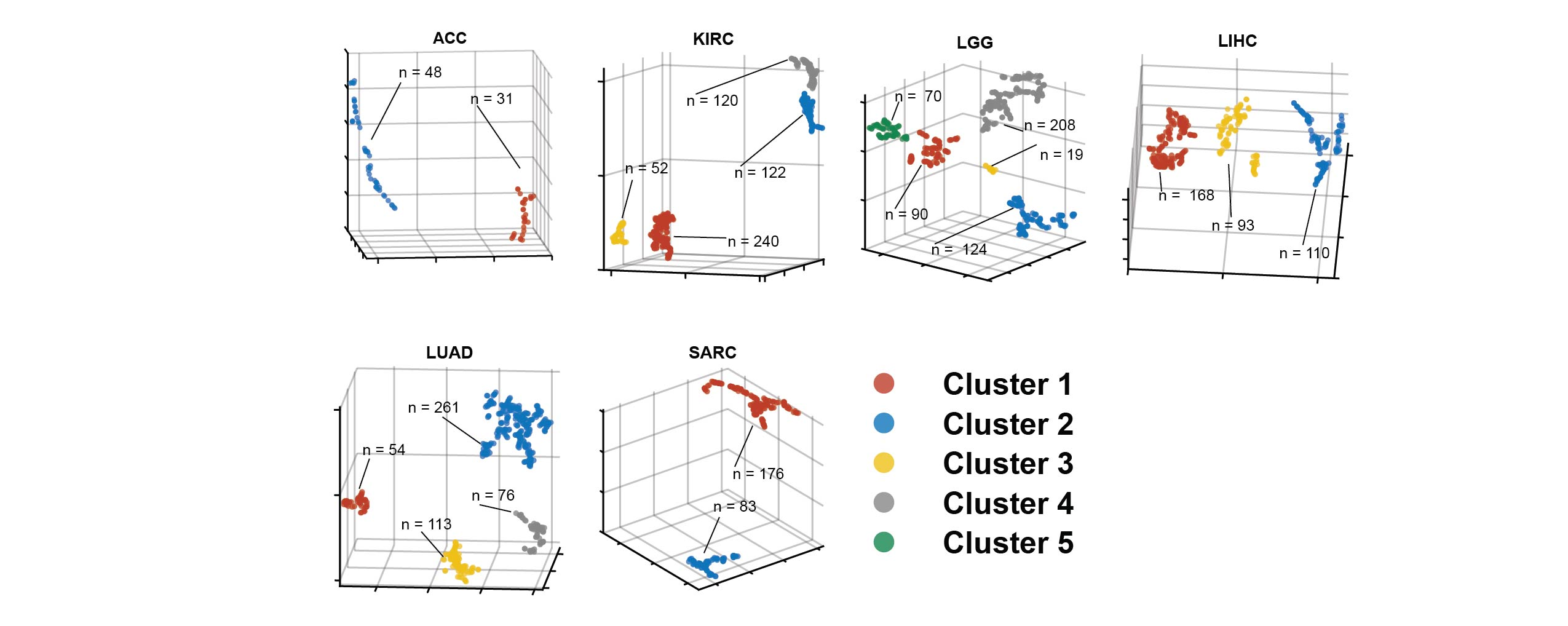


**Figure S8.** Additional t-SNE profiles for select tumor types, excluding those shown in Fig. 1, demonstrating TGF-β Pathway transcript clustering.

**
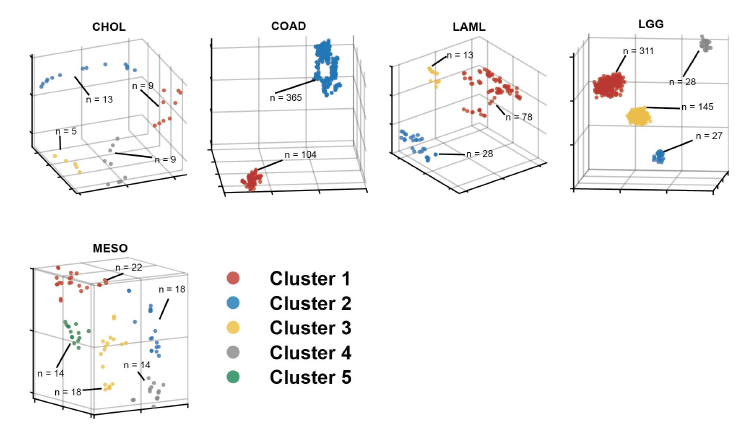
**

**Figure S9.** Additional t-SNE profiles for select tumor types, excluding those shown in Fig. 1, demonstrating Hippo Pathway transcript clustering.

**
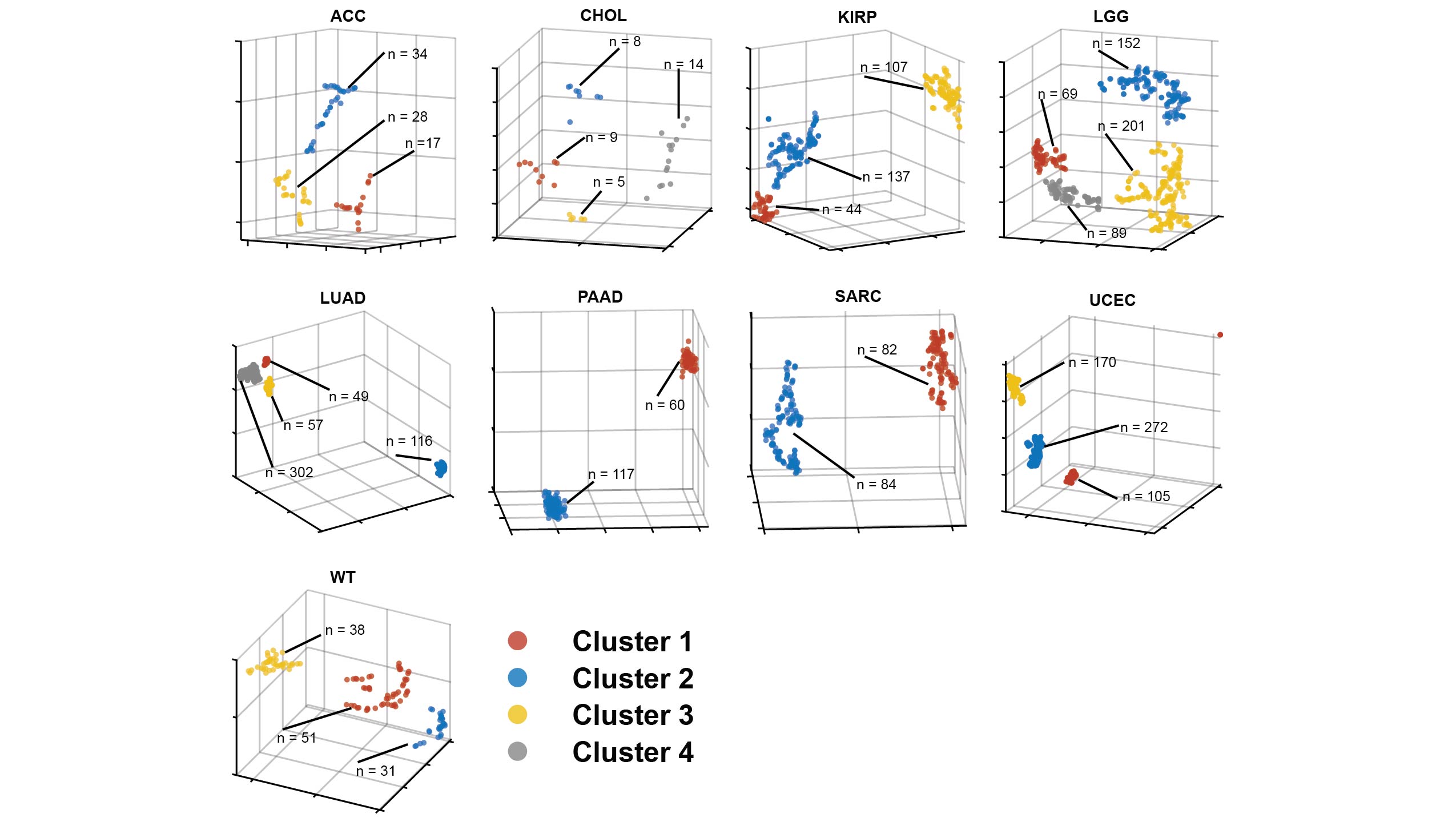
**

**Figure S10.** Additional t-SNE profiles for select tumor types, excluding those shown in Fig. 1, demonstrating Myc Pathway transcript clustering.


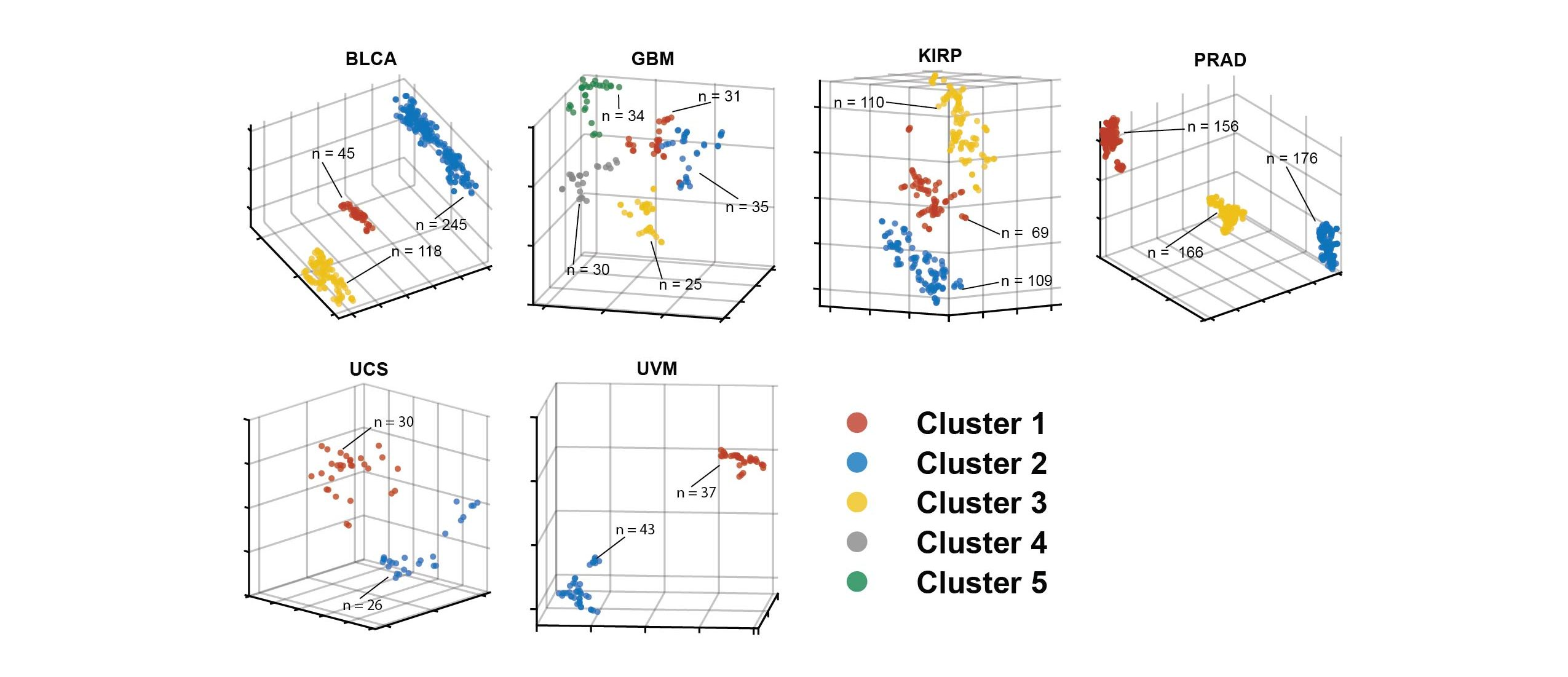


**Figure S11.** Additional t-SNE profiles for select tumor types, excluding those shown in Fig. 1, demonstrating TCA Cycle transcript clustering.


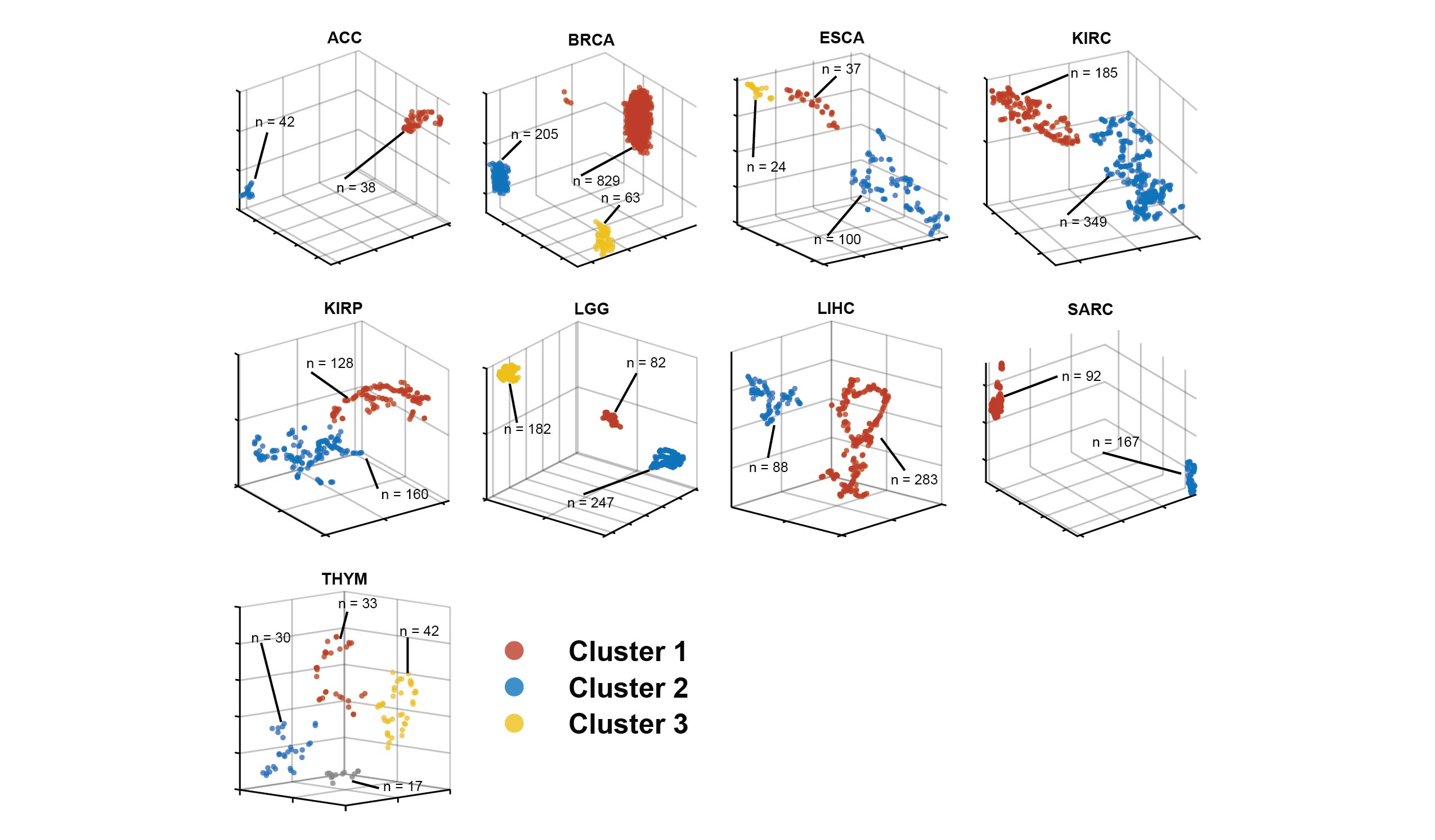


**Figure S12.** Additional t-SNE profiles for select tumor types, excluding those shown in Fig. 1, demonstrating Pentose Phosphate Pathway transcript clustering.

**
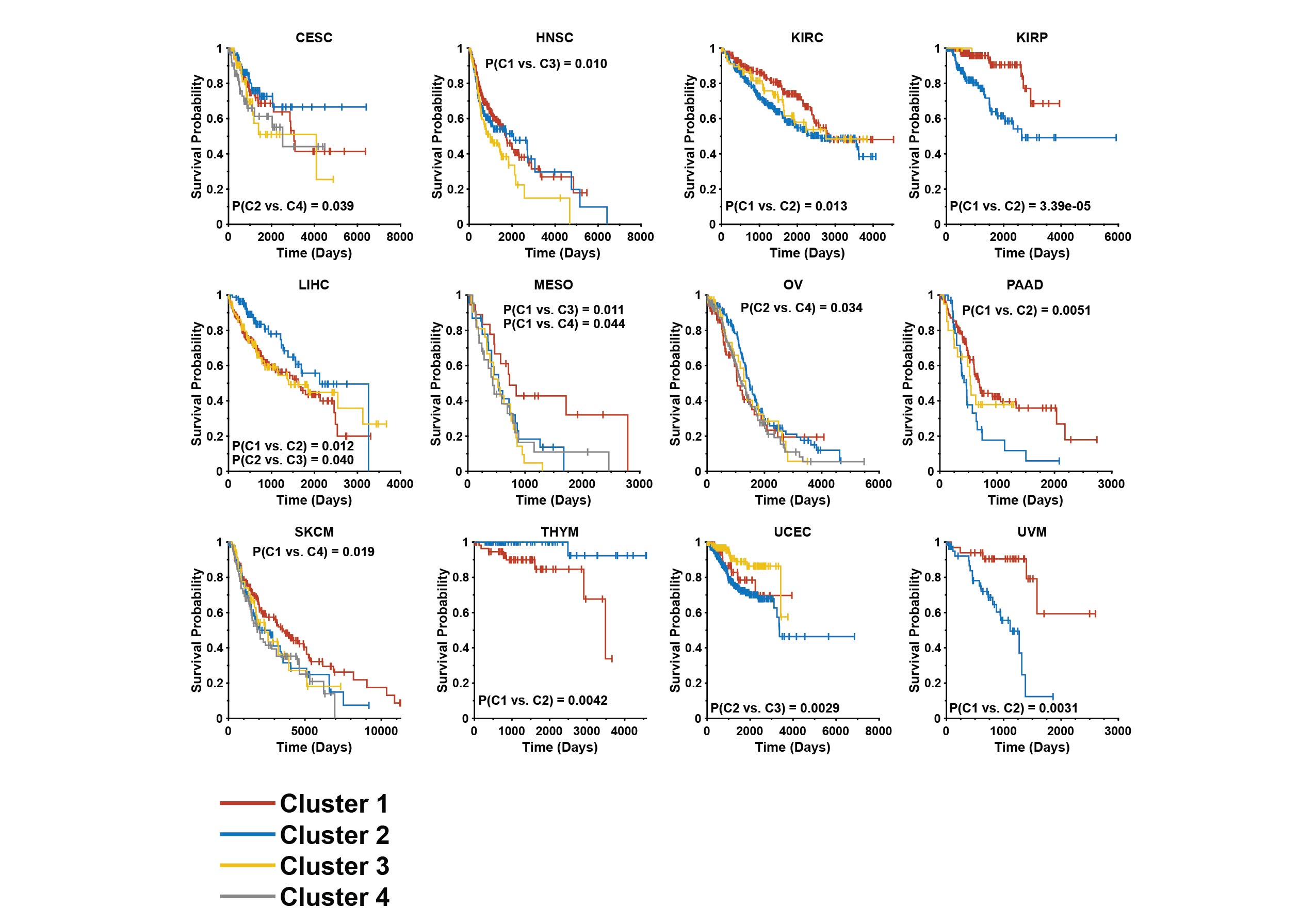
**

**Figure S13.** Additional Kaplan-Meier survival curves for patients with distinct groups of Cell Cycle Pathway t-SNE clusters, excluding those shown in Fig. 2.

**
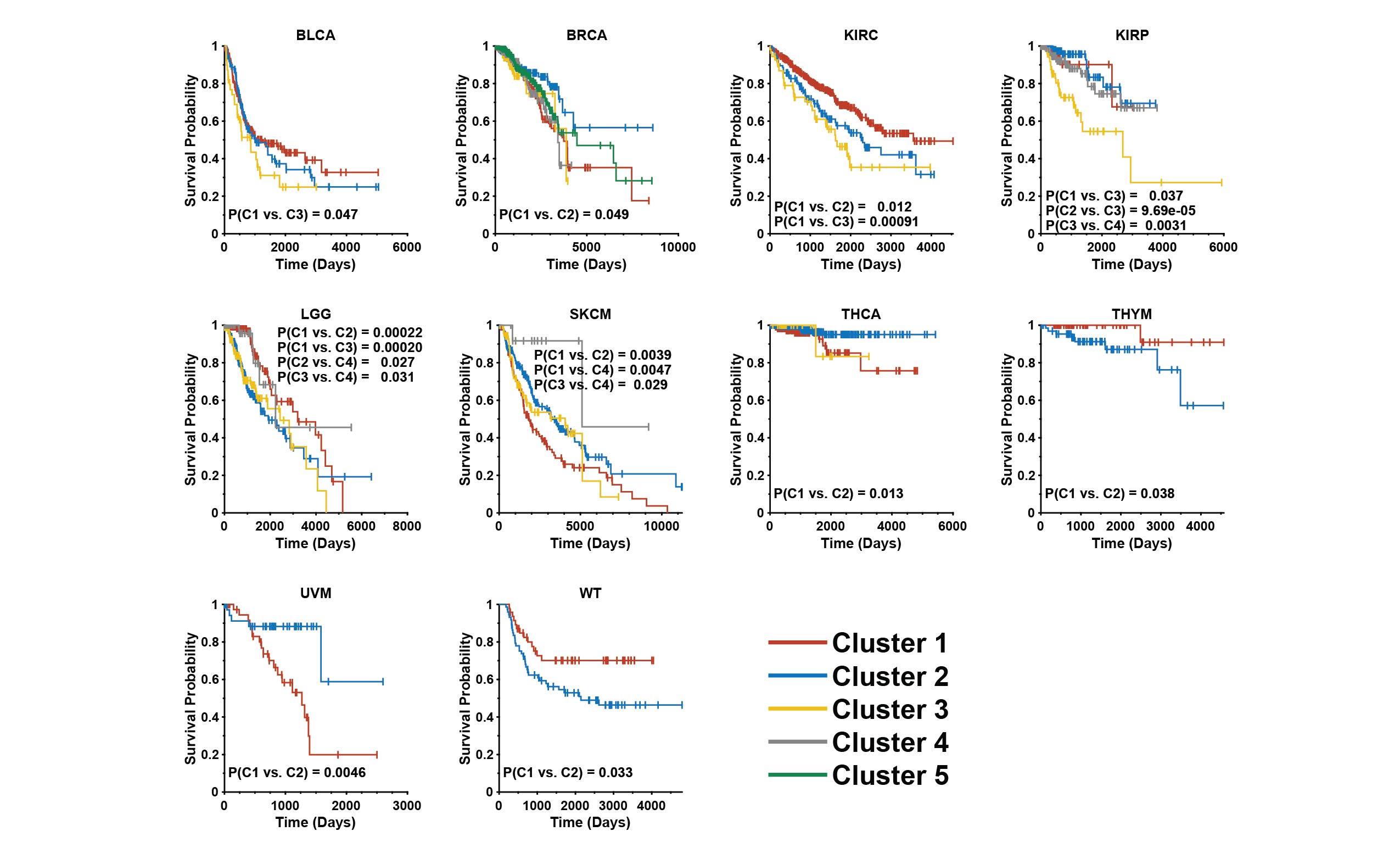
**

**Figure S14.** Additional Kaplan-Meier survival curves for patients with distinct groups of Wnt Pathway t-SNE clusters, excluding those shown in Fig. 2.

**
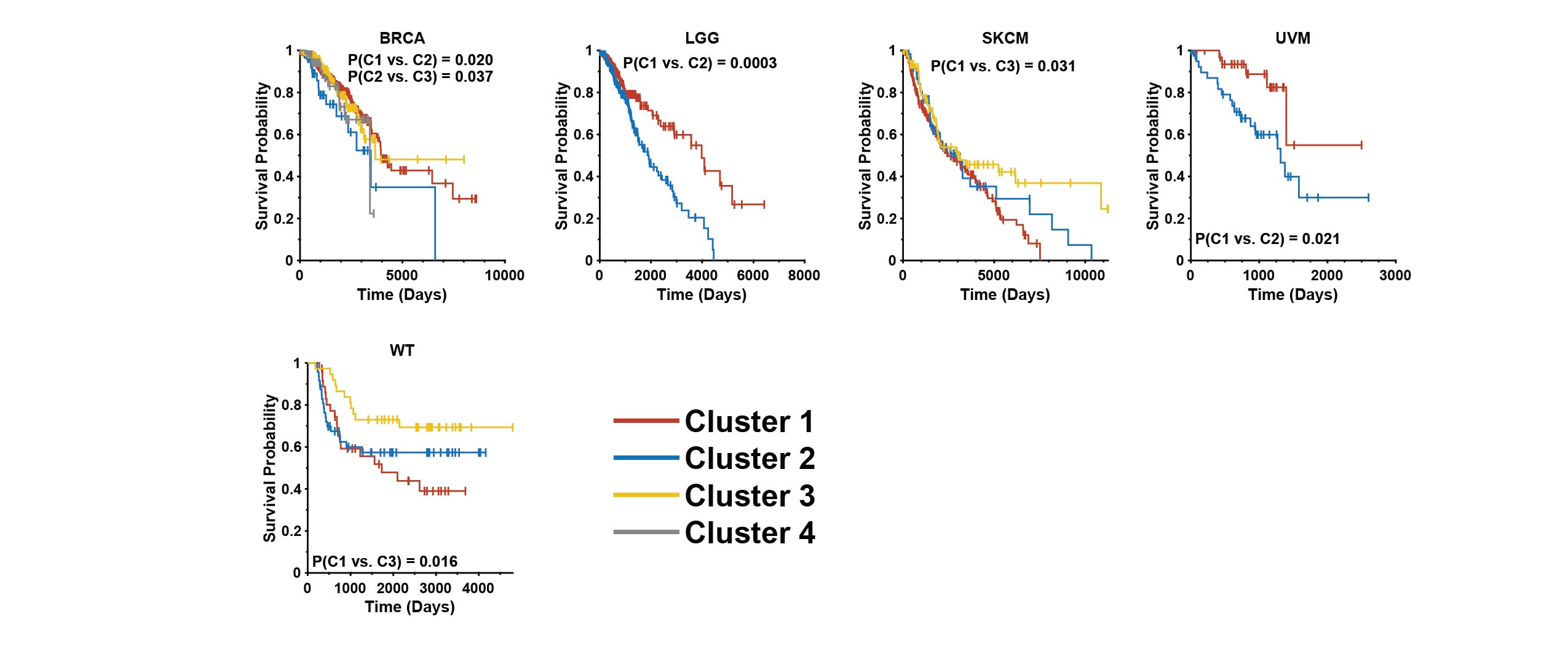
**

**Figure S15.** Additional Kaplan-Meier survival curves for patients with distinct groups of Notch Pathway t-SNE clusters, excluding those shown in Fig. 2.

**
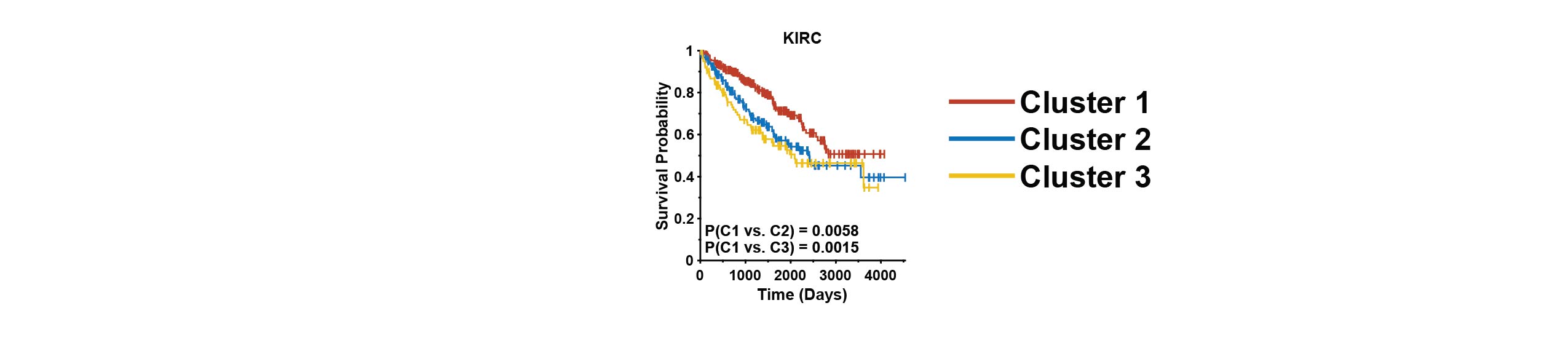
**

**Figure S16.** Additional Kaplan-Meier survival curves for patients with distinct groups of PI3K Pathway t-SNE clusters, excluding those shown in Fig. 2.

**
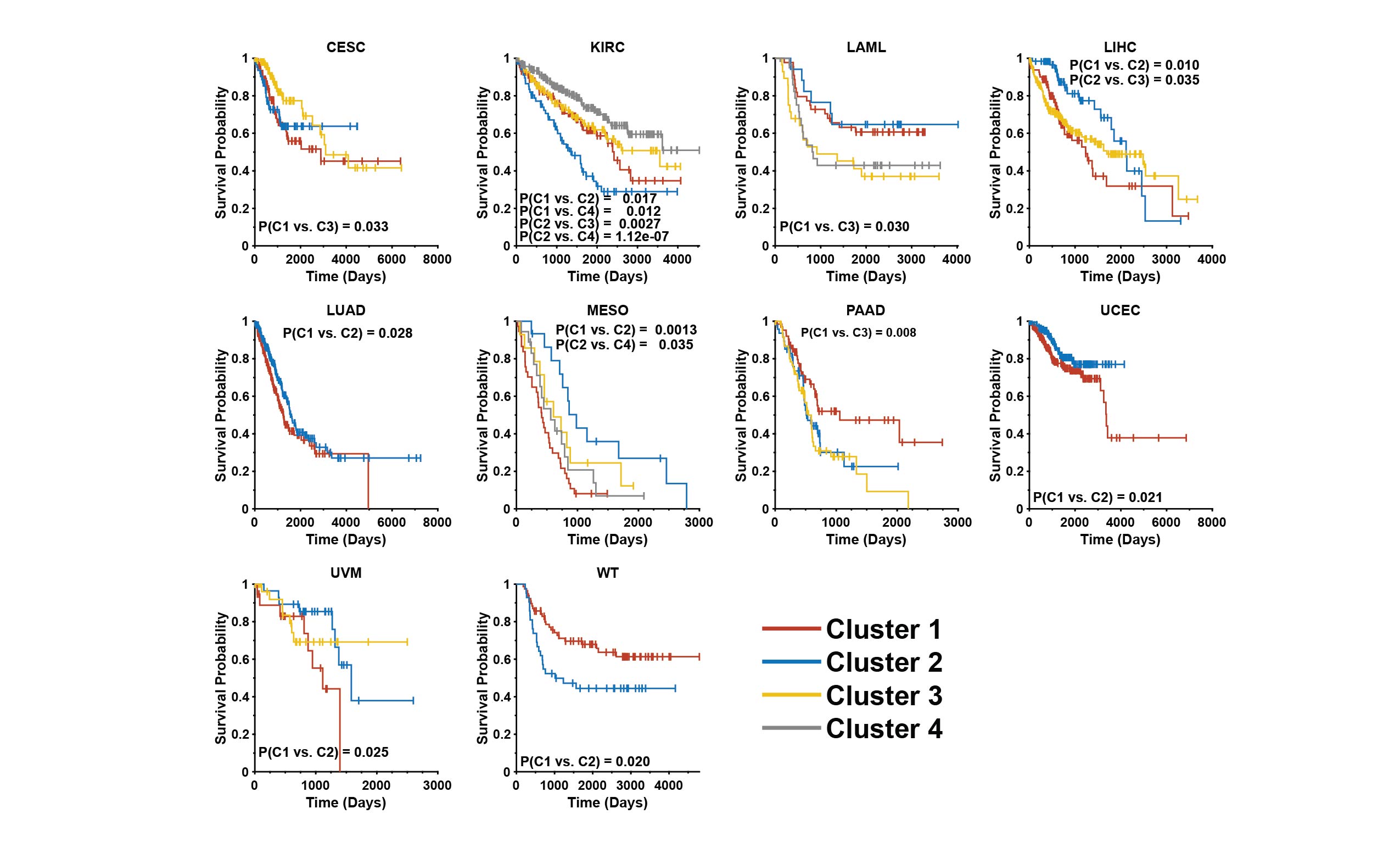
**

**Figure S17.** Additional Kaplan-Meier survival curves for patients with distinct groups of Purine Biosynthesis Pathway t-SNE clusters, excluding those shown in Fig. 2.

**
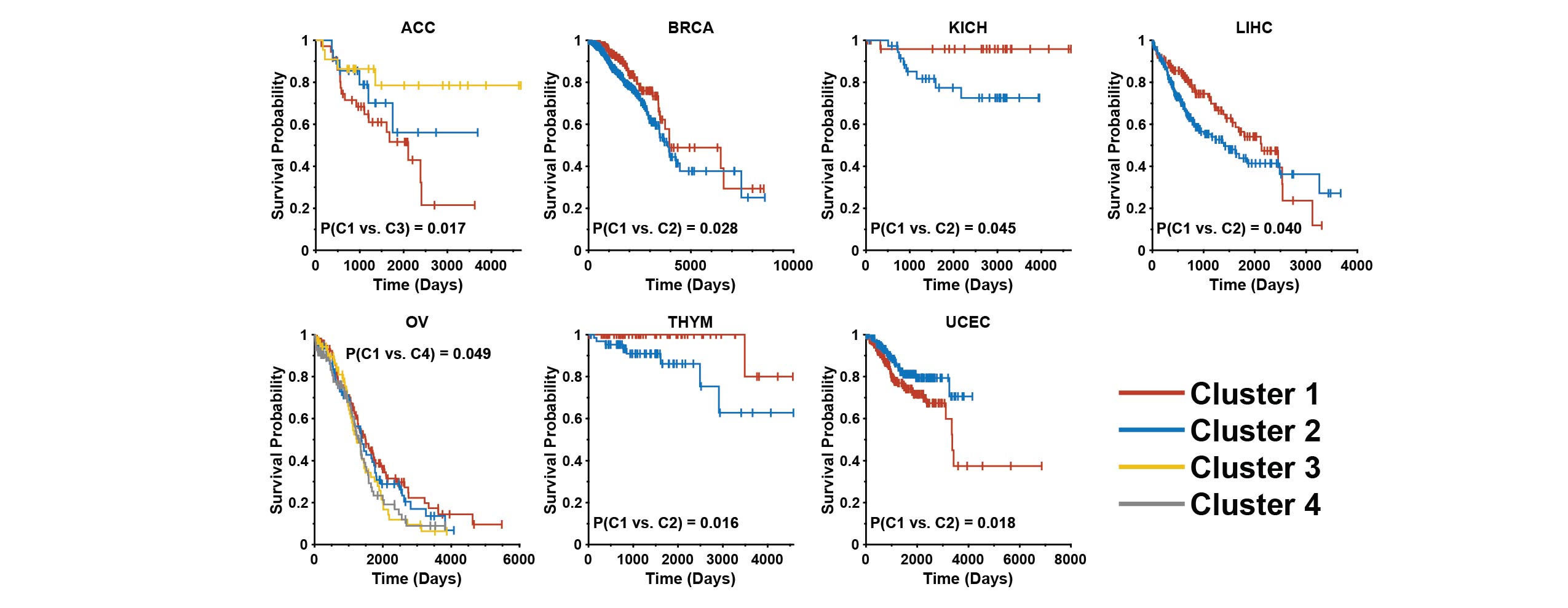
**

**Figure S18.** Additional Kaplan-Meier survival curves for patients with distinct groups of Pyrimidine Biosynthesis Pathway t-SNE clusters, excluding those shown in Fig. 2.


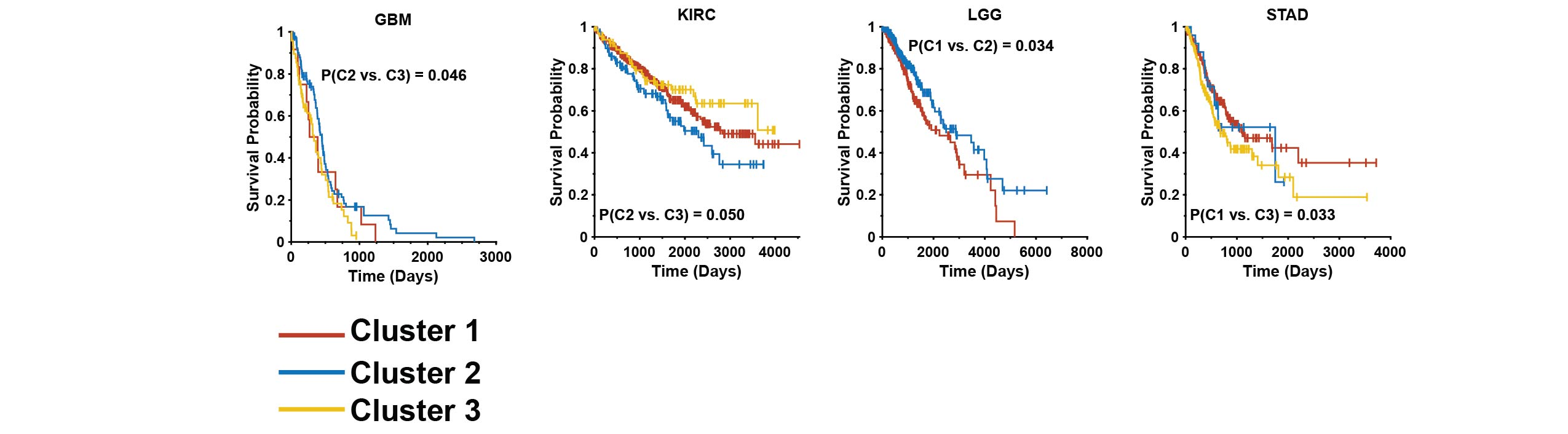


**Figure S19.** Additional Kaplan-Meier survival curves for patients with distinct groups of TP53 Pathway t-SNE clusters, excluding those shown in Fig. 2.

**
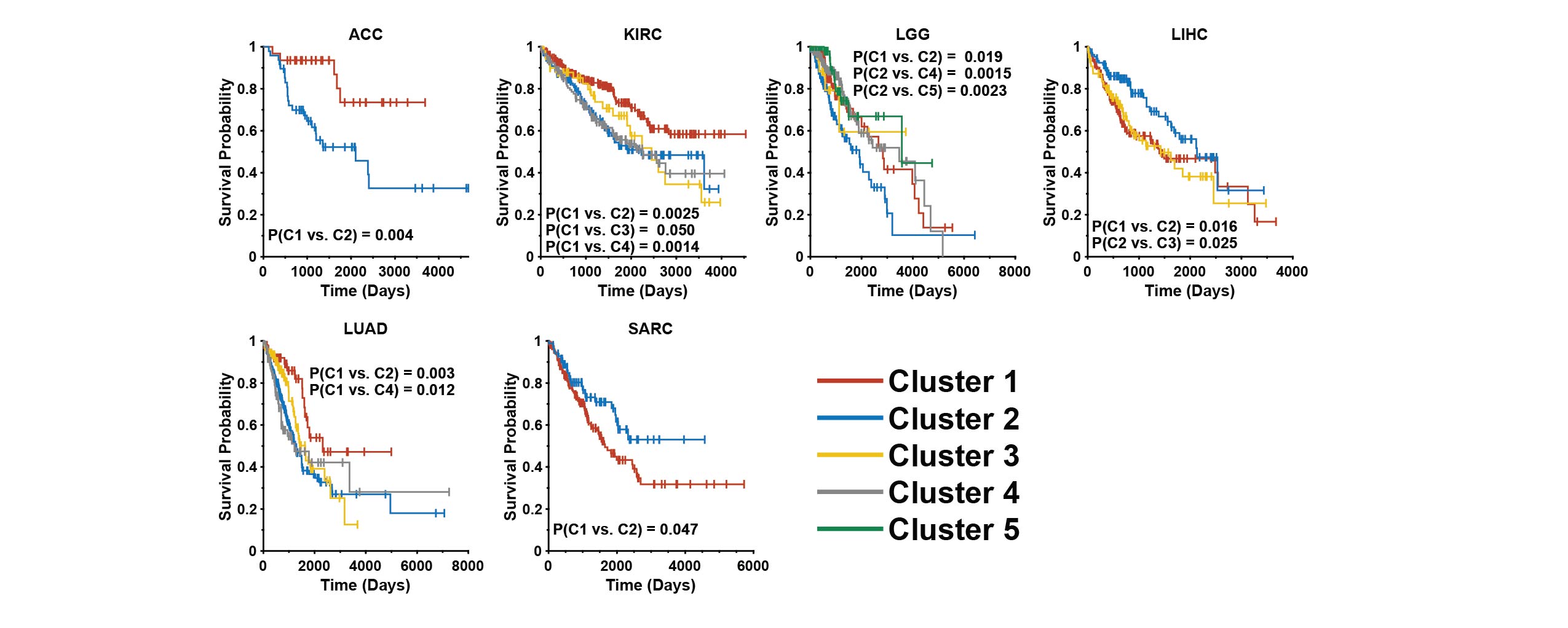
**

**Figure S20.** Additional Kaplan-Meier survival curves for patients with distinct groups of TGF-β Pathway t-SNE clusters, excluding those shown in Fig. 2.


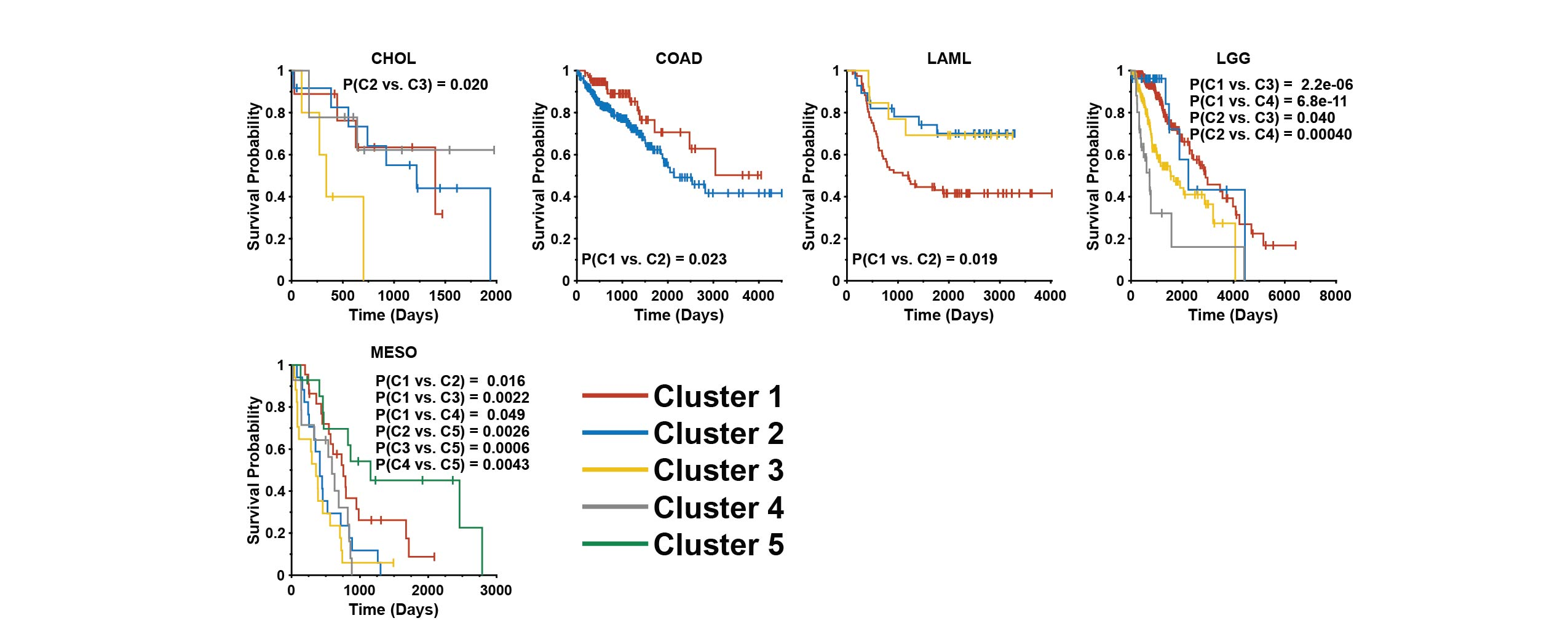


**Figure S21.** Additional Kaplan-Meier survival curves for patients with distinct groups of Hippo Pathway t-SNE clusters, excluding those shown in Fig. 2.


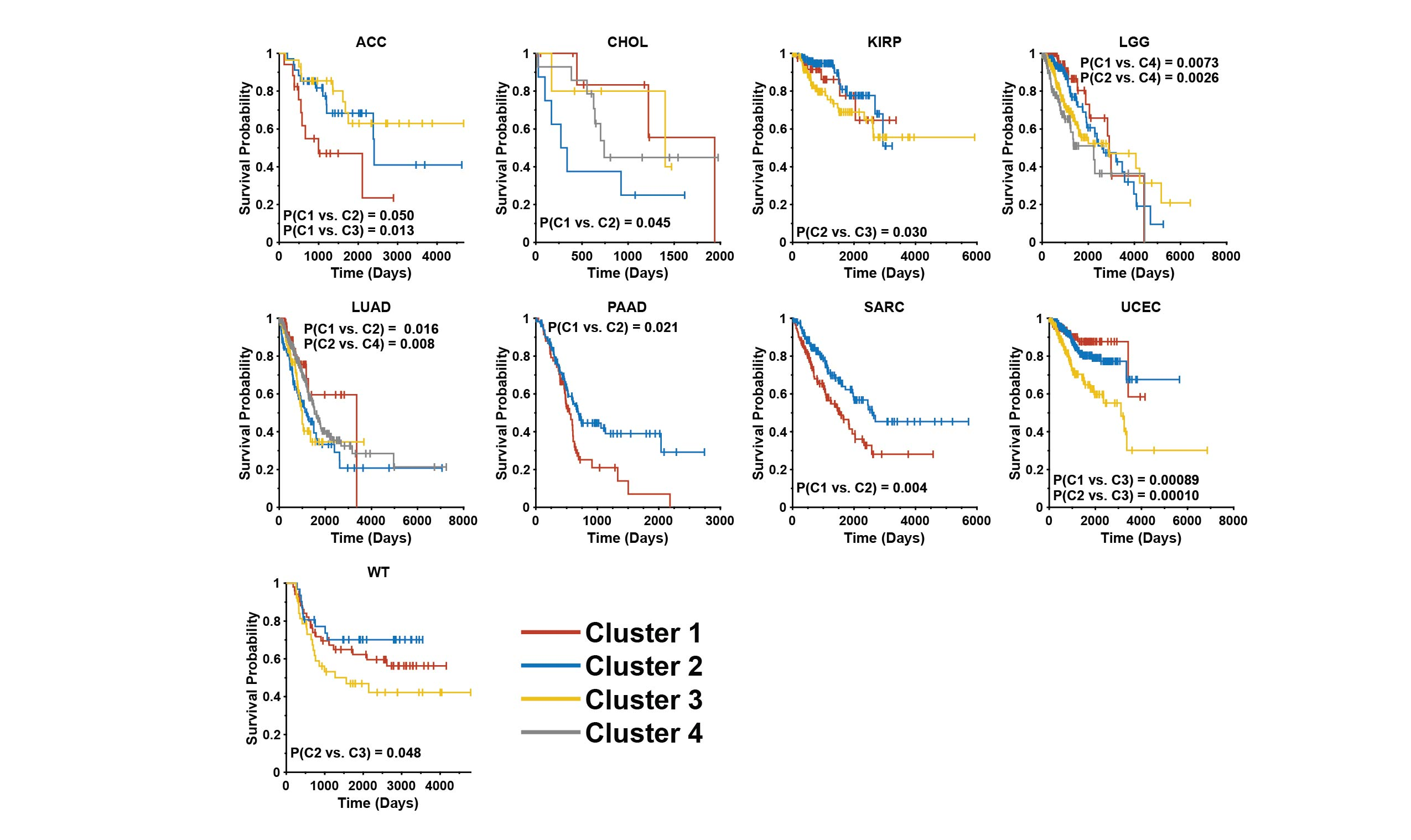


**Figure S22.** Additional Kaplan-Meier survival curves for patients with distinct groups of Myc Pathway t-SNE clusters, excluding those shown in Fig. 2.

**
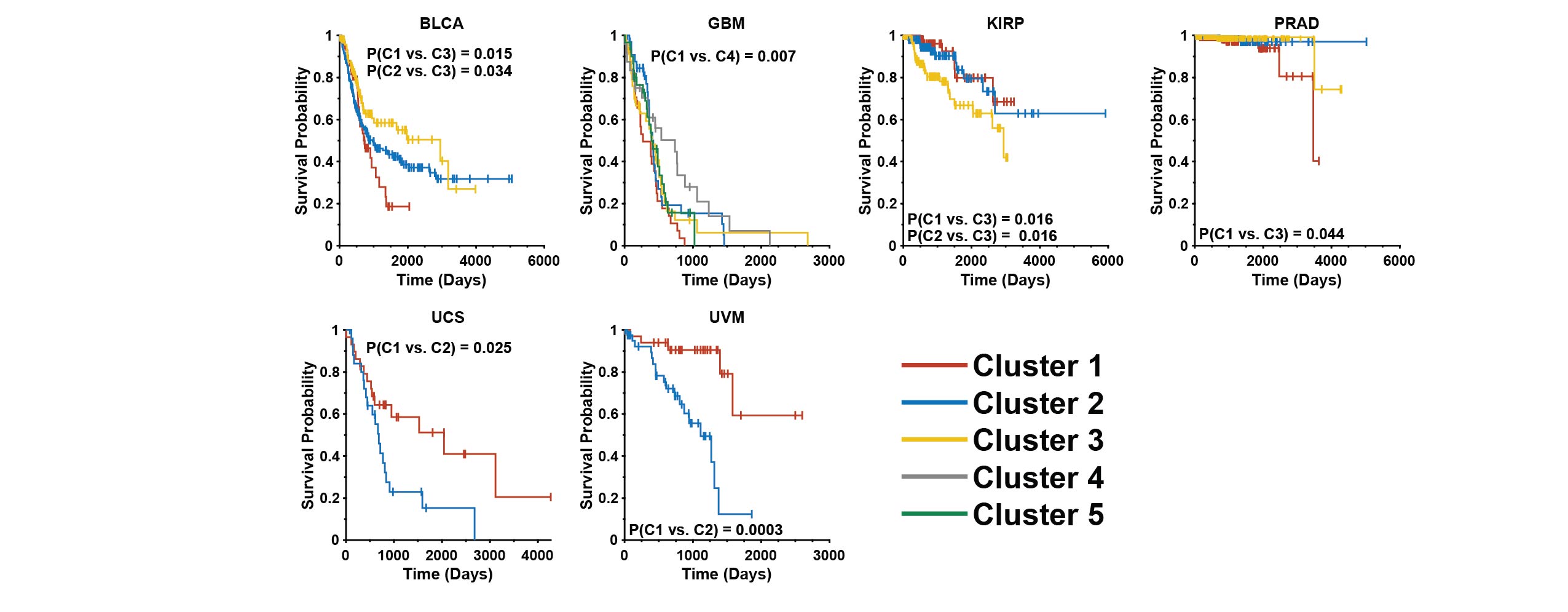
**

**Figure S23.** Additional Kaplan-Meier survival curves for patients with distinct groups of TCA Cycle Pathway t-SNE clusters, excluding those shown in Fig. 2.


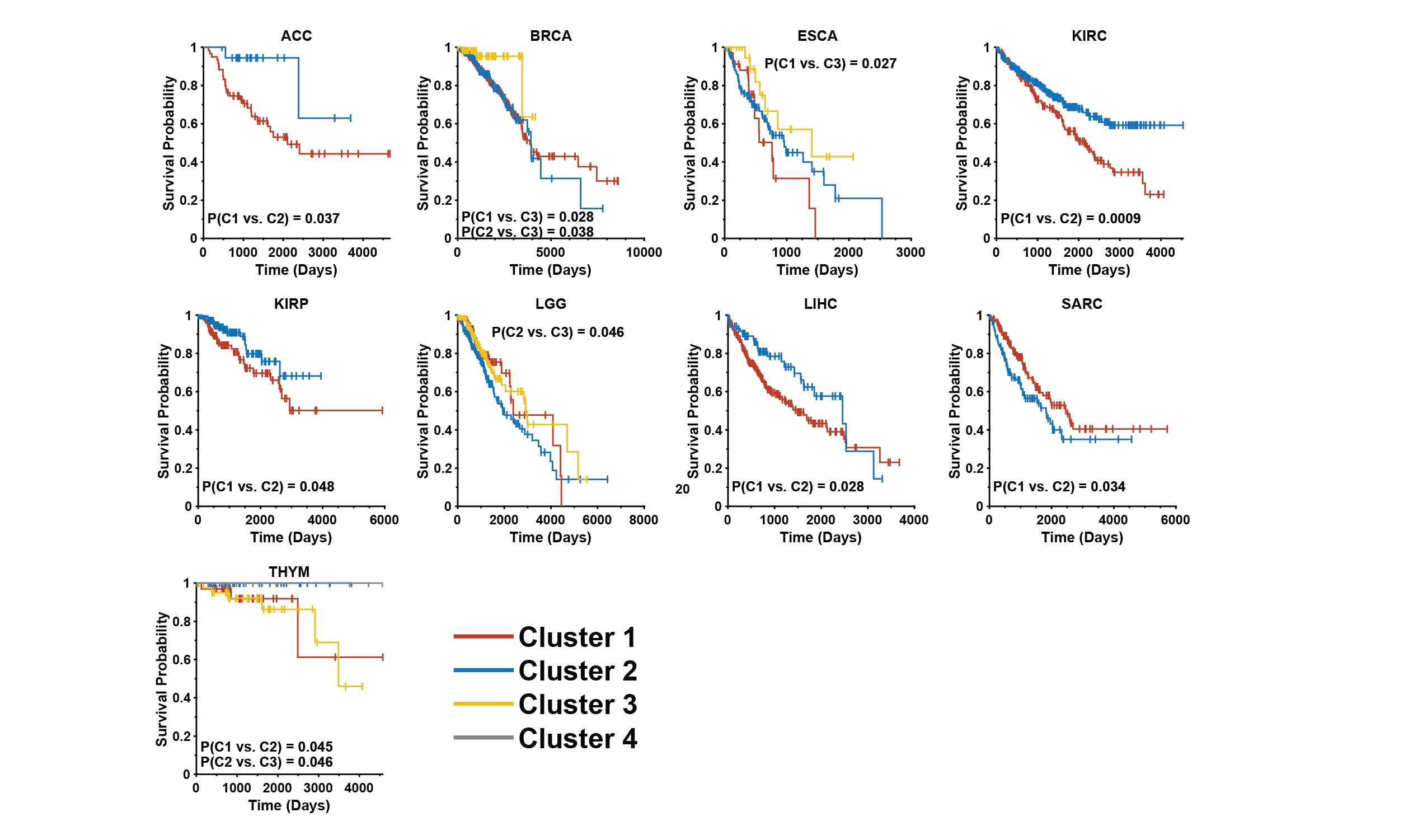


**Figure S24.** Additional Kaplan-Meier survival curves for patients with distinct groups of Pentose Phosphate Pathway t-SNE clusters, excluding those shown in Fig. 2.

**
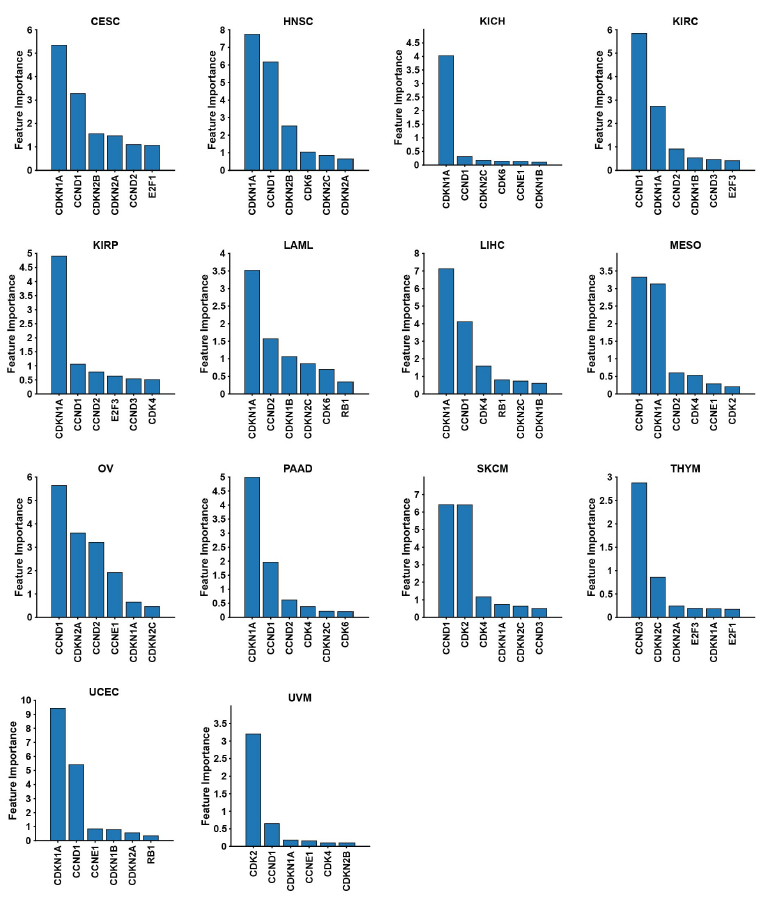
**

**Figure S25.** Additional Random Forest Classifiers showing the individual transcripts in the Cell Cycle Pathway that were most deterministic of t-SNE profiles for each relevant tumor type.


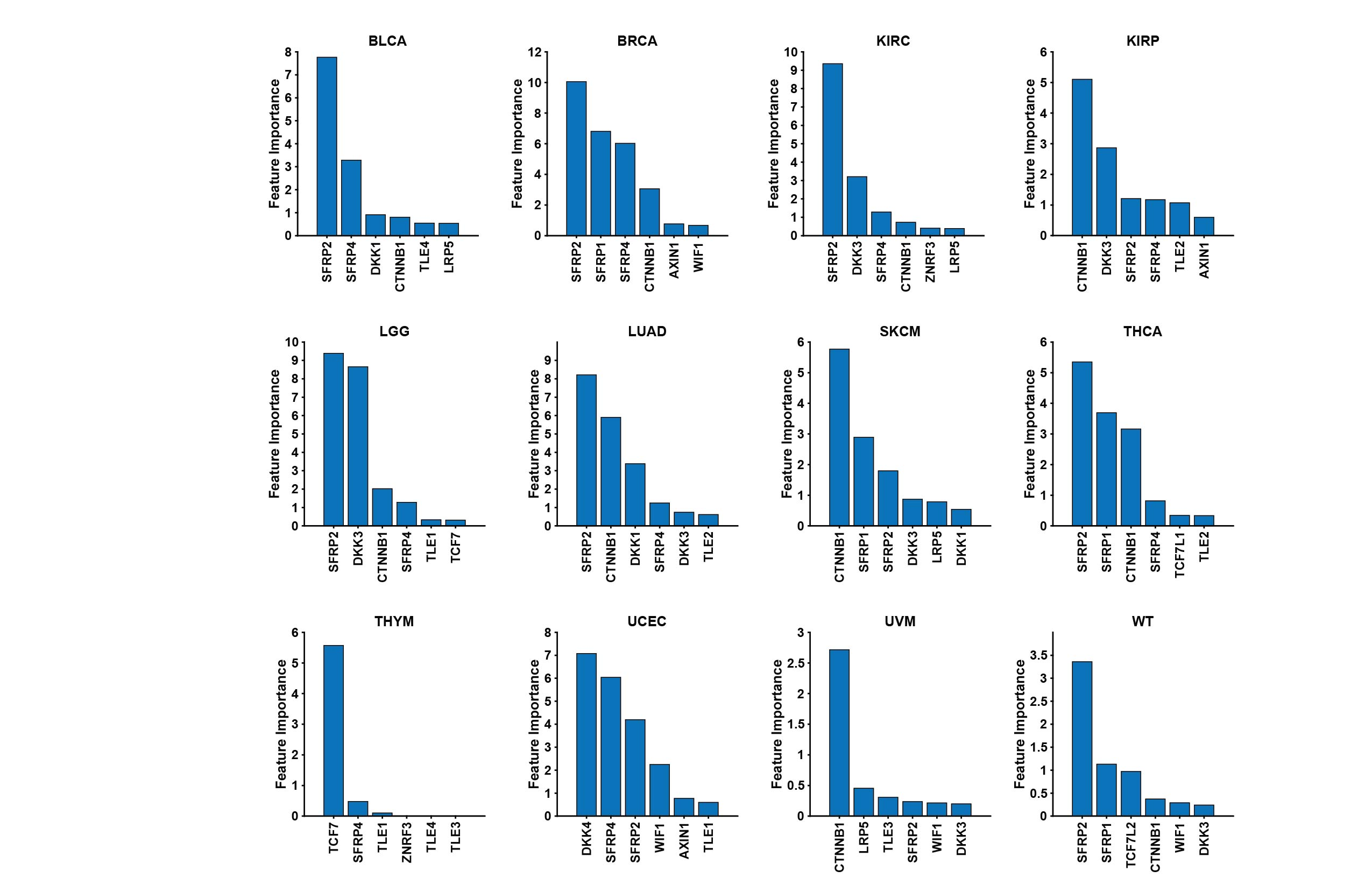


**Figure S26.** Additional Random Forest Classifiers showing the individual transcripts in the Wnt Pathway that were most deterministic of t-SNE profiles for each relevant tumor type.

**
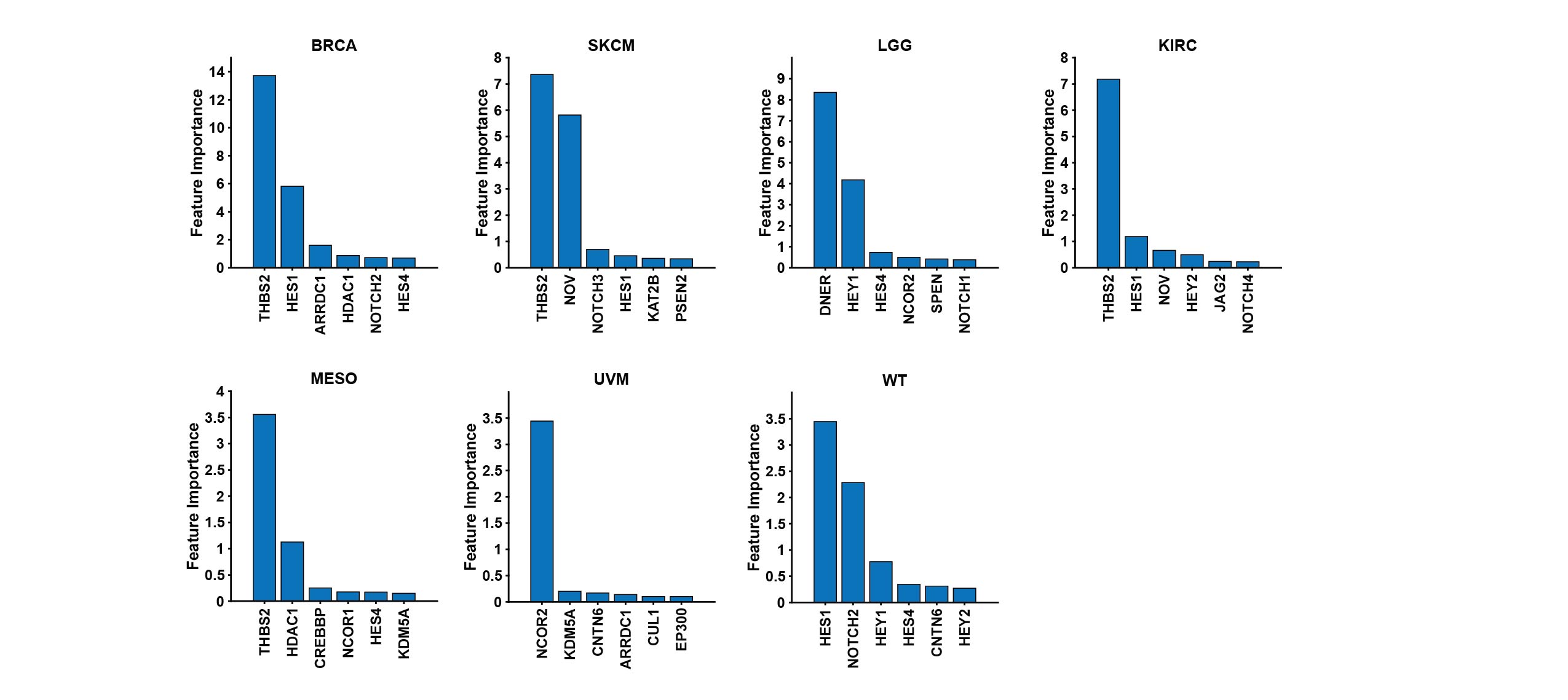
**

**Figure S27.** Additional Random Forest Classifiers showing the individual transcripts in the Notch Pathway that were most deterministic of t-SNE profiles for each relevant tumor type.


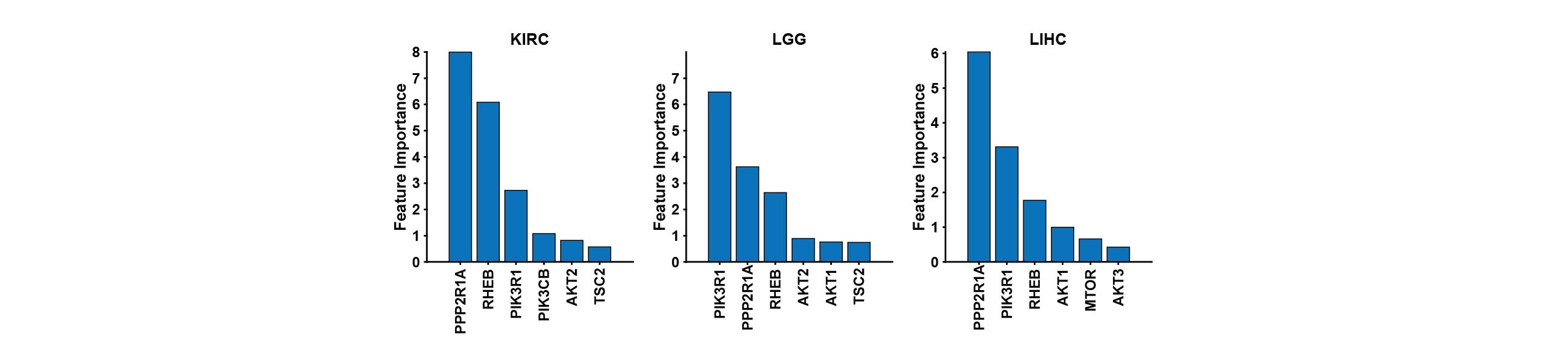


**Figure S28.** Additional Random Forest Classifiers showing the individual transcripts in the PI3K Pathway that were most deterministic of t-SNE profiles for each relevant tumor type.

**
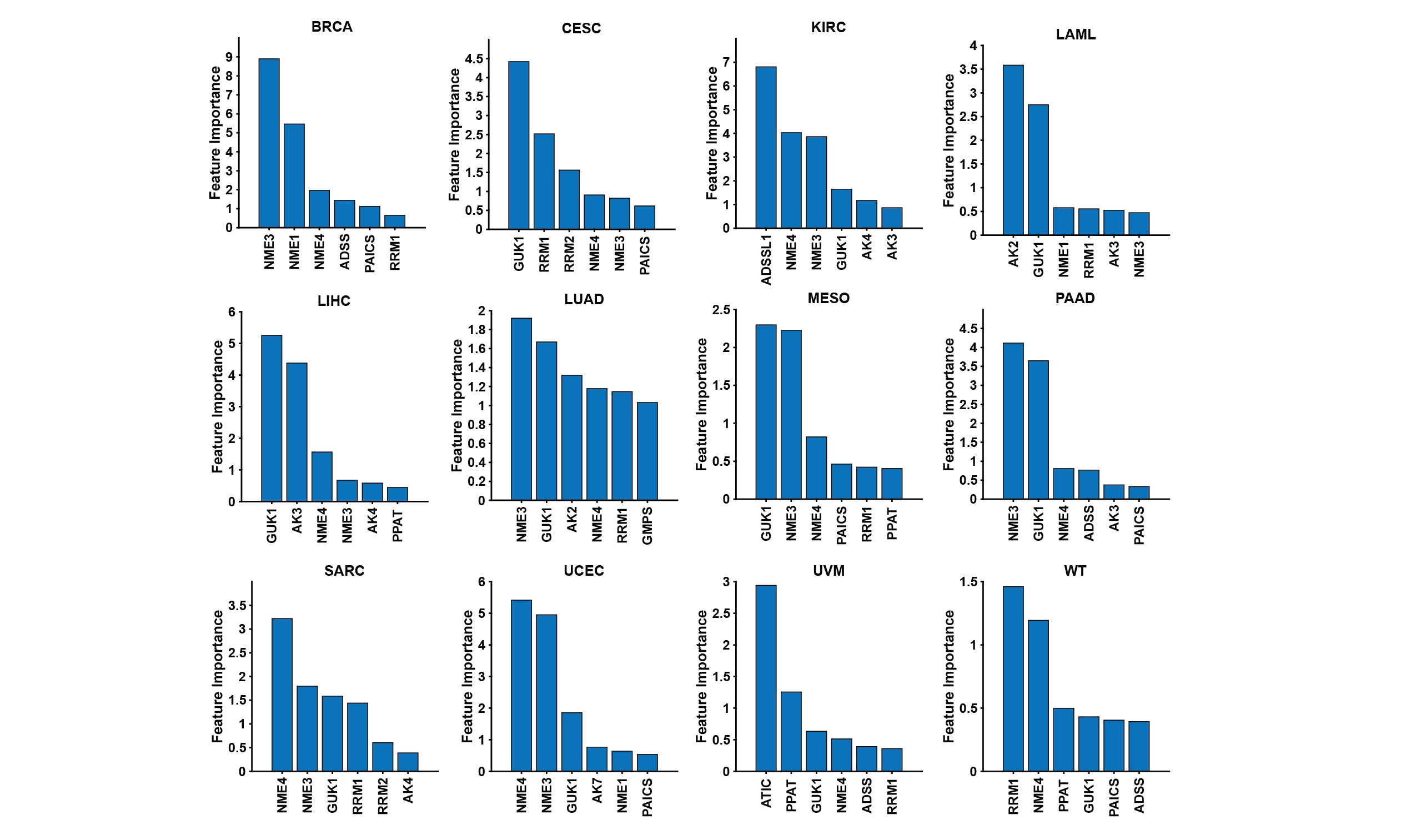
**

**Figure S29.** Additional Random Forest Classifiers showing the individual transcripts in the Purine Biosynthesis Pathway that were most deterministic of t-SNE profiles for each relevant tumor type.

**
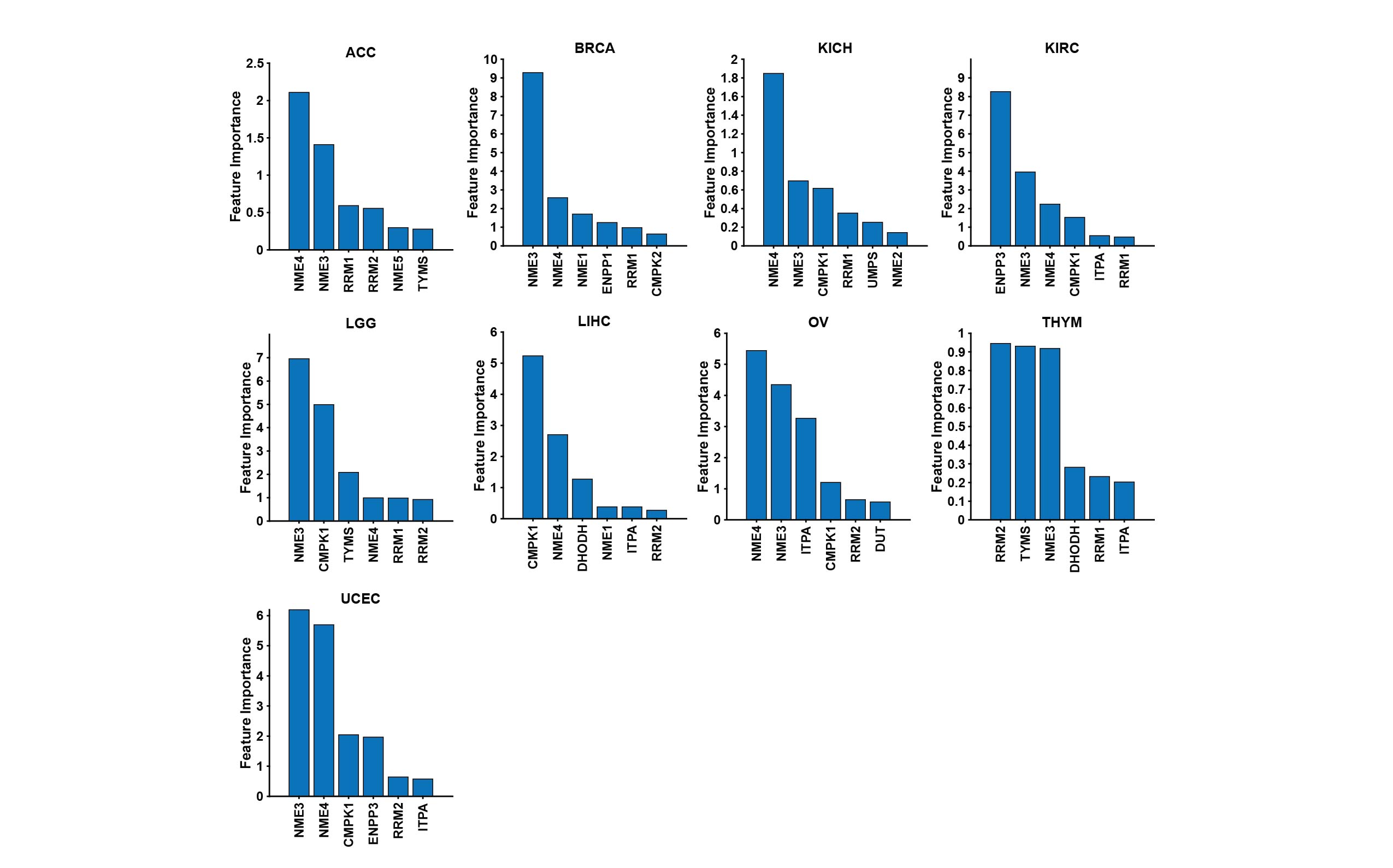
**

**Figure S30.** Additional Random Forest Classifiers showing the individual transcripts in the Pyrimidine Biosynthesis Pathway that were most deterministic of t-SNE profiles for each relevant tumor type.


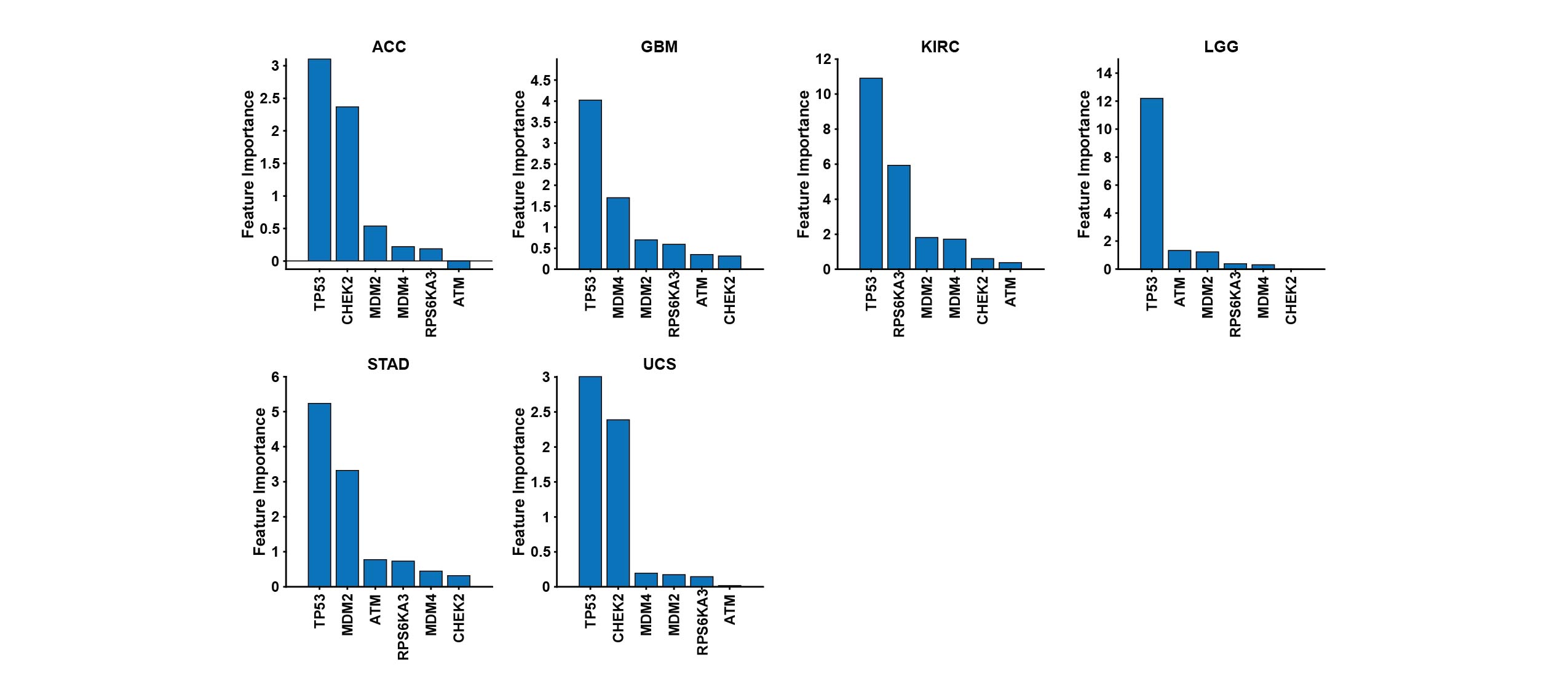


**Figure S31.** Additional Random Forest Classifiers showing the individual transcripts in the TP53 Pathway that were most deterministic of t-SNE profiles for each relevant tumor type.

**
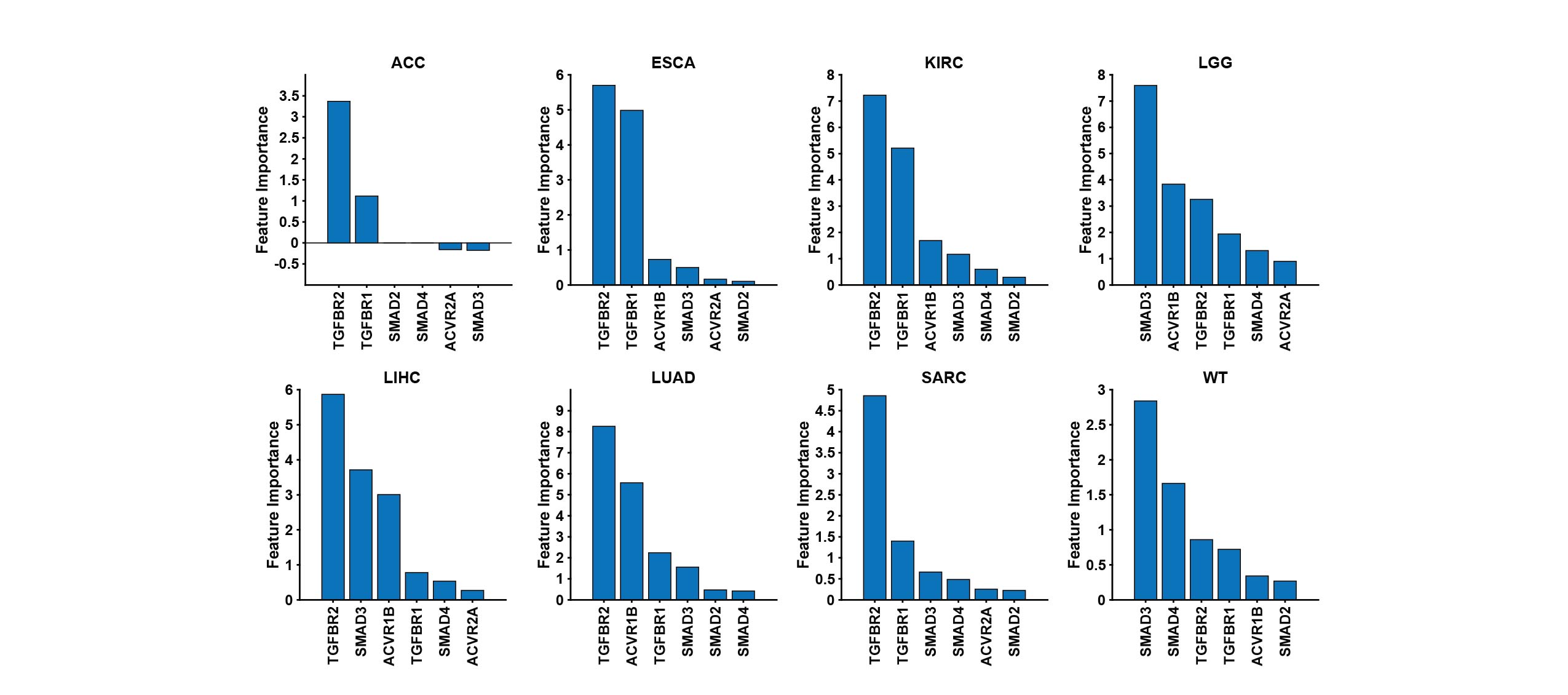
**

**Figure S32.** Additional Random Forest Classifiers showing the individual transcripts in the TGF-β Pathway that were most deterministic of t-SNE profiles for each of 11 relevant tumor types, not including those shown in Fig. 4.

**
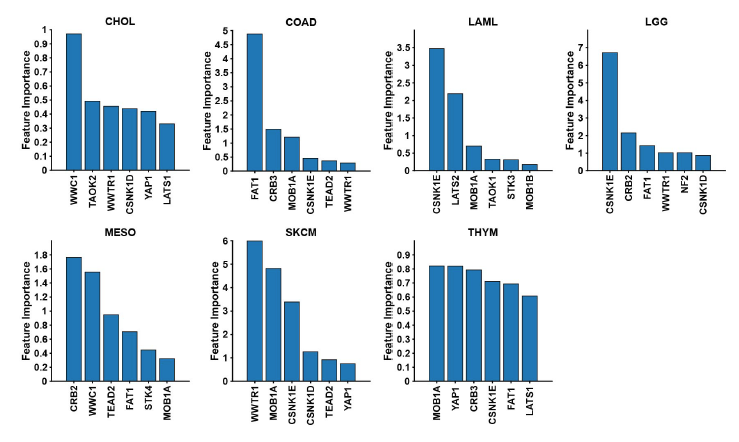
**

**Figure S33.** Additional Random Forest Classifiers showing the individual transcripts in the Hippo Pathway that were most deterministic of t-SNE profiles for each relevant tumor type.

**
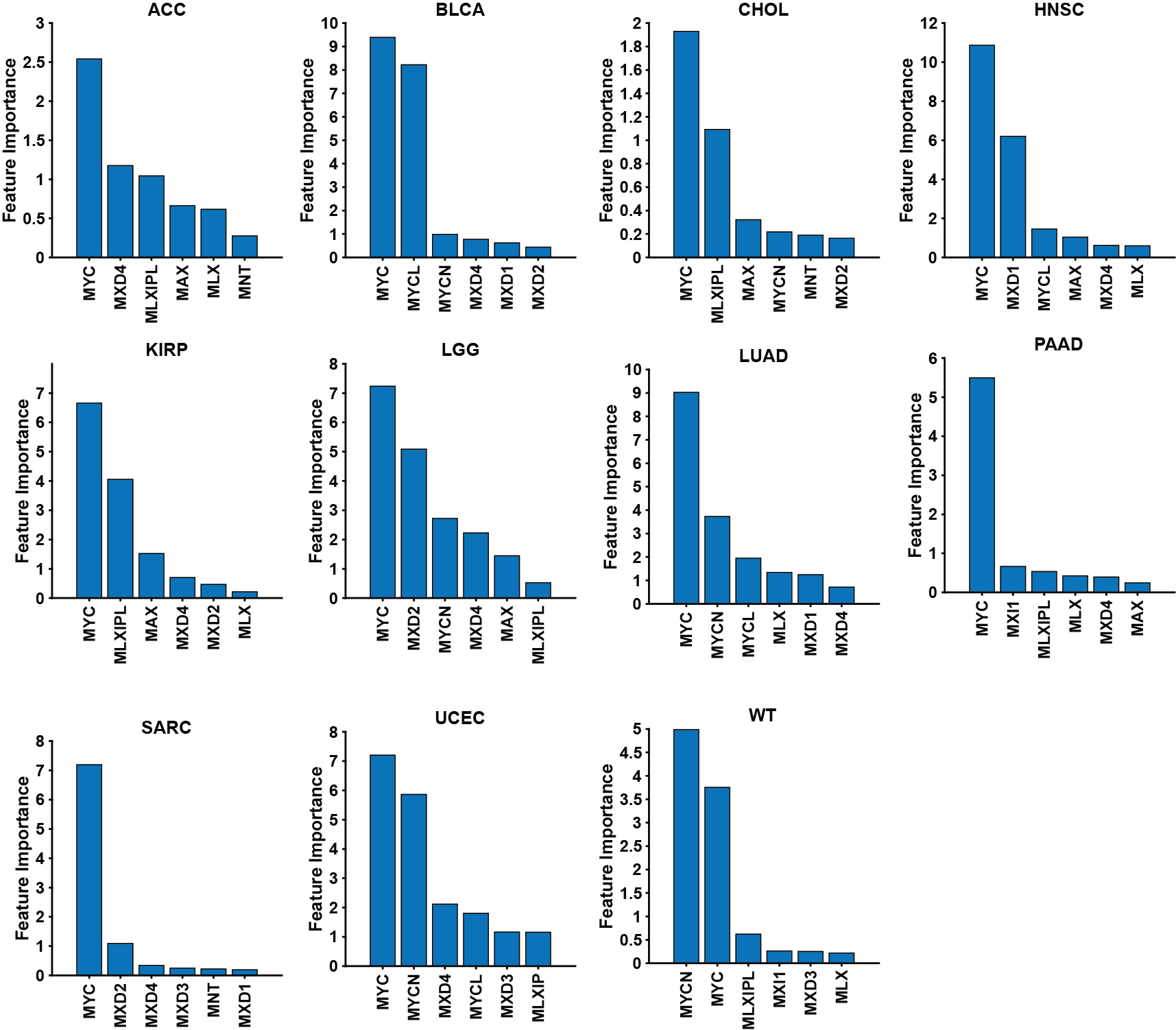
**

**Figure S34.** Additional Random Forest Classifiers showing the individual transcripts in the Myc Pathway that were most deterministic of t-SNE profiles for each relevant tumor type.


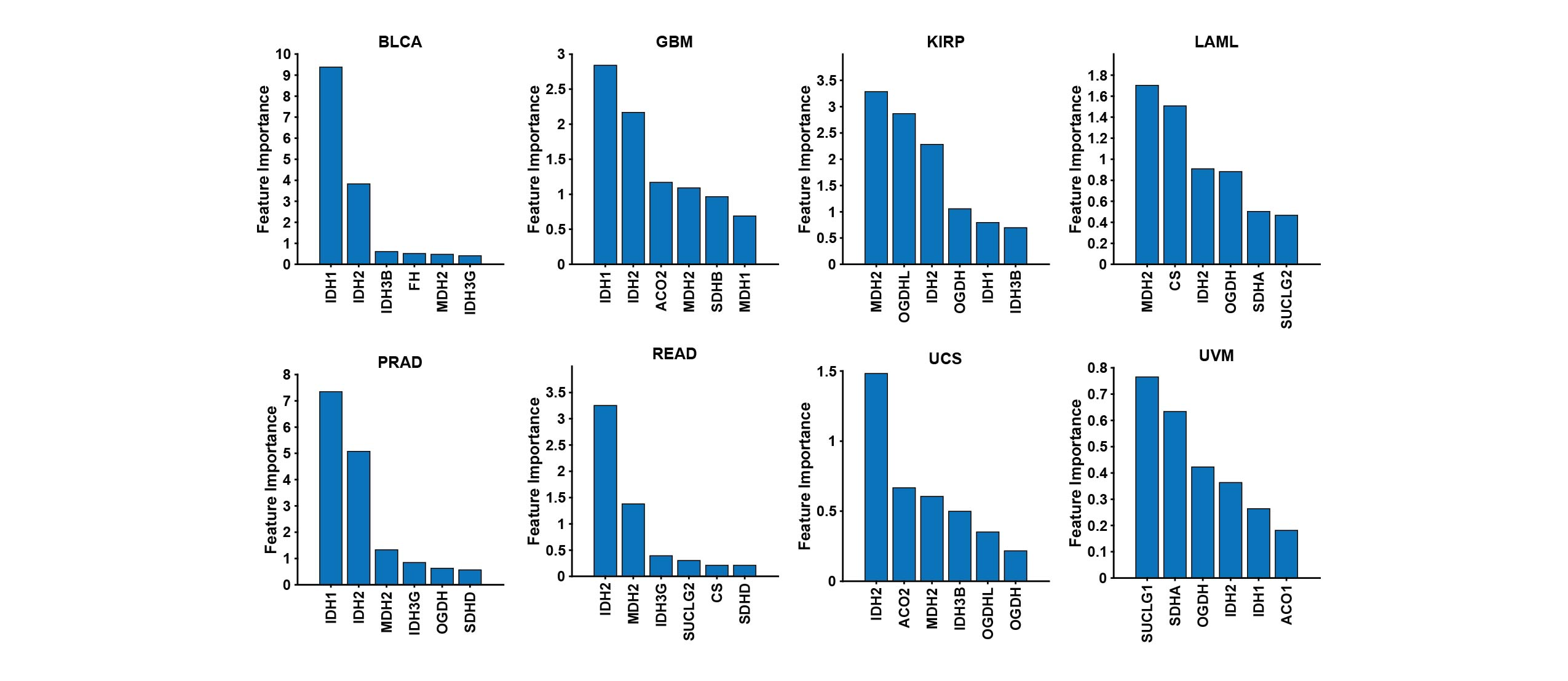


**Figure S35.** Additional Random Forest Classifiers showing the individual transcripts in the TCA Pathway that were most deterministic of t-SNE profiles for each relevant tumor type.


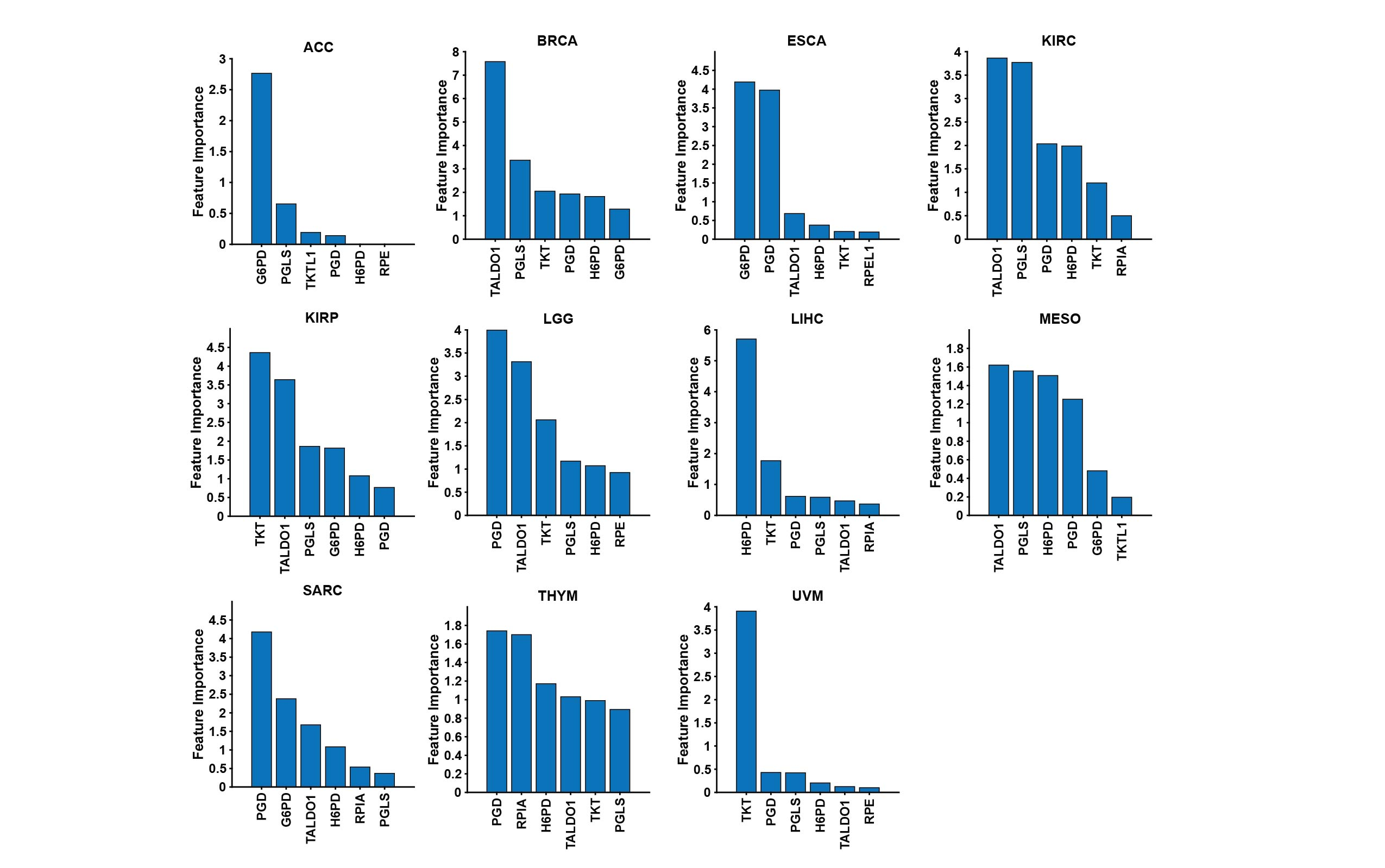


**Figure S36.** Additional Random Forest Classifiers showing the individual transcripts in the Pentose Phosphate Pathway that were most deterministic of t-SNE profiles for each relevant tumor type.


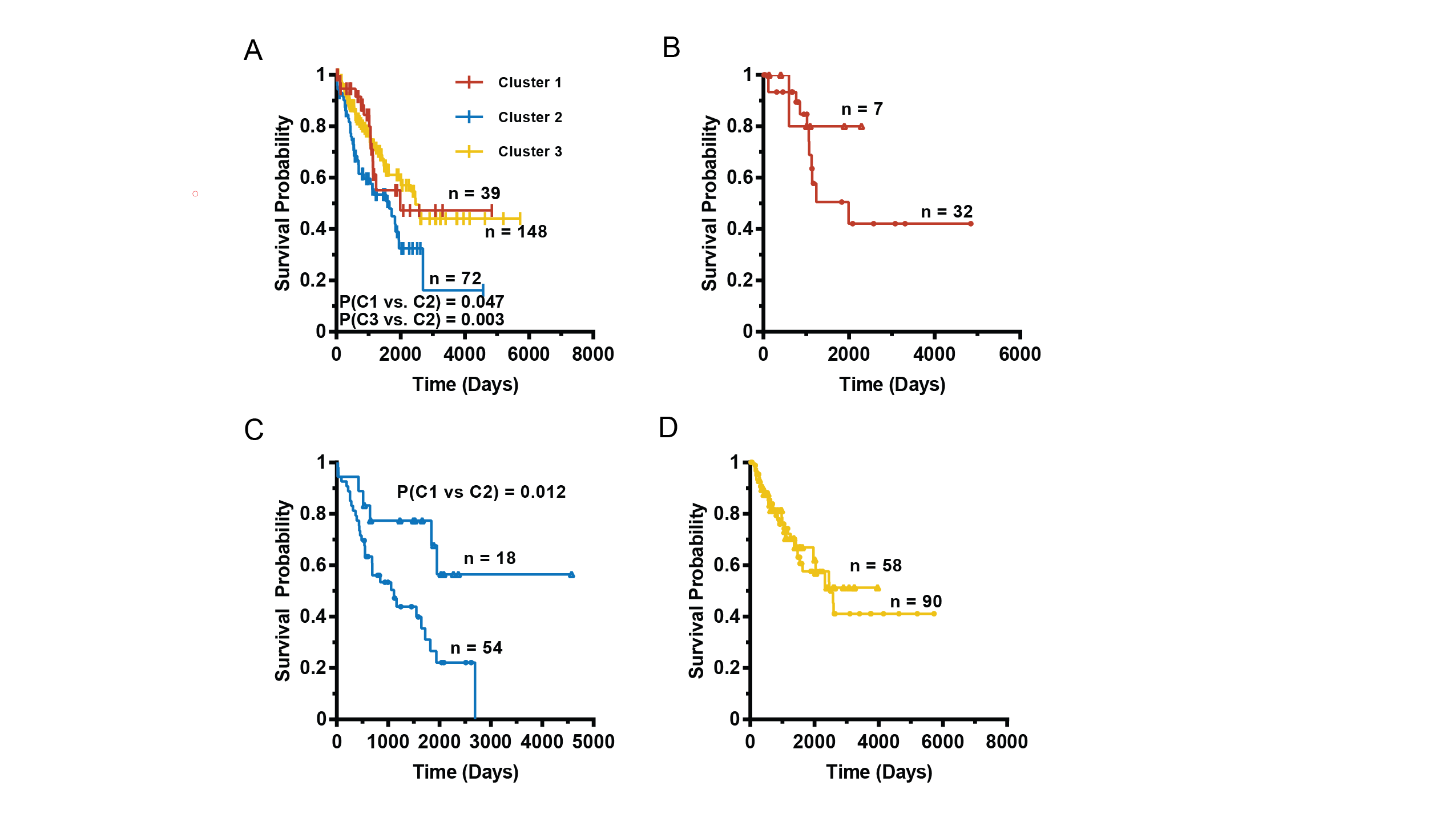


**Figure S37.** Additional predictive power of sequential t-SNE analyses. (A). The survival curve shown in Fig. 2 of sarcoma patients based on t-SNE clusters from the Purine Biosynthesis Pathway. (B). Cluster 1 patients from A were further analyzed based on whether they could be categorized as Cluster 1 or Cluster 2 when analyzed for TGF-β Pathway transcripts. (C). Cluster 2 patients from A were similarly categorized as in B. (D). Cluster 3 patients from A were similarly categorized as in B. (4). Cluster 4 patients from A were similarly categorized as in B.


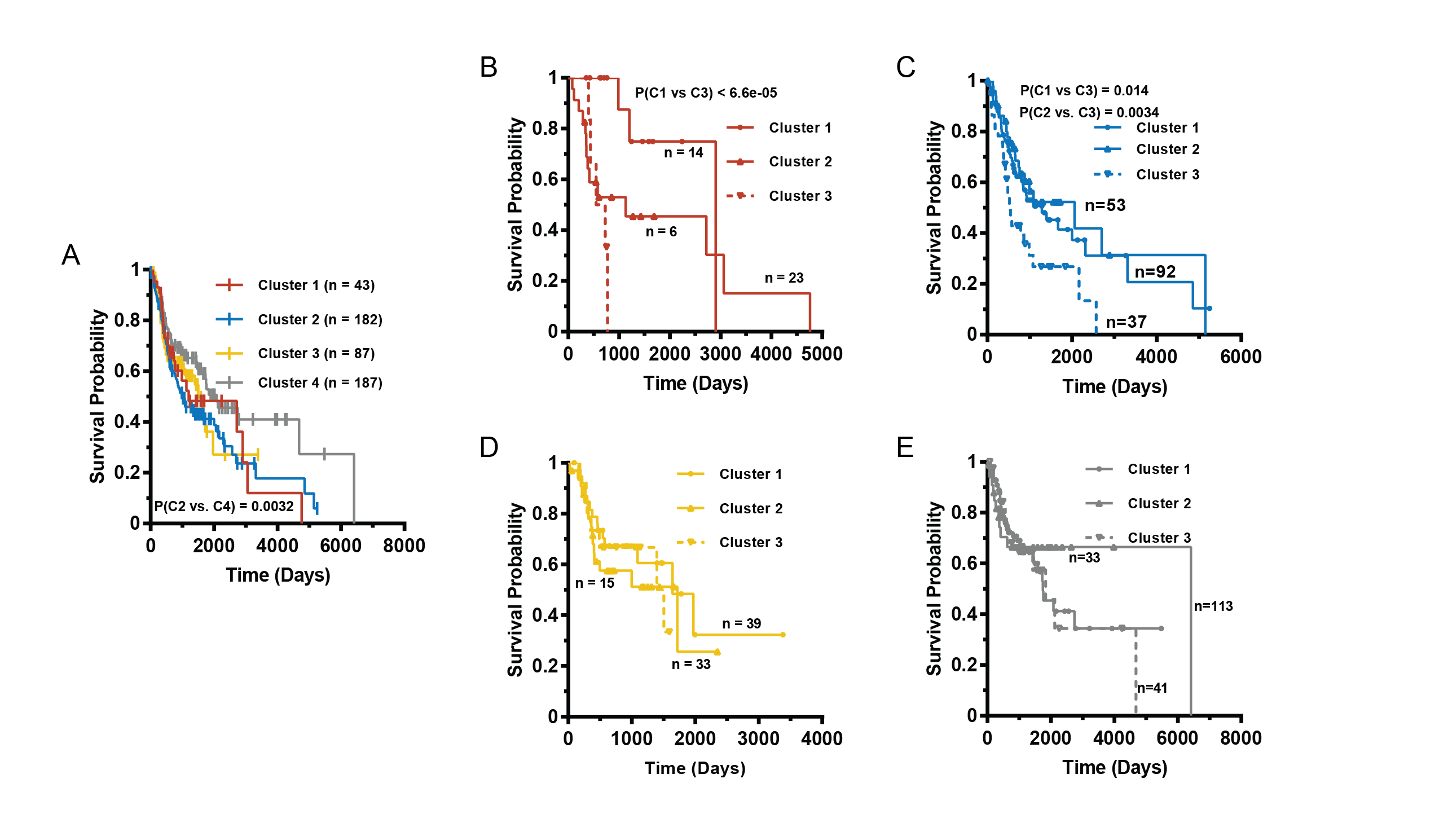


**Figure S38.** Additional predictive power of sequential t-SNE analyses. (A). The survival curves of head and neck cancer patients based on t-SNE clusters from the Myc Pathway taken from Fig. 2 in the Supplementary Appendix. (B). Cluster 1 patients from A were further analyzed based on whether they could be categorized as Cluster 1, Cluster 2, or cluster 3 when analyzed for cell cycle Pathway transcripts. (C-E). Clusters 2-4 patients from A were similarly categorized as in B. Figs.


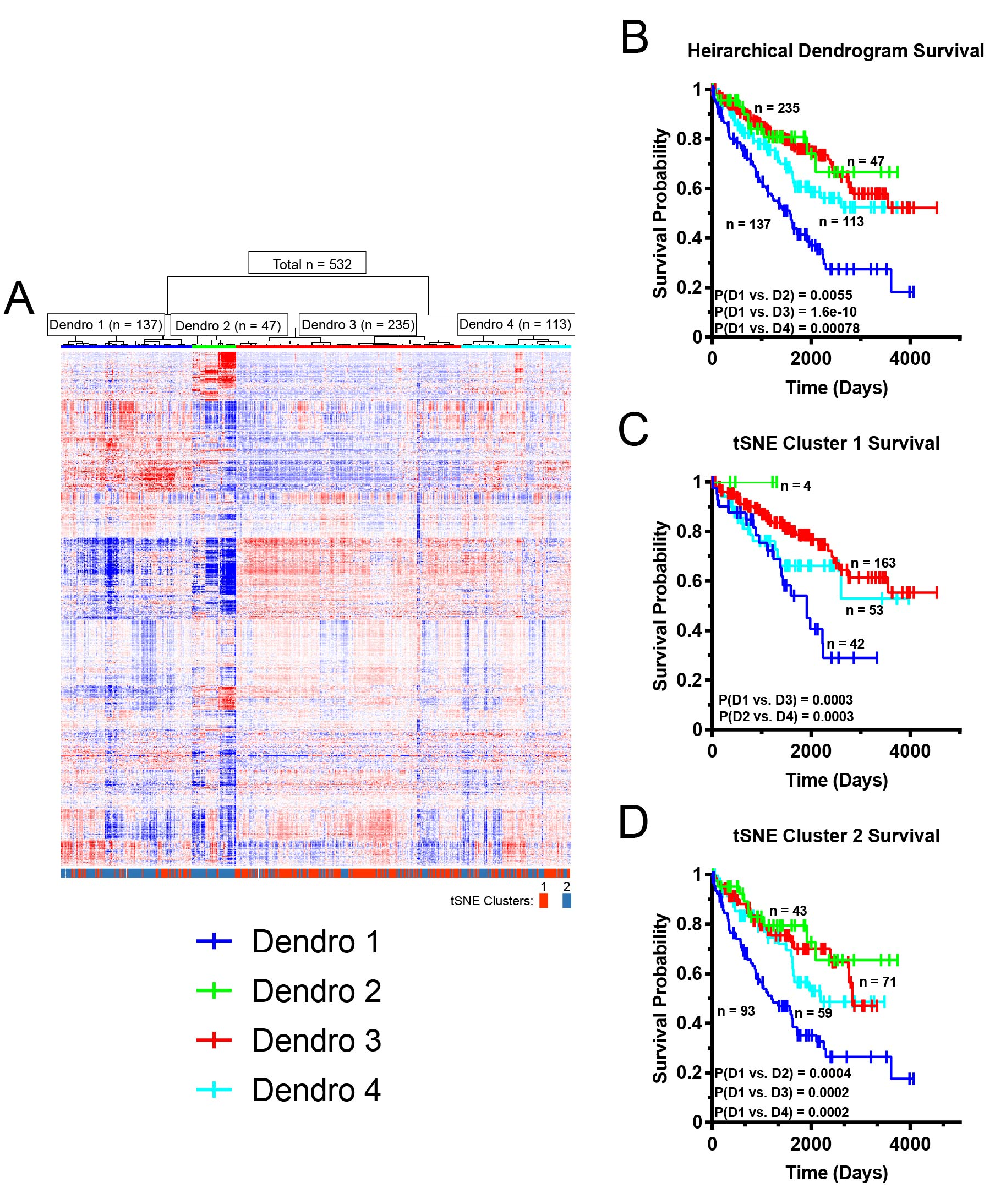


**Figure S39.** Whole transcriptome analysis refines the predictive power of Pyrimidine Pathway t-SNE profiling in renal clear cell carcinoma (KIRC). (A). Hierarchical clustering of all KIRCs based on whole transcriptome profiling. Each tumor’s t-SNE cluster is indicated and is derived from Fig. 2. (B). Kaplan-Meier survival curves of each of the Dendro groups from A. (C). All t-SNE Cluster 1 tumors with favorable survival (Fig. 2) were further categorized based on their Dendro Groupings. It can be seen that these tumors were associated with a worse overall survival if they fell into the Dendro 1 group. Similarly, t-SNE cluster 2 tumors with overall unfavorable survival could be further sub-classified according to their Dendro group.


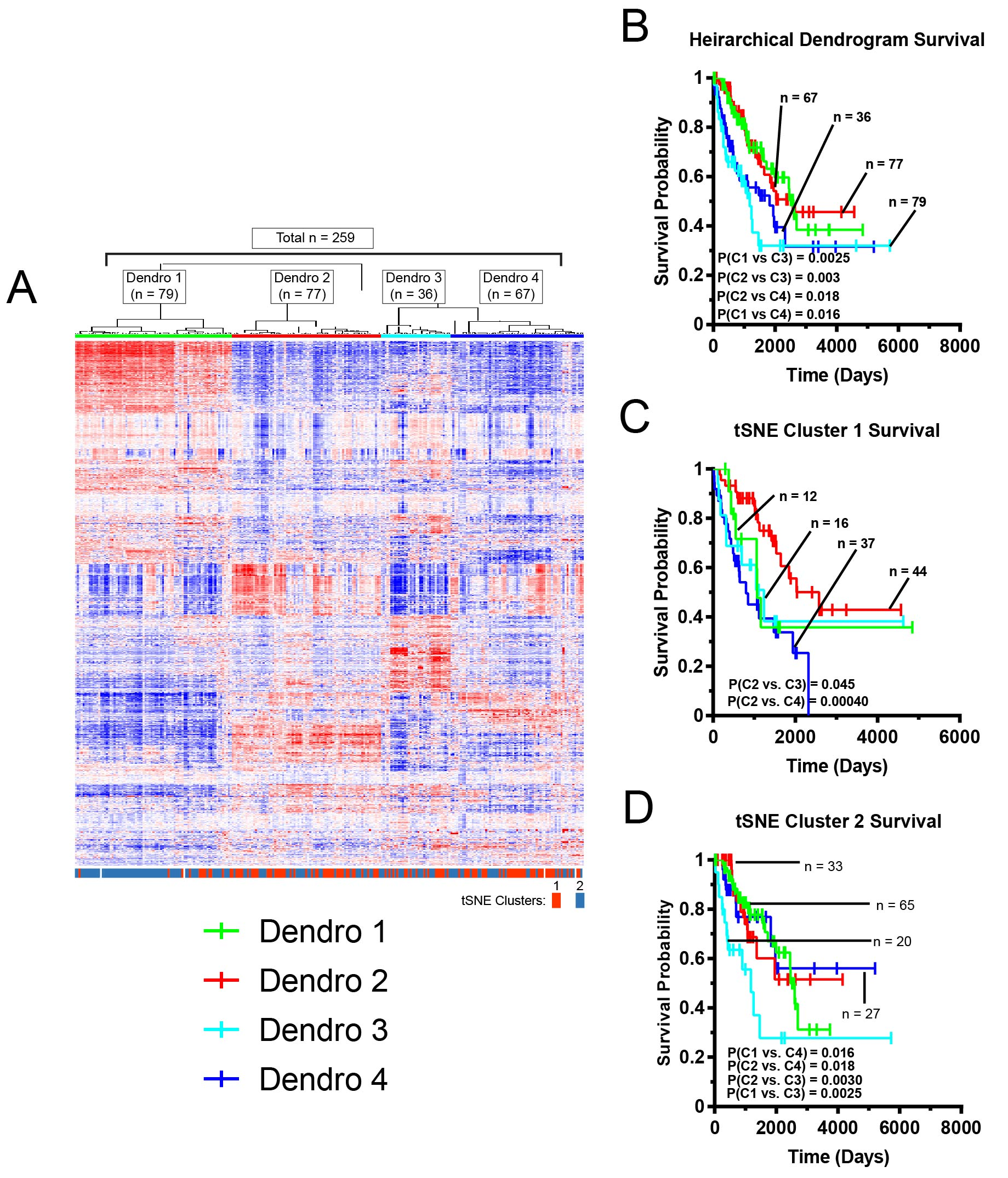


**Figure S40.** Whole transcriptome analysis refines the predictive power of Myc Pathway t-SNE profiling in sarcoma (SARC). (A). Hierarchical clustering of all sarcoma patients identified 4 distinct Dendro Groups (1-4). The two t-SNE Clusters into which these tumors fell are indicated at the bottom of the heat map. Note that the Dendro 1 Group is particularly weighted with t-SNE Cluster 2 tumors having favorable survival. To a somewhat lesser extent, the Dendro 4 Group was more heavily populated by t-SNE Cluster 1 tumors with unfavorable survival. (B). Survival for each of the Dendro Groups in (A) showing that Dendro Groups 1 and 2 were associated with relatively favorable survival whereas Dendro group 4 was associated with unfavorable survival. (C). t-SNE Cluster 1 unfavorable survival tumors could be further subdivided based on their Dendro Group identities. (D). t-SNE Cluster 2 favorable survival tumors could also be subdivided further based on there whole transcriptome profiles.


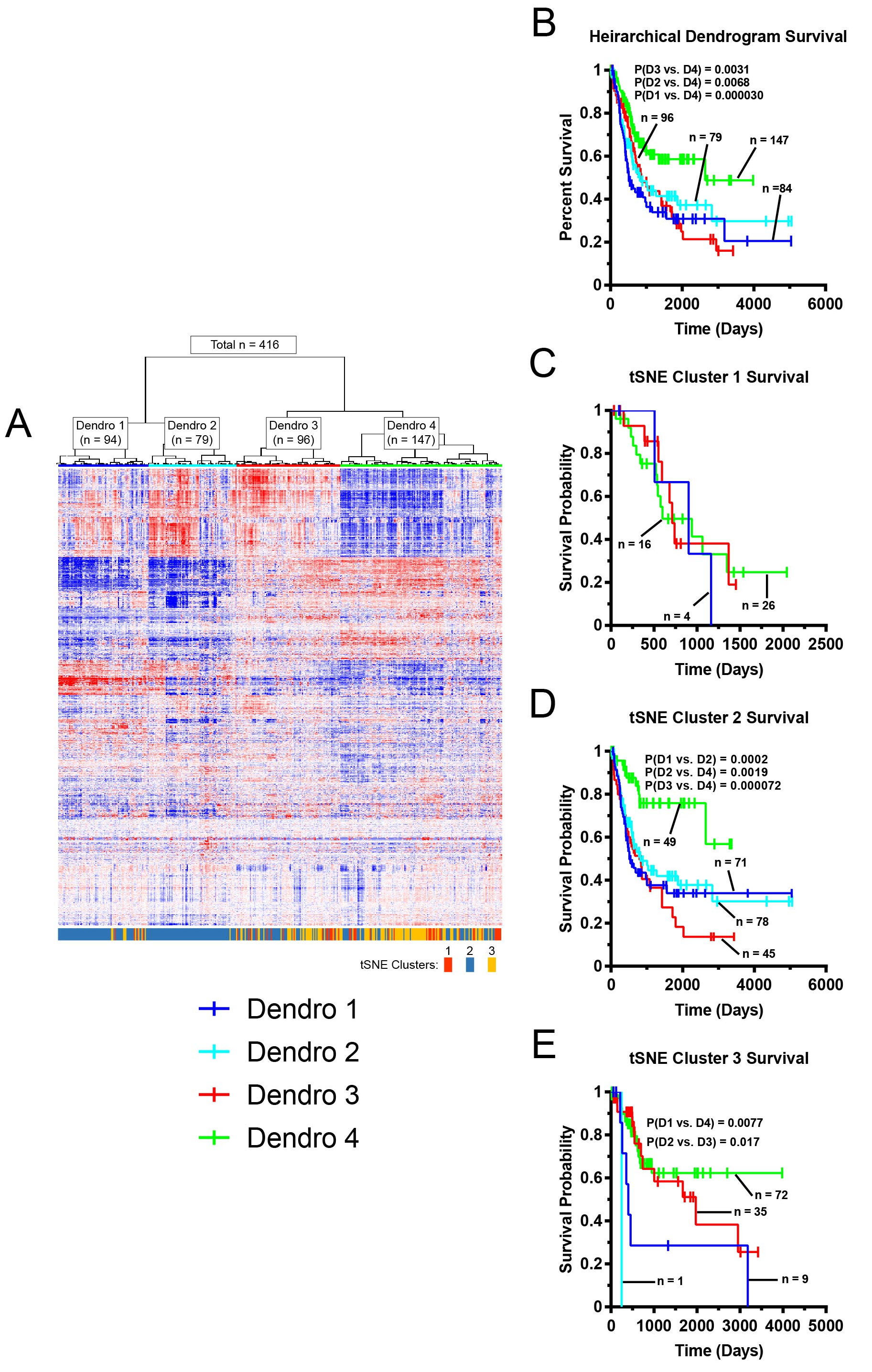


**Figure S41.** Whole transcriptome analysis refines the predictive power of TCA Cycle Pathway in bladder urothelial cancer (BLCA). (A). Hierarchical cluster of all tumors identified 4 Dendro Groups. Note that Dendro Groups 1 and 2 are over-represented by t-SNE Cluster 2 TCA Pathway tumors with an intermediate survival whereas Dendro Group 4 is over-represented by t-SNE Cluster 3 tumors with a relatively favorable survival (Figures S11 and S23 in the Supplementary Appendix). (B). Kaplan-Meier survival curves of each of the 4 Dendro Groups in (A). (C-E). Kaplan-Meier survival curves of each of the 3 t-SNE Groups. Note that the t-SNE Cluster 1 could not be further subdivided by further hierarchical clustering whereas both t-SNE Clusters 2 and 3 could.
